# Psychometrics of health-related quality of life questionnaires in bronchiectasis: a systematic review and meta-analysis

**DOI:** 10.1183/13993003.00025-2021

**Published:** 2021-11-11

**Authors:** Rebecca H. McLeese, Arietta Spinou, Zina Alfahl, Michail Tsagris, J. Stuart Elborn, James D. Chalmers, Anthony De Soyza, Michael R. Loebinger, Surinder S. Birring, Konstantinos C. Fragkos, Robert Wilson, Katherine O'Neill, Judy M. Bradley

**Affiliations:** 1 The Wellcome Trust-Wolfson Northern Ireland Clinical Research Facility, School of Medicine, Dentistry and Biomedical Sciences, Queen's University Belfast, Belfast, UK; 2 Population Health Sciences, Faculty of Life Sciences and Medicine, King's College London, London, UK; 3 School of Pharmacy, Queen's University Belfast, Belfast, UK; 4 Dept of Economics, University of Crete, Rethymnon, Greece; 5 Wellcome-Wolfson Institute for Experimental Medicine, School of Medicine, Dentistry and Biomedical Sciences, Queen's University Belfast, Belfast, UK; 6 Scottish Centre for Respiratory Research, University of Dundee, Dundee, UK; 7 Respiratory Dept, Institute of Cellular Medicine, Newcastle University and Freeman Hospital, Sir William Leech Research Centre, Newcastle upon Tyne, UK; 8 Host Defence Unit, Royal Brompton Hospital, London, UK; 9 Centre for Human and Applied Physiological Sciences, School of Basic and Medical Biosciences, Faculty of Life Sciences and Medicine, King's College London, London, UK; 10 Division of Medicine, University College London, London, UK; 11 These two authors are co-senior authors

## Abstract

**Introduction:**

Understanding the psychometric properties of health-related quality of life (HRQoL) questionnaires can help inform selection in clinical trials. Our objective was to assess the psychometric properties of HRQoL questionnaires in bronchiectasis using a systematic review and meta-analysis of the literature.

**Methods:**

A literature search was conducted. HRQoL questionnaires were assessed for psychometric properties (reliability, validity, minimal clinically important difference (MCID) and floor/ceiling effects). Meta-analyses assessed the associations of HRQoL with clinical measures and responsiveness of HRQoL in clinical trials.

**Results:**

166 studies and 12 HRQoL questionnaires were included. The Bronchiectasis Health Questionnaire (BHQ), Leicester Cough Questionnaire (LCQ), Chronic Obstructive Pulmonary Disease (COPD) Assessment Test (CAT) and Medical Outcomes Study 36-item Short-Form Health Survey (SF-36) had good internal consistency in all domains reported (Cronbach's α≥0.7) across all studies, and the Quality of Life-Bronchiectasis (QOL-B), St George's Respiratory Questionnaire (SGRQ), Chronic Respiratory Disease Questionnaire (CRDQ) and Seattle Obstructive Lung Disease Questionnaire (SOLQ) had good internal consistency in all domains in the majority of (but not all) studies. BHQ, SGRQ, LCQ and CAT had good test–retest reliability in all domains reported (intraclass correlation coefficient ≥0.7) across all studies, and QOL-B, CRDQ and SOLQ had good test–retest reliability in all domains in the majority of (but not all) studies. HRQoL questionnaires were able to discriminate between demographics, important markers of clinical status, disease severity, exacerbations and bacteriology. For HRQoL responsiveness, there was a difference between the treatment and placebo effect.

**Conclusions:**

SGRQ was the most widely used HRQoL questionnaire in bronchiectasis studies and it had good psychometric properties; however, good psychometric data are emerging on the bronchiectasis-specific HRQoL questionnaires QOL-B and BHQ. Future studies should focus on the medium- to long-term test–retest reliability, responsiveness and MCID in these HRQoL questionnaires which show potential in bronchiectasis.

## Abstract 2

**The psychometric properties of health-related quality of life questionnaires should inform the selection of patient-reported outcomes in bronchiectasis clinical trials** https://bit.ly/3wQuSrm

## Introduction

Bronchiectasis is associated with significant morbidity and substantial healthcare utilisation. There are no licensed medications available and current management guidelines have emphasised the importance of randomised controlled trials to direct care [1, 2]. Evidence from recent clinical trials supports the use of some medications, including macrolides, inhaled antibiotics and novel anti-inflammatory therapies [3–5]. However, a number of other clinical trials have failed to demonstrate a clinically important or statistically significant change in their primary end-points, including measures of quality of life (QoL) [6–9]. The reasons for this are multifactorial, including ineffective treatments, lack of consideration of treatable traits, and underlying physiology and microbiome, but also the inability of interventions to affect specific domain scores of health-related QoL (HRQoL) questionnaires and a lack of bronchiectasis-specific, clinically validated outcome measures. The need for robust outcome measures in clinical trials has been emphasised by regulators [10, 11], with particular emphasis on tools used to measure patient-reported outcomes (PROs).

In 2016, Spinou *et al*. [12] published a systematic review summarising data from 57 studies across nine HRQoL questionnaires in bronchiectasis. They concluded that most of the HRQoL questionnaires had good reliability and validity. The responsiveness of HRQoL questionnaires ranged from trivial to large and there were some differences between questionnaires in their association with clinical measures such as demographics, symptoms, disease severity, lung function, bacteriology and healthcare utilisation.

Since the Spinou *et al*. [12] systematic review was completed, and based on a recent search of the literature, 1574 new bronchiectasis publications have been identified with data from a broad range of HRQoL questionnaires. For example, a recent large clinical trial, RESPIRE-1 [13], reported a significant change in the St George's Respiratory Questionnaire (SGRQ) but not in Quality of Life-Bronchiectasis (QOL-B) from baseline to end of treatment; discordance between multiple questionnaires causes significant challenges with regulatory approval. The aim of the current systematic review was to determine the psychometric properties of all HRQoL questionnaires available for use in bronchiectasis and to perform a meta-analysis of responsiveness in randomised controlled trials. These data will assist investigators and clinicians in their selection of HRQoL PROs and inform the design of clinical trials in bronchiectasis.

## Methods

### Protocol and registration

The methods for this systematic review and meta-analysis are described in a protocol registered with the International Prospective Register of Systematic Reviews (PROSPERO: CRD42019146181). Findings were reported according to the PRISMA (Preferred Reporting Items for Systematic Reviews and Meta-Analyses) guidelines [14] and the Scottish Intercollegiate Guidelines Network methodology checklist for systematic reviews and meta-analyses [15].

### Study eligibility criteria

Empirical studies of adult patients (≥18 years old) with bronchiectasis, studies reporting on the psychometric properties of HRQoL, and studies reporting on the association between HRQoL and clinical measures were included. In all studies, the diagnosis of bronchiectasis was established by clinical and/or radiological features. Only studies published in the English language were included. Reviews and protocols were excluded. Inclusion and exclusion criteria are listed in full in supplementary table E1.

The following psychometric properties of HRQoL questionnaires were assessed: reliability (internal consistency and test–retest reliability (including timescale of test–retest)), validity (translational validity and discriminant validity), correlations of HRQoL with clinical measures, responsiveness including effect size and minimal clinically important difference (MCID), floor/ceiling effects, and missing data [16, 17]. The psychometric properties assessed are listed in full in supplementary table E1.

### Data sources and searches

Two authors (R.H.M. and Z.A.) independently searched the following databases: Embase, PubMed, MEDLINE, PsycINFO and the Cochrane Library (for search strategy, see supplementary table E2). Further searches were performed including the names of the identified HRQoL questionnaires. Reviews and references from included studies were manually searched to identify additional studies. The literature search included all studies published between 6 November 2014 and 31 December 2020. Spinou *et al*. [12] reported on all relevant studies published prior to 6 November 2014. All relevant studies from the current literature search and the prior review by Spinou *et al*. [12] were included in our review.

### Study selection, data extraction and quality assessment

R.H.M. and Z.A. independently screened articles by title and abstracts against the inclusion criteria, and full text was subsequently reviewed. Abstracts were considered for inclusion if there was adequate information regarding study methods and results. R.H.M. and Z.A. extracted the data, including: author, year of publication, study aim, sample size, disease aetiology, age, gender, lung function (forced expiratory volume in 1 s (FEV_1_) % predicted), HRQoL questionnaire used and its psychometric properties. The quality of studies was assessed by R.H.M. and Z.A. using a modified tool by Swigris *et al*. [18] (appendix 1 in the supplementary material). Multiple articles from the same study were considered as a single study. The original article was cited in most cases, except when a subsequent publication presented new data and/or performed secondary analyses on the original study data; in this case the data was attributed to the later publication. J.B., K.O.N. and A.S. were consulted to resolve any disagreements. For author roles, see appendix 2 in the supplementary material.

### Statistical analysis

Quantitative analysis was performed with R version 3.6.3 (R Foundation for Statistical Computing, Vienna, Austria). Internal consistency was reported as Cronbach's α coefficient, with values ≥0.7 considered acceptable [19]. Test–retest reliability was reported with intraclass correlation coefficient (ICC), with values ≥0.7 considered acceptable for reproducibility [19, 20].

Meta-analysis was performed to assess associations between HRQoL questionnaires and clinical measures. Correlation coefficients (r) were extracted from the collected studies whenever this information was supplied. In cases where only p-values or test statistics (t-values, Cohen's d, F-values or Chi-squared values) were available, correlation coefficients were extracted according to formulas suggested by Rosenthal *et al*. [21]. The strength of association was assessed using the same categorisation as in Spinou *et al*. [12]: |r|<0.4 (weak), 0.4<|r|<0.7 (moderate) and |r|>0.7 (strong). The statistical significance of r was evaluated at the 5% level of significance (α=0.05).

Meta-analysis was performed to determine the effect of bronchiectasis treatments *versus* placebo on HRQoL. The treatment, placebo and total combined effect sizes were classified according to the questionnaire. In addition, effect sizes were presented cumulatively by year, highlighting the direction of the effect size and whether there was stability through the time course.

Heterogeneity between studies was tested with the standard Chi-squared test. A random effects model was used to determine effect sizes with 95% confidence intervals. We tested for asymmetry of funnel plots to determine the presence of publication bias. Questionnaires with <15% floor/ceiling effects were defined as meeting standards and >15% floor/ceiling effects were defined as failed to have met standards.

Supplementary methodology is presented in appendix 3 in the supplementary material.

## Results

### Study characteristics

#### Study selection

**FIGURE 1:**
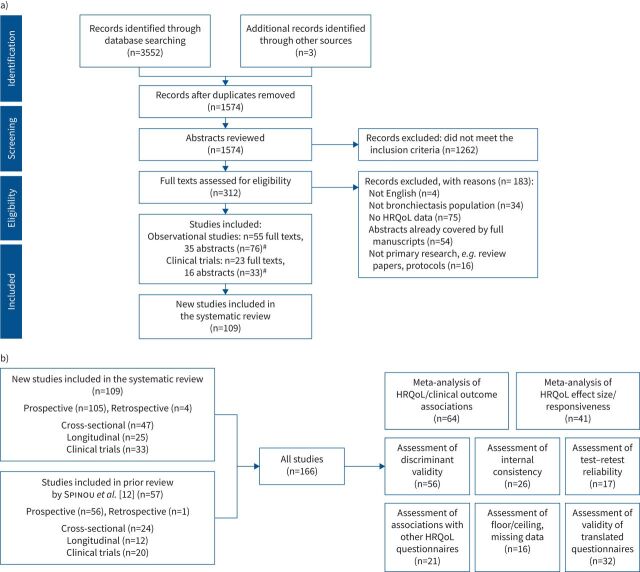
a) Flowchart of new studies (published between 6 November 2014 and 31 December 2020) included in the review based on the PRISMA (Preferred Reporting Items for Systematic Reviews and Meta-Analyses) protocols. HRQoL: health-related quality of life. ^#^: studies with multiple articles were subsequently combined and considered as a single study. The search yielded 1574 new studies. After screening the titles, abstracts and full texts when necessary, 109 new studies fulfilled the eligibility criteria for inclusion in the systematic review. b) Flowchart of total studies included in the review (studies reported by Spinou *et al*. [12] and studies published between 6 November 2014 and 31 December 2020).

The PRISMA flowchart shows the selection process of new studies, including reasons for study exclusion (figure 1).

#### Study overview and study quality

**FIGURE 2:**
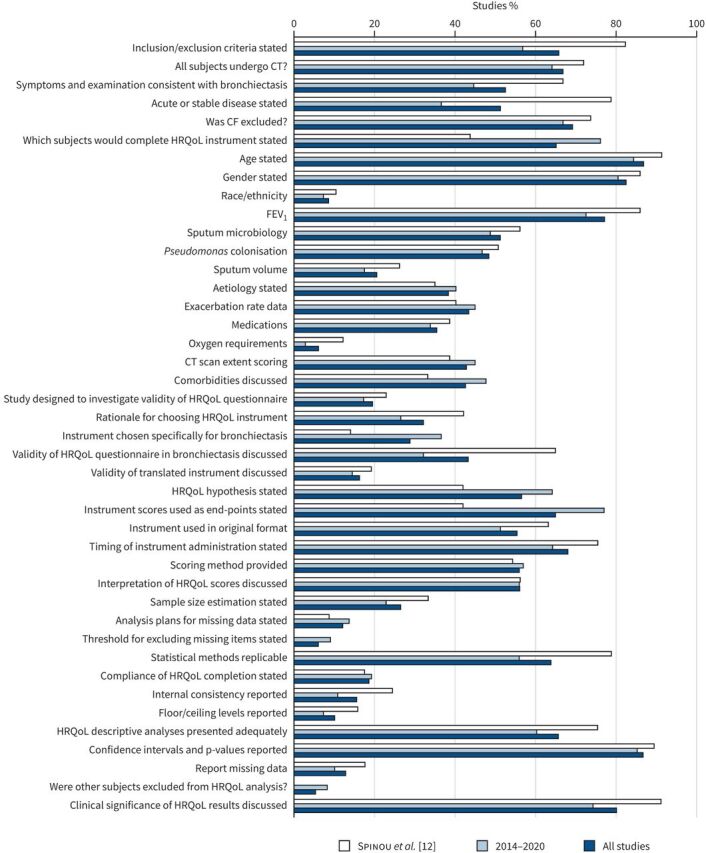
Quality of reporting of included studies. CT: computed tomography; CF: cystic fibrosis; FEV_1_: forced expiratory volume in 1 s; HRQoL: health-related quality of life; RCT: randomised controlled trial. *x*-axis: percentage of studies which meet each criterion; *y*-axis: quality criterion. Total studies, n=166: Spinou *et al*. [12], n=57 and 6 November 2014–31 December 2020, n=109. Spinou *et al*. [12] reported on quality of studies included in meta-analysis of HRQoL with clinical associations and did not report on quality of other studies (*i.e.* RCTs assessing responsiveness). Number of studies reported by Spinou *et al*. [12], n=37 (additionally we have assessed the quality of n=20 RCTs reported on in the prior review which were included in the current meta-analysis).

161 out of 166 studies included in the review were prospective. 71 studies reported cross-sectional findings and 37 studies reported longitudinal findings. 53 studies reported on clinical trials. Studies met a mean (range) of 45% (5–81%) of the quality criteria. The quality of the new studies published was similar to those presented in the prior review by Spinou *et al*. [12] (figure 2). The studies published in abstract form met fewer of the criteria for quality domains: mean (range) quality score 30% (5–57%). No study met the criteria for all quality domains. The full list of studies is included in the References list and supplementary material.

#### Patient characteristics

The studies included 47 540 patients with bronchiectasis. Mean (range) age was 60 (38–74) years and 62% (24–91%) were female. Eight studies recruited participants during an exacerbation [22–29], while all other studies recruited clinically stable patients. 115 studies required computed tomography scan findings to confirm the bronchiectasis diagnosis, while all other studies required a clinician diagnosis and symptoms consistent with bronchiectasis. Clinical characteristics of patients included in the newly identified studies are summarised in supplementary table E3.

### HRQoL questionnaires

#### Overview

The 12 HRQoL questionnaires used were categorised as bronchiectasis-specific, respiratory-specific or generic. Bronchiectasis-specific questionnaires were QOL-B and Bronchiectasis Health Questionnaire (BHQ). The respiratory-specific questionnaires were SGRQ, Leicester Cough Questionnaire (LCQ), Chronic Obstructive Pulmonary Disease (COPD) Assessment Test (CAT), Chronic Respiratory Disease Questionnaire (CRDQ), Sino-Nasal Outcome Test-20 (SNOT-20), Cough Quality of Life Questionnaire (CQLQ) and Seattle Obstructive Lung Disease Questionnaire (SOLQ). The generic questionnaires were the Medical Outcomes Study 36-item Short-Form Health Survey (SF-36), EuroQoL five-dimension five-level (EQ-5D-5L) and Nottingham Health Profile (NHP) (full descriptions in appendix 4 in the supplementary material). The number of studies that used the different HRQoL questionnaires is shown in supplementary figure E1. Overall, respiratory-specific questionnaires were the most widely used to assess HRQoL: SGRQ (58%) and LCQ (32%). The bronchiectasis-specific QOL-B was the third most commonly used (27%). Mean HRQoL scores, where available, are presented in supplementary table E4. Translated questionnaires are outlined in appendix 5 in the supplementary material.

#### Internal consistency

The internal consistency for all questionnaires is detailed in table 1. BHQ, LCQ, CAT and SF-36 had good internal consistency in all domains reported (Cronbach's α≥0.7) across all studies, and QOL-B, SGRQ, CRDQ and SOLQ had good internal consistency in all domains in the majority of (but not all) studies. Internal consistency was not reported for SNOT-20, CQLQ, EQ-5D-5L and NHP.

**TABLE 1:** Internal consistency and test–retest reliability of health-related quality of life (HRQoL) questionnaires

**Reference^#^**	**HRQoL questionnaire**	**Internal consistency**		**Test–retest reliability**		**Timeframe**
		**Domain**	**Cronbach's α** **^§^**	**Domain**	**ICC^§^**	
**Bronchiectasis-specific**						
Quittner (2010b)^¶^	QOL-B	8 domains	0.65–0.94	8 domains	0.72–0.88	2 weeks±3 days
Quittner (2010a)^¶^	QOL-B	8 domains	0.73–0.96	8 domains	NR	
Quittner (2014)^¶^	QOL-B V3.0^+^	Respiratory symptoms	0.82	Respiratory symptoms	0.80	2 weeks
		Physical functioning	0.94	Physical functioning	0.88	2 weeks
		Vitality	0.85	Vitality	0.67	2 weeks
		Role functioning	0.86	Role functioning	0.84	2 weeks
		Health perceptions	0.77	Health perceptions	0.78	2 weeks
		Emotional functioning	0.72	Emotional functioning	0.82	2 weeks
		Social functioning	0.66	Social functioning	0.85	2 weeks
		Treatment burden	0.84	Treatment burden	0.76	2 weeks
Quittner (2015)^¶^	QOL-B V3.0	Respiratory symptoms	0.81	Respiratory symptoms	0.83	2 weeks
		Physical functioning	0.91	Physical functioning	0.85	2 weeks
		Vitality	0.73	Vitality	0.74	2 weeks
		Role functioning	0.84	Role functioning	0.86	2 weeks
		Health perceptions	0.77	Health perceptions	0.76	2 weeks
		Emotional functioning	0.83	Emotional functioning	0.79	2 weeks
		Social functioning	0.77	Social functioning	0.80	2 weeks
		Treatment burden	0.78	Treatment burden	0.76	2 weeks
Olveira (2014a)^¶^	QOL-B V3.0	Respiratory symptoms	0.87	Respiratory symptoms	0.83	2 weeks
		Physical functioning	0.91	Physical functioning	0.88	2 weeks
		Vitality	0.82	Vitality	0.78	2 weeks
		Role functioning	0.84	Role functioning	0.86	2 weeks
		Health perceptions	0.71	Health perceptions	0.83	2 weeks
		Emotional functioning	0.84	Emotional functioning	0.86	2 weeks
		Social functioning	0.70	Social functioning	0.78	2 weeks
		Treatment burden	0.72	Treatment burden	0.68	2 weeks
Sokol (2019)	QOL-B (German)	8 domains	0.867–0.888	8 domains	NR	
Speck (2018)	QOL-B	Respiratory symptoms	0.81	Respiratory symptoms	0.70	
		Physical functioning	NR	Physical functioning	NR	
		Vitality	NR	Vitality	NR	
		Role functioning	NR	Role functioning	NR	
		Health perceptions	NR	Health perceptions	NR	
		Emotional functioning	NR	Emotional functioning	NR	
		Social functioning	NR	Social functioning	NR	
		Treatment burden	NR	Treatment burden	NR	
Spinou (2018)	QOL-B	8 domains	0.46–0.90	8 domains	NR	
Liu (2019)	QOL-B	8 domains	>0.64	8 domains	NR	
De Camargo (2020)	QOL-B	Respiratory symptoms	0.85	Respiratory symptoms	0.85	1–2 weeks
		Physical functioning	0.91	Physical functioning	0.91	1–2 weeks
		Vitality	0.58	Vitality	0.58	1–2 weeks
		Role functioning	0.70	Role functioning	0.70	1–2 weeks
		Health perceptions	0.77	Health perceptions	0.77	1–2 weeks
		Emotional functioning	0.91	Emotional functioning	0.91	1–2 weeks
		Social functioning	0.93	Social functioning	0.93	1–2 weeks
		Treatment burden	0.70	Treatment burden	0.70	1–2 weeks
Spinou (2017b)	BHQ	Total	0.85	Total	0.89	2 weeks
Spinou (2018)	BHQ	Total	0.84	Total	NR	
Gissel (2020)	BHQ	Total	0.739	Total	NR	
**Respiratory-specific**						
Wilson (1997a)^¶^	SGRQ	Total	NR	Total	0.97	2 weeks
		Symptoms	0.90	Symptoms	0.93	2 weeks
		Activity	0.89	Activity	0.98	2 weeks
		Impact	0.92	Impact	0.94	2 weeks
Chan (2002)^¶^	SGRQ	Total	0.92	Total	0.93	2 weeks
		Symptoms	0.59	Symptoms	0.94	2 weeks
		Activity	0.91	Activity	0.84	2 weeks
		Impact	0.88	Impact	0.89	2 weeks
Martinez-Garcia (2005)^¶^	SGRQ	Total	0.90	Total	NR	
		Symptoms	0.81	Symptoms	NR	
		Activity	0.87	Activity	NR	
		Impact	0.81	Impact	NR	
Speck (2018)	SGRQ	Total	NR	Total	NR	
		Symptoms	0.646	Symptoms	NR	
		Activity	NR	Activity	NR	
		Impact	NR	Impact	NR	
Murray (2009b)^¶^	LCQ	Total	NR	Total	0.96	6 months
		Physical	NR	Physical	NR	
		Psychological	NR	Psychological	NR	
		Social	NR	Social	NR	
Munoz (2013)^¶^	LCQ	Total	0.91	Total	NR	
		Physical	0.94	Physical	NR	
		Psychological	0.93	Psychological	NR	
		Social	0.93	Social	NR	
Gao (2014b)	LCQ (Mandarin)	Total	0.93	Total	0.89	6 months
		Physical	0.83	Physical	0.84	6 months
		Psychological	0.88	Psychological	0.82	6 months
		Social	0.82	Social	0.89	6 months
Munoz (2016)	LCQ (Spanish)	Total	0.91	Total	0.84	15 days
		Physical	0.87	Physical	0.87	15 days
		Psychological	0.87	Psychological	0.82	15 days
		Social	0.86	Social	0.79	15 days
Lee (2012)^¶^	CAT	Total	0.84	Total	NR	
Lanza (2018)	CAT	Total	0.91	Total	0.84	7–10 days
De la Rosa Carrillo (2020)	CAT	Total	0.86	Total	0.95	15 days
Finch (2020)	CAT	Total	NR	Total	0.88	4 weeks
Vodanovich (2015)	CRDQ	Total	NR	Total	0.82	9 weeks
		Dyspnoea	0.76	Dyspnoea	0.85	9 weeks
		Fatigue	0.85	Fatigue	0.69	9 weeks
		Emotional functioning	0.94	Emotional functioning	0.83	9 weeks
		Mastery	0.80	Mastery	0.77	9 weeks
Bulcun (2015)	SOLQ (Turkish)	Physical functioning	0.72	Physical functioning	0.83	2 weeks
		Emotional functioning	0.91	Emotional functioning	0.71	2 weeks
		Coping skills	0.74	Coping skills	0.81	2 weeks
		Treatment satisfaction	0.62	Treatment satisfaction	0.65	2 weeks
**Generic**						
Guilemany (2006)^¶^	SF-36	8 domains	0.75–0.91	8 domains	NR	

ICC: intraclass correlation coefficient; QOL-B: Quality of Life-Bronchiectasis; BHQ: Bronchiectasis Health Questionnaire; SGRQ: St George's Respiratory Questionnaire; LCQ: Leicester Cough Questionnaire; CAT: Chronic Obstructive Pulmonary Disease (COPD) Assessment Test; CRDQ: Chronic Respiratory Disease Questionnaire; SOLQ: Seattle Obstructive Lung Disease Questionnaire; SF-36: Medical Outcomes Study 36-item Short-Form Health Survey; NR: not reported. ^#^: see the References list and supplementary material; ^¶^: studies reported in the prior review by Spinou *et al*. [12]; ^+^: the repeatability of QOL-B V3.0 was not reported (table presents data from QOL-B V2.0); ^§^: Cronbach's α and ICC ≥0.7 are considered acceptable for HRQoL questionnaires.

#### Test–retest reliability

Test–retest reliability for all questionnaires is detailed in table 1. BHQ, SGRQ, LCQ and CAT had good test–retest reliability in all domains reported (ICC ≥0.7) across all studies, and QOL-B, CRDQ and SOLQ had good test–retest reliability in all domains in the majority of (but not all) studies. The majority of studies reported test–retest reliability over 2 weeks; however, only a few studies reported test–retest reliability over a longer period of time (up to 6 months). Test–retest reliability was not reported for SNOT-20, CQLQ, SF-36, NHP and EQ-5D-5L.

#### Discriminant validity

Discriminant validity for all questionnaires is detailed in supplementary table E5 and appendix 6 in the supplementary material. Disease-specific and respiratory-specific HRQoL questionnaires were most commonly able to discriminate patients based on demographics, disease severity, exacerbations and bacteriology, as well as a range of other symptoms. In terms of disease-specific questionnaires, QOL-B had the most data available and it was able to discriminate patients based on demographics, disease severity, exacerbation rate, sputum and bacteriology, signs and symptoms, adherence to treatment, and exercise capacity. BHQ had some data available and it was able to discriminate patients based on demographics, lung function, exacerbation rate and hospital admissions. In terms of respiratory-specific questionnaires, SGRQ had the most data available and it was able to discriminate based on demographics, disease severity, exacerbation rate, bacteriology, signs and symptoms, and exercise capacity. LCQ, CAT, CRDQ, SNOT-20 and SOLQ had some data available. Discriminant data were limited for generic HRQoL questionnaires. Discriminant validity was not reported for CQLQ and NHP.

#### Associations between HRQoL and clinical measures

The associations between HRQoL and clinical measures were evaluated in a meta-analysis. The results are summarised in table 2 and forest plots with subgroup analysis of each HRQoL questionnaire are presented in supplementary figures E2–E9 and indicate a high level of heterogeneity. The strongest associations, albeit moderate, were between HRQoL and cough (r=0.5, I^2^=66%), dyspnoea (r=0.5, I^2^=87%) and *Pseudomonas aeruginosa* colonisation (r=0.5, I^2^=99%). Moderate associations existed between HRQoL and wheeze (r=0.4, I^2^=0%), fatigue (r=0.4, I^2^=36%), exercise capacity (r= −0.4, I^2^=73%), anxiety (r=0.4, I^2^=59%), depression (r=0.5, I^2^=72%) and sputum volume (r=0.4, I^2^=89%). Weak to moderate associations existed between HRQoL questionnaires and lung function (r= −0.3, I^2^=51–71%), bacteriology (r=0.2, I^2^=44%), inflammatory markers (r=0.3, I^2^=13%), healthcare utilisation (r=0.3–0.4, I^2^=8–66%), disease severity (r=0.2–0.4, I^2^=0–97%), demographics (r= −0.2–0.2, I^2^=0–65%), oxygen saturation (r= −0.3, I^2^=0%) and comorbidities (r=0.08, I^2^=0%).

**TABLE 2:** Correlations between health-related quality of life (HRQoL) questionnaires and clinical measures

**Clinical measures**	**Studies n**	**Overall participants n**	**Correlation (95% CI)**	**p-value**	**I^2^-value %**
**Symptoms**					
Cough	7	577	0.524 (0.404, 0.626)	<0.01	66
Dyspnoea	119	25 953	0.491 (0.425, 0.551)	<0.01	87
Wheeze	2	213	0.422 (0.304, 0.527)	<0.01	0
Fatigue	4	182	0.424 (0.231, 0.585)	<0.01	36
Anxiety (including HADS)	114	11 289	0.430 (0.352, 0.502)	<0.01	59
Depression (including HADS)	114	11 295	0.455 (0.362, 0.538)	<0.01	72
**Lung function/exercise capacity**					
FVC % pred	112	11 656	−0.291 (−0.360, −0.218)	<0.01	51
FEV_1_ % pred	235	7032	−0.309 (−0.355, −0.260)	<0.01	52
Exercise capacity	223	12 079	−0.388 (−0.464, −0.305)	<0.01	73
**Sputum bacteriology**					
Bacteriology	5	1387	0.207 (0.129, 0.281)	<0.01	44
*Pseudomonas aeruginosa* colonisation	45	11 713	0.497 (0.017, 0.791)	<0.01	99
Inflammatory markers	112	11 808	0.287 (0.238, 0.334)	<0.01	13
**Healthcare utilisation**					
Infection/exacerbation rate	120	36 367	0.324 (0.277, 0.369)	<0.01	66
Hospital admissions rate	45	12 027	0.366 (0.326, 0.406)	<0.01	15
**Disease severity**					
BSI	120	26 557	0.393 (0.351, 0.433)	<0.01	64
FACED	510	11 975	0.311 (0.271, 0.351)	<0.01	0
CT bronchiectasis score	10	1880	0.341 (0.052, 0.578)	0.02	97
CT lung zone	2	142	0.233 (0.069, 0.385)	<0.01	0
**Demographics**					
Age	9	1497	0.167 (0.109, 0.225)	<0.01	16
Sex	6	868	−0.200 (−0.353, −0.037)	0.02	65
BMI	58	11 413	−0.161 (−0.211, −0.109)	<0.01	0
**Other**					
Sputum volume	69	14 971	0.359 (0.275, 0.437)	<0.01	89
Oxygen saturation	4	324	−0.345 (−0.439, −0.244)	<0.01	0
Comorbidities	2	815	0.085 (0.016, 0.153)	0.02	0

For the purpose of comparison, higher score indicates poorer HRQoL. I^2^=0% indicates no observed heterogeneity, while I^2^>50% indicates substantial heterogeneity. HADS: Hospital Anxiety and Depression; FVC: forced vital capacity; FEV_1_: forced expiratory volume in 1 s; BSI: Bronchiectasis Severity Index; FACED: FEV_1_, age, chronic colonisation, extension, dyspnoea; CT: computed tomography; BMI: body mass index.

#### Associations between HRQoL questionnaires

Multiple questionnaires were used in 34% of studies and 21 studies directly assessed associations between the questionnaires (supplementary table E6). There was some exploration of correlations between the different questionnaires. The data available do not permit comprehensive comparisons; however, there were data suggesting strong correlations existed between bronchiectasis-specific and respiratory-specific questionnaires, but not between bronchiectasis-specific and generic questionnaires.

#### Responsiveness

**FIGURE 3:**
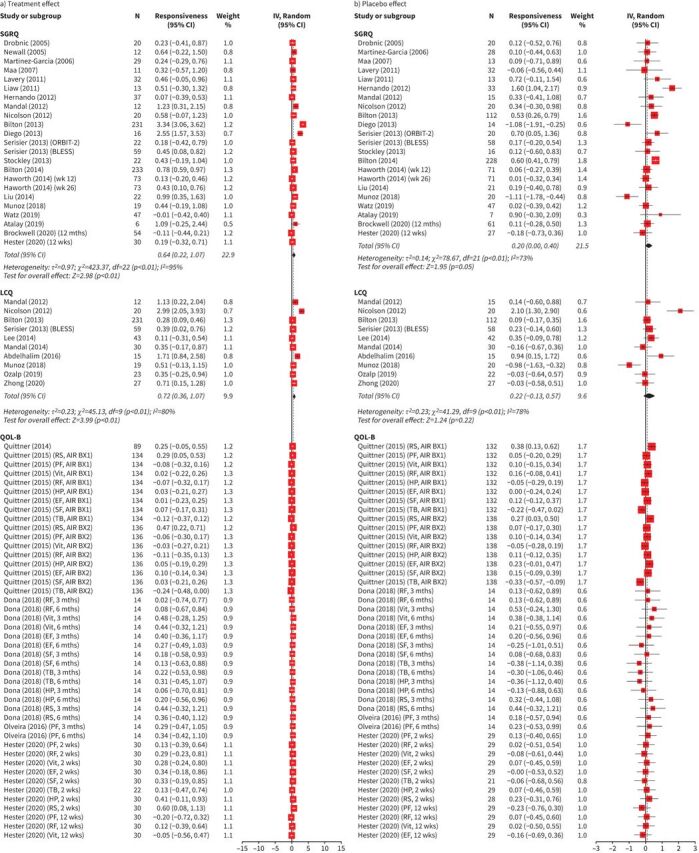

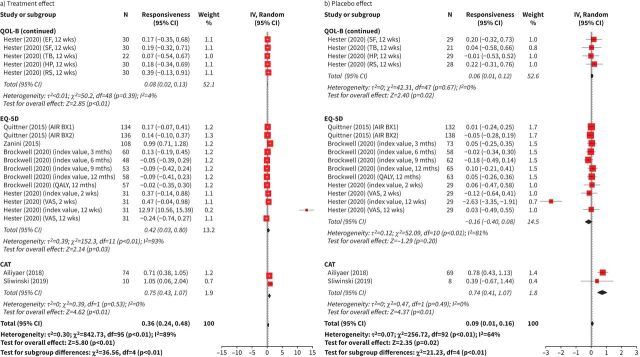
Forest plots for responsiveness of health-related quality of life (HRQoL) for a) treatment effect and b) placebo effect classified according to the HRQoL questionnaire: St George's Respiratory Questionnaire (SGRQ), Leicester Cough Questionnaire (LCQ), Quality of Life-Bronchiectasis (QOL-B), EuroQoL five-dimension (EQ-5D) and Chronic Obstructive Pulmonary Disease (COPD) Assessment Test (CAT). Total number of studies included in meta-analysis, n=41. The full list of studies is included in the References list and supplementary material. For HRQoL questionnaires where a lower score equates to better/improved HRQoL, scores were converted for meta-analysis. For all included questionnaires, positive effect sizes indicate improvement in HRQoL, while negative effect sizes indicate worsening of HRQoL. Higher score=better HRQoL. wk: week; mth: month; IV: interval variable; Random: random effect; RS: respiratory symptoms; PF: physical functioning; Vit: vitality; RF: role functioning; HP: health perceptions; EF: emotional functioning; SF: social functioning; TB: treatment burden; QALY: quality-adjusted life-years; VAS: visual analogue scale.

**FIGURE 4:**
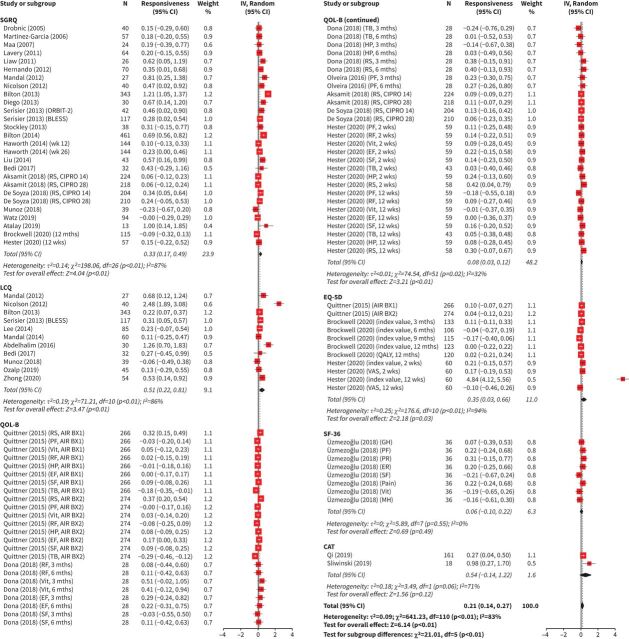
Forest plots for responsiveness of health-related quality of life (HRQoL) for total combined effect classified according to the HRQoL questionnaire: St George's Respiratory Questionnaire (SGRQ), Leicester Cough Questionnaire (LCQ), Quality of Life-Bronchiectasis (QOL-B), EuroQoL five-dimension (EQ-5D), Medical Outcomes Study 36-item Short-Form Health Survey (SF-36) and Chronic Obstructive Pulmonary Disease (COPD) Assessment Test (CAT). Total number of studies included in meta-analysis, n=41. The full list of studies is included in the References list and supplementary material. For HRQoL questionnaires where a lower score equates to better/improved HRQoL, scores were converted for meta-analysis. For all included questionnaires, positive effect sizes indicate improvement in HRQoL, while negative effect sizes indicate worsening of HRQoL. Higher score=better HRQoL. wk: week; mth: month; IV: interval variable; Random: random effect; RS: respiratory symptoms; PF: physical functioning; Vit: vitality; RF: role functioning; HP: health perceptions; EF: emotional functioning; SF: social functioning; TB: treatment burden; QALY: quality-adjusted life-years; VAS: visual analogue scale; GH: general health; PR: physical role; ER: emotional role; Pain: bodily pain; MH: mental health.

The responsiveness analysis included 4153 patients. Interventions included mucoactives, long-term inhaled or oral antibiotics, inhaled corticosteroids, statin treatment, neutrophil elastase inhibitors, physiotherapy, exercise, nutritional supplements, self-management programmes and alternative medicines. The treatment and placebo effect estimates for SGRQ, LCQ, QOL-B, EQ-5D-5L and CAT are presented in figure 3a and b. The total combined effect estimates for SGRQ, LCQ, QOL-B, EQ-5D-5L, SF-36 and CAT are presented in figure 4. The I^2^-values for the treatment, placebo and total combined effect sizes were equal to 89%, 64% and 83%, respectively, indicating a high level of heterogeneity (all p<0.01). There was evidence that the observed symmetry in the funnel plots was marginally significant at the 5% significance level for the treatment effect, statistically significant for the placebo effect and nonsignificant for the total combined effect (supplementary figures E10 and E11). The effect estimate for treatment was 0.36 (95% CI 0.24, 0.48). The treatment effect for the disease-specific QOL-B (all domains) was small (0.08 (95% CI 0.02, 0.13)). The treatment effects were highest for the respiratory-specific questionnaires SGRQ (0.64 (95% CI 0.22, 1.07)), LCQ (0.72 (95% CI 0.36, 1.07)) and CAT (0.75 (95% CI 0.43, 1.07)). The treatment effect was significant for the generic questionnaire EQ-5D-5L (0.42 (95% CI 0.03, 0.80)). The effect estimate for placebo was statistically significant, but the effect was weak (0.09 (95% CI 0.01, 0.16)). The total combined effect size was 0.21 (95% CI 0.14, 0.27). The treatment, placebo and total combined effect estimates presented cumulatively by year are shown in supplementary figure E12. The HRQoL questionnaires became less responsive over the years. Responsiveness has not been investigated for BHQ, CRDQ, CQLQ, SOLQ and NHP.

#### Floor/ceiling effects and missing data

Floor/ceiling effects were reported for QOL-B, BHQ, SGRQ, LCQ, CAT and CRDQ (appendix 7 in the supplementary material). All studies met the standards for floor effects (<15% participants) for all questionnaires. For QOL-B, four out of five studies failed to meet the standards for ceiling effects (>15% participants). For LCQ, one out of two studies failed to meet the standards for ceiling effects (>15%). All other questionnaires met the standards for ceiling effects (<15% participants). Missing data were reported for QOL-B, BHQ, SGRQ and CAT.

#### Minimal clinically important difference

MCID for QOL-B was determined from a bronchiectasis population. MCID for CAT was determined from a bronchiectasis population and was similar to MCIDs reported in COPD. MCIDs for SGRQ, LCQ, CRDQ, CQLQ, SF-36 and EQ-5D-5L visual analogue scale (VAS) component were based on other disease populations and are commonly used in bronchiectasis clinical trials. MCIDs for BHQ, SOLQ, CQLQ, SNOT-20, EQ-5D-5L five-digit code/index value and NHP have not been investigated in bronchiectasis or other respiratory conditions (table 3).

**TABLE 3:** The origin (studies and population) of the minimal clinically important difference (MCID) of health-related quality of life (HRQoL) questionnaires

**HRQoL questionnaire**	**MCID study disease population**	**MCID units**	**Studies referenced as origin of MCID^#^**
**QOL-B**	Bronchiectasis	6.8 (respiratory symptoms)	Olveira (2014a)
	Bronchiectasis	7.0–10.0	Quittner (2015)
	Bronchiectasis	8.6	De Camargo (2020)
	Bronchiectasis	8.1–8.3 (respiratory symptoms)	Tong (2020)
**SGRQ**	COPD	4.0	Jones (2005)
	COPD	5.8	Schünemann (2003)
	IPF	7.0	Swigris (2005)
**LCQ**	Chronic cough	1.3	Raj (2009)
**CQLQ**	IPF	5.0–5.7	Lechtzin (2013)
	Chronic cough	10.6	Fletcher (2010)
**CAT**	COPD	1.2–3.8	Kon (2013)
	COPD	3.5	Zhou (2018)
	Bronchiectasis	3.0	De la Rosa Carrillo (2020)
	Bronchiectasis	3.0–4.0	Finch (2020)
**CRDQ**	COPD	0.5	Jaeschke (1989)
**SF-36**	IPF	2.0–4.0	Swigris (2005)
**EQ-5D VAS**	COPD	8.0	Zanini (2015b)

QOL-B: Quality of Life-Bronchiectasis; SGRQ: St George's Respiratory Questionnaire; COPD: chronic obstructive pulmonary disease; IPF: idiopathic pulmonary fibrosis; LCQ: Leicester Cough Questionnaire; CQLQ: Cough Quality of Life Questionnaire; CAT: COPD Assessment Test; CRDQ: Chronic Respiratory Disease Questionnaire; SF-36: Medical Outcomes Study 36-item Short-Form Health Survey; EQ-5D: EuroQoL five-dimension; VAS: visual analogue scale. ^#^: see the References list and supplementary material.

## Discussion

This systematic review with new synthesised data from 166 studies highlights the value in evaluating psychometric properties of HRQoL to inform the choice of questionnaires used in clinical trials.

The psychometric properties varied between questionnaires. Internal consistency and test–retest reliability were generally good for the majority of questionnaires. However, the timeframe explored for test–retest was found to be generally quite short (≤15 days), except for LCQ (6 months) [30, 31], CAT (4 weeks) [32] and CRDQ (9 weeks) [33]. Given that the majority of randomised controlled trials are longer than 6 months, this is important to consider when selecting a HRQoL questionnaire. In ORBIT-3 and -4, QOL-B respiratory symptoms scores were not significantly different between baseline and 48 weeks in the treatment group [34]; however, they were significantly improved during on-treatment periods and these correlated with changes in bacterial load [3]. Future studies should investigate test–retest reliability of the HRQoL questionnaires over a longer timeframe in clinically stable patients and at multiple time-points which accurately reflect long-term variation in symptoms.

The HRQoL questionnaires were generally able to discriminate between demographics, important markers of clinical status and disease severity, as well as symptoms, highlighting that most questionnaires capture the impact of bronchiectasis on HRQoL.

The majority of HRQoL questionnaires were responsive, and the meta-analysis showed a difference between the treatment and placebo effect across the questionnaires. The effect sizes, categorised by HRQoL questionnaire, were higher in the respiratory-specific questionnaires (SGRQ, LCQ and CAT) compared with the bronchiectasis-specific QOL-B and this should be taken into consideration when selecting a HRQoL questionnaire for clinical trials. The lowest effect size was in the generic questionnaire SF-36.

Most recent studies used the QOL-B respiratory symptoms domain, which contains nine questions related to potentially important symptoms experienced by bronchiectasis patients as primary or secondary end-points. Crichton *et al*. [35] found that the QOL-B respiratory symptoms domain was unresponsive to inhaled antibiotic treatment, despite improvements in cough and sputum production. Other large randomised trials reported similar findings [13, 36]. These studies highlight that responsiveness may depend largely on the type of intervention and its ability to affect symptoms in specific domains of HRQoL questionnaires.

The reasons for an apparent decrease in the responsiveness of HRQoL questionnaires over time may be attributed to improved bronchiectasis management in recent years resulting in subtle, nonsignificant changes in outcome measures, such as HRQoL [37]. Establishment of the MCID is essential for clinical interpretation of HRQoL scores; however, only two questionnaires (QOL-B and CAT) have MCID values for bronchiectasis populations. Future studies need to address the MCID for the most commonly used HRQoL questionnaires, especially since large randomised trials for promising bronchiectasis treatments failed to demonstrate a significant change in HRQoL with QOL-B respiratory symptoms [13, 36], SGRQ [9, 13, 38, 39] and LCQ [39] despite evidence there were other clinically meaningful changes.

The findings for floor/ceiling effects are important for studies exploring interventions in early disease where HRQoL impairments may be less obvious and should be taken into consideration in such future studies.

Some studies used multiple questionnaires and this has resulted in challenges in interpretation, *e.g.* in RESPIRE-1 [13], and additional burden for patients. Multiple questionnaires may be valuable when they capture different aspects of HRQoL.

Our review has limitations. We did not contact study authors for unpublished data and we chose to limit the review to articles published in the English language only. Our methodology attempted to include bronchiectasis studies with homogenous populations. We recognise that 31% of studies included in our review did not require a radiological confirmation of bronchiectasis; however, they required a clinician diagnosis and symptoms consistent with bronchiectasis. The presence of heterogeneity in our meta-analysis results was therefore expected given the differences in the methodology, study population, sample size and study quality. Our methodology assumes that the interventions used in clinical trials are effective compared with placebo and also that the specific domain captured the effect of the intervention. Ideally, a positive control is needed to assess responsiveness, *e.g.* an acute exacerbation of bronchiectasis known to affect all domains of HRQoL questionnaires. This has been explored in a small number of studies only [30, 40–42] and this will be an important focus in future clinical trials. A limitation of HRQoL questionnaires is that they generate aggregate scores to represent distinct symptoms, *e.g.* cough, dyspnoea and chest pain, and not all may improve with a therapy. A recent study by Crichton *et al*. [35] illustrated this by showing that with QOL-B, inhaled antibiotics showed marked improvements in cough, sputum production and sputum colour, but there were no changes in any other symptoms.

This review considers the psychometric properties; however, the patient perspective is also key in the selection of HRQoL questionnaires. In cystic fibrosis, recent studies have involved patients and their caregivers in the assessment and improvement of PROs [43]. In bronchiectasis, Dudgeon *et al*. [44] explored the patient perspective of four HRQoL questionnaires (QOL-B, SGRQ, LCQ and CAT). The authors concluded that bronchiectasis symptoms are highly individual and HRQoL tools do not fully capture the burden of disease. This is beginning to be explored by Crichton *et al*. [45] through the development of a novel PRO measure, *i.e.* the Bronchiectasis Impact Measure (BIM). Future studies of HRQoL tools in bronchiectasis should involve patients and their caregivers.

### Recommendations for future research

Future studies should focus specifically on the medium- to long-term test–retest reliability, responsiveness and MCID in HRQoL questionnaires which show the best potential in bronchiectasis, such as QOL-B, BHQ and SGRQ. Future studies should focus on the involvement of patients and their caregivers in the assessment and improvement of these HRQoL questionnaires.

### Conclusions

The consideration of psychometrics properties of HRQoL questionnaires is an important component of decision making to ensure optimal choice of HRQoL questionnaires in clinical trials. SGRQ was the most widely used HRQoL questionnaire in bronchiectasis studies and it had good psychometric properties; however, good psychometric data are emerging on bronchiectasis-specific HRQoL questionnaires such as QOL-B and BHQ.

## Supplementary material

10.1183/13993003.00025-2021.Supp1 **Please note:** supplementary material is not edited by the Editorial Office, and is uploaded as it has been supplied by the author.
Supplementary material ERJ-00025-2021.SUPPLEMENT

## Shareable PDF

10.1183/13993003.00025-2021.Shareable1 This one-page PDF can be shared freely online.
Shareable PDF ERJ-00025-2021.Shareable
