# Supplementary material for: Psychometrics of health-related quality of life questionnaires in bronchiectasis: a systematic review and meta-analysis
Source: Eur Respir J. 2021 Nov 11;58(5):2100025. doi: 10.1183/13993003.00025-2021 (PMC8581652; doi:10.1183/13993003.00025-2021)
Supplement: Supplementary file 1 [file ERJ-00025-2021.SUPPLEMENT.pdf]

ONLINE SUPPLEMENT FOR

Psychometrics of HRQoL questionnaires in bronchiectasis: A systematic review and meta-analysis

Rebecca H. McLeese,[1] Arietta Spinou,[2] Zina Alfahl,[1,3] Michail Tsagris,[4] J. Stuart Elborn,[5] James D. Chalmers,[6] Anthony De Soyza,[7] Michael R. Loebinger,[8] Surinder S. Birring,[9] Konstantinos C. Fragkos,[10] Robert Wilson,[8] Katherine O'Neill,[1,5,11] Judy M. Bradley,[1,5,11]

[1]The Wellcome Trust-Wolfson Northern Ireland Clinical Research Facility, School of Medicine, Dentistry and Biomedical Sciences, Queen's University Belfast, Belfast, UK.

[2]Population Health Sciences, Faculty of Life Sciences and Medicine, King's College London, London, UK

[3]School of Pharmacy, Queen's University Belfast, Belfast, UK

[4]Department of Economics, University of Crete, 74100, Rethymnon, Greece

[5]Wellcome-Wolfson Institute for Experimental Medicine, School of Medicine, Dentistry and Biomedical Sciences, Queen's University Belfast, Belfast, UK

[6]Scottish Centre for Respiratory Research, University of Dundee, Dundee, UK

[7]Institute of Cellular Medicine, Newcastle University and Freeman Hospital, Sir William Leech Research Centre, Respiratory Department, Newcastle upon Tyne, UK

[8]Host Defence Unit, Royal Brompton Hospital, London, UK

[9]Centre for Human & Applied Physiological Sciences, School of Basic & Medical Biosciences, Faculty of Life Sciences & Medicine, King's College London, UK

[10]Division of Medicine, University College London (UCL), London, UK

[11]These two authors are co-senior authors

Corresponding author: Professor Judy M. Bradley, Queen's University Belfast, The Wellcome Trust-Wolfson Northern Ireland Clinical Research Facility, Belfast, BT9 7AB. Judy.Bradley@qub.ac.uk

Appendix 1 Quality criteria used to assess articles included in the review.

Appendix 2 Data searches, study selection and data extraction: Author roles.

Appendix 3 Supplementary Methodology.

Appendix 4 Description of health-related quality of life questionnaires.

Appendix 5 The validation of translated health-related quality of life questionnaires.

Appendix 6 Discriminant validity of health-related quality of life questionnaires.

Appendix 7 Floor/ceiling effects and missing data.

Table E1 Inclusion and Exclusion Criteria and Psychometric Properties Assessed.

Table E2 Search terms and number of studies retrieved in each database.

Table E3 Characteristics of new studies included in the literature review.

Table E4 Average health-related quality of life scores for questionnaires.

Table E5 Studies reporting discriminant validity ($p<0.05$) of health-related quality of life questionnaires.

Table E6 Associations between health-related quality of life questionnaires.

Figure E1 Health-related quality of life questionnaires utilized in studies reported by Spinou et al. and studies published between 06 November 2014 and 31 December 2020. X axis = Percentage of total studies (total studies=166; Spinou et al. 2016, n=57; 2014-31 December 2020, n=109), Y axis = questionnaire.

Figure E2 Forest plot for the correlation between health-related quality of life and **(a)** cough, **(b)** dyspnoea, **(c)** wheeze and **(d)** fatigue. High score= poor health-related quality of life.

Figure E3 Forest plot for the correlation between health-related quality of life and **(a)** anxiety (including HADS) and **(b)** depression (including HADS). High score= poor health-related quality of life.

Figure E4 Forest plot for the correlation between health-related quality of life and **(a)** FVC (%) predicted, **(b)** FEV_1 (%) predicted and **(c)** exercise capacity. High score= poor health-related quality of life.

Figure E5 Forest plot for the correlation between health-related quality of life and **(a)** bacteriology, **(b)** *Pseudomonas aeruginosa* (Pa) colonization and **(c)** inflammatory markers. High score= poor health-related quality of life.

Figure E6 Forest plot for the correlation between health-related quality of life and **(a)** infection/exacerbation rate and **(b)** hospital admissions rate. High score= poor health-

related quality of life.

Figure E7 Forest plot for the correlation between health-related quality of life and **(a)** Bronchiectasis Severity Index (BSI), **(b)** FACED score**, (c)** CT bronchiectasis score and **(d)** CT lung zone. High score= poor health-related quality of life.

Figure E8 Forest plot for the correlation between health-related quality of life and **(a)** age, **(b)** sex **and (c)** BMI. High score= poor health-related quality of life.

Figure E9 Forest plot for the correlation between health-related quality of life and **(a)** sputum volume, **(b)** oxygen saturation **and (c)** co-morbidities. High score= poor health-related quality of life.

Figures E10 Funnel plot of responsiveness vs. standard error for health-related quality of life (HRQoL) for **(a)** Treatment effect, **(b)** Placebo effect and **(c)** Total combined effect assessing the publication bias for the meta-analysis. Each study is illustrated as a dot.

Figures E11 Funnel plot of responsiveness vs. sample size for health-related quality of life (HRQoL) for **(a)** Treatment effect, **(b)** Placebo effect and **(c)** Total combined effect assessing the publication bias for the meta-analysis. Each study is illustrated as a dot.

Figures E12 Forest plots for responsiveness of health-related quality of life (HRQoL) for **(a)** Treatment effect, **(b)** Placebo effect and **(c)** Total combined effect presented cumulatively by year. The HRQoL questionnaires become less responsive over the years as shown by decreasing effect sizes. Total number of studies included in meta-analysis=41. Higher score = better HRQoL.

References References of supplementary material and additional references of the main paper.

Online Appendix 1 Quality criteria used to assess articles included in the review (Swigris et al., 2005, Spinou et al., 2016)

Title:
Authors:

A. Bronchiectasis Case Definition and HRQoL Study Subject Assembly

	Yes	No
1) Were the inclusion and exclusion criteria reported?		
2) Did all bronchiectasis subjects undergo CT or bronchography?		
3) Inclusion criteria included symptoms and physical examination consistent with a diagnosis of bronchiectasis?		
4) Did the authors state if acute or stable disease and definition of it?		
5) Was cystic fibrosis excluded for bronchiectasis?		
6) Did the authors state which subjects would complete the HRQoL instrument?		

B. Clinical Characteristics of Bronchiectasis Subjects

	Yes	No
7) For the subjects with bronchiectasis, is the age distribution given?		
8) For the subjects with bronchiectasis, is the gender distribution given?		
9) For the subjects with bronchiectasis, is the race/ethnicity distribution given?		
10) For the subjects with bronchiectasis, are there data for FEV1?		
11) For the subjects with bronchiectasis, are there data for sputum microbiology?		
12) For the subjects with bronchiectasis, are there data for pseudomonas chronic colonisation or intermittent infection?		
13) For the subjects with bronchiectasis, are there data for sputum volume?		
14) For the subjects with bronchiectasis, are there data for aetiology?		
15) For the subjects with bronchiectasis, are there data for exacerbation rate?		
16) For the subjects with bronchiectasis, are there data for medications?		
17) For the subjects with bronchiectasis, are there data for oxygen requirements?		
18) For the subjects with bronchiectasis, is there an objective measure (e.g. a CT scoring scale) for the degree of abnormality on CT?		
19) Were potentially relevant comorbidities discussed?		

C. HRQoL Instrument Selection

	Yes	No
20) Was this study designed to evaluate, validate or develop a HRQoL questionnaire in bronchiectasis?		
21) If this study is not designed to "validate" an instrument, did the authors provide a rationale for choosing the HRQoL instrument(s) studied?		
22) Was the instrument(s) chosen for this study specifically designed to assess HRQoL in bronchiectasis patients?		

23) If this study is not a study designed to “validate” an instrument, did the authors discuss (or reference) previously published data that supports the reliability (e.g. test-retest and internal consistency) of the chosen instrument(s) in bronchiectasis patients?		
24) If a translated instrument was used, did the authors discuss (or reference) data that verifies the cultural validity of the translated instrument?		

D. HRQoL Endpoints and Instrument Administration

	Yes	No
25) Was the hypothesis regarding HRQoL stated?		
26) Did the authors state which instrument scores (e.g. the total instrument score or specific domain scores) were specified as endpoints?		
27) Was the instrument(s) administered in the format (e.g. self- or interviewer-administered) that the instrument developers intended?		
28) Did the authors adequately describe the timing of instrument(s) administration (as applicable) in the context of a single administration, an individual study visit, and throughout the course of the study?		
29) Did the authors provide details of the scoring methods used?		
30) Did the authors provide information on how to interpret scores (e.g. do higher scores indicate better or worse HRQoL)?		

E. Methods of Statistical Analysis

	Yes	No
31) Did the authors provide documentation of sample size estimation?		
32) Did the authors describe how missing data (e.g. items missing responses and data from drop-outs) would be accounted for (e.g. by using imputation methods)?		
33) Did the authors define what would deem a subject’s HRQoL data inadequate (or did they define what constitutes adequate data) for analysis?		
34) Were the statistical methods used to assess (and if applicable, to compare) HRQoL described in enough detail that other researchers could repeat the analysis if the full data were made available?		

F. Reporting Results

	Yes	No
35) Was compliance (% of patients who were asked to complete the instrument and actually completed it) data for each administration given?		
36) Did the investigators calculate Internal Consistency Reliability (i.e. Cronbach’s alpha) for the instrument (and/or its subscales in this study’s population?		
37) Were the floor and ceiling effect levels reported?		
38) Were the results of the primary and secondary HRQoL analyses presented adequately (e.g. mean or median scores) to support the conclusions drawn?		
39) Were confidence intervals or p-values reported for the results of the hypothesized HRQoL endpoints?		

40) Did the authors adequately report missing data (e.g. due to item non-response, due to non-completion of the instrument)?		
41) Were subjects excluded from the HRQoL analysis?<br>a. If “Yes” did the investigators describe the circumstances surrounding subjects excluded from the analysis?		
42) Was the clinical significance of the HRQoL results addressed?		

Appendix 2 Data searches, study selection and data extraction: Author roles

The authors Rebecca McLeese and Zina Alfahl independently performed data searches and study screening. Judy Bradley, Katherine O'Neill and Arietta Spinou were consulted to resolve any disagreements. To ensure data extraction was in line with Spinou et al., Judy Bradley selected 10 studies at random, Rebecca McLeese and Zina Alfahl independently extracted data and this was checked by Judy Bradley and Arietta Spinou. Rebecca McLeese and Zina Alfahl independently extracted data from the remaining included studies and data extraction was checked by Judy Bradley or Katherine O'Neill. Rebecca McLeese and Zina Alfahl independently assessed the quality of all studies using a modified tool developed by Swigris et al., 2005 (see Online Supplementary Appendix 1) and data extraction was checked by Judy Bradley and Katherine O'Neill.

Appendix 3 Supplementary Methodology

Correlations meta-analysis

In order to perform meta-analysis, the bounded correlation coefficients were converted to unbounded variables using Fisher's transform $Z = \frac{1}{2}\ln\left(\frac{1+r}{1-r}\right)$, where Z is assumed to follow a normal distribution with mean equal to $\frac{1}{2}\ln\left(\frac{1+\rho}{1-\rho}\right)$ and variance equal to $\frac{1}{n-3}$, where ρ is the true correlation and n denotes the sample size of each study. The Z quantities computed for each study were subjected to meta-analytic models (Borenstein et al., 2011). The pooled estimate Z_c was extracted using a random effects model*. Finally, the meta-analytically pooled Z values were converted back to the pooled correlation using the inverse Fisher's transform $r_c = \frac{\exp(2Z_c)-1}{\exp(2Z_c)+1}$, where exp(.) denotes the exponential function.

Heterogeneity and Publication Bias

Heterogeneity between studies was tested with the standard χ^2 test. An I^2 value of 0% indicated no observed heterogeneity, while >50% indicated substantial heterogeneity (Higgins et al., 2003).The I^2 values for the treatment, placebo and total combined effect sizes were equal to 89%, 64% and 83% respectively, indicating a high level of heterogeneity (all $p<0.01$). Such heterogeneity in treatment effects is attributed to differences in study population demographics, interventions received (e.g. dose of drug), follow-up length, and other factors (Riley et al., 2011).

A random effects model was used to determine effect sizes with 95% confidence intervals (CI), to allow for differences in the treatment effect from study to study. The random effects model assumes the observed treatment effect estimates can vary across studies because of real differences in the treatment effect in each study as well

as sampling variability (chance).

To determine the presence of publication bias, we tested for asymmetry of funnel plots using a weighted linear regression in which effect sizes were plotted against their standard errors using Egger's test (Egger et al., 1997) and against their sample sizes. Should there be no publication bias, the studies should cover the area within the triangle. In specific, studies with high precision (effect sizes with low variance) are expected to appear near the average. On the contrary, studies with low precision should be spread evenly about the average covering all area of the triangle. Deviations from this pattern could be evidence of publication bias. The p-values for treatment, placebo and total combined effect sizes were equal to 0.025, 0.050 and 0.563 respectively. There was evidence that the observed symmetry in the funnel plots was marginally significant at the 5% significance level for the treatment effect, statistically significant for the placebo effect and non-significant for the total combined effect. The stability of the meta-analysis was tested by computing Rosenthal's fail safe number (Rosenthal, 1979). This is a "what if" analysis applied to studies to demonstrate how many null articles would be needed to change the statistically significant results to a statistically non-significant finding. Rosenthal's fail safe number for treatment, placebo and total effect sizes was equal to 7320, 490 and 6856.

References in online supplement.

*The advantage of random effects over fixed effects is that the former allows for heterogeneity among the studies, as was the case in the present work. Ignoring the heterogeneity can result in severely biased pooled estimates.

Appendix 4 Description of Health Related Quality of Life Questionnaires

Bronchiectasis Specific Questionnaires

Quality of Life - Bronchiectasis (QoL-B): a bronchiectasis specific HRQoL questionnaire. QoL-B version 3.1 has 37 items in 8 domains: respiratory symptoms, physical functioning, vitality, role functioning, health perceptions, emotional functioning, social functioning and treatment burden. Domain scores range from 0 to 100, and no total score is calculated. Higher scores indicate better HRQoL (Quittner et al., 2015).

Bronchiectasis Health Questionnaire (BHQ): a bronchiectasis specific HRQoL questionnaire. Rasch Analysis (a quantitative method of eliminating the instrument's items to create a linear scale) was used for its development. The questionnaire consists of 10 items and a single overall health status score is calculated. Scores range from 0 to 100. Higher scores indicate better health status (Spinou et al., 2017b).

Respiratory Specific Questionnaires

Saint George's Respiratory Questionnaire (SGRQ): was originally developed to measure HRQoL of patients with Chronic Obstructive Pulmonary Disease (COPD) and asthma. It has 50 items (76 weighted responses) across 3 domains: symptoms, activity and impact. The total score ranges from 0 to 100. Lower scores indicate better HRQoL (Jones et al., 1992).

Leicester Cough Questionnaire (LCQ): is a cough-specific questionnaire, which assesses the impact of cough on HRQoL. It was originally developed for adults with chronic cough. LCQ has 19 items with 7-point Likert response scales. LCQ has 3 domains: physical (8 items), psychological (7 items) and social (4 items). The sum of

the domain scores gives the total score which ranges from 3 to 21. Higher scores indicate better HRQoL (Birring et al., 2003).

Chronic Obstructive Pulmonary Disease Assessment Tool (CAT): was developed using Rasch Analysis for patients with COPD. It has 8 items with 0-5 response scales. The total score ranges from 0 to 40. Lower scores indicate better HRQoL (Jones et al., 2009).

Chronic Respiratory Disease Questionnaire (CRDQ): is an interviewer-administered questionnaire. It measures physical and emotional aspects of quality of life in patients with chronic respiratory disease, including COPD (>3 months in duration). It has 20 items across 4 domains: dyspnoea, fatigue, emotional function and mastery. Higher scores indicate better HRQoL (Guyatt et al., 1987).

20-Item Sino-Nasal Outcome Test (SNOT-20): was originally developed for patients with rhinosinusitis. This is a modification of the 31-item Rhinosinusitis Outcome Measure. It has 20 nose, sinus and general items. Lower scores indicate better HRQoL (Piccirillo et al., 2002).

Cough Quality of Life Questionnaire (CQLQ): is a cough-specific questionnaire. It has 28 items and a 4-point Likert response scale across 6 domains: physical complaints, psychosocial issues, functional abilities, emotional well-being, extreme physical complaints and personal safety fears. The maximum score is 112. Lower scores indicate better HRQoL (French et al., 2002).

Seattle Obstructive Lung Disease Questionnaire (SOLQ): was developed to monitor HRQoL in patients with COPD. It has 29 items across 4 domains: physical function, emotional function, coping skills, and treatment satisfaction. Items are scored

on a simple linear scale, with a response of 1 representing the lowest function. The sum of all items gives a raw score that is transformed to a normalized score ranging from 0 to 100. Higher scores indicate better HRQoL (Tu et al., 1997).

Generic Questionnaires

Medical Outcomes Study 36-item Short-Form Health Survey (SF-36): a generic HRQoL questionnaire with 8 domains: physical functioning, role physical, bodily pain, general health, vitality, social functioning, role emotional, and mental health. These scales are grouped into either SF-36 Physical and SF-36 Mental Component scores that range from 0 to 100. Higher scores indicate better HRQoL (Ware and Sherbourne, 1992).

Nottingham Health Profile (NHP): is a self-administered generic HRQoL measure designed to measure perceived distress related to health conditions. It has 38 items across 6 domains: sleep, mobility, energy, pain, emotional reactions and social isolation. Respondents must answer "yes" or "no", scored as either 1 or 0, respectively. Series of weights are used to score each category from 0 to 100. Lower scores indicate better HRQoL (Hunt and McEwen, 1980).

Euro Quality of Life 5-level (EQ-5D-5L): The EQ-5D was developed to measure patient HRQoL in economic evaluations and has been increasingly used as a 'stand-alone tool' (Buchholz et al., 2018). The EQ-5D-5L comprises of five dimensions: mobility, selfcare, usual activities, pain/discomfort and anxiety/depression. The digits for the five dimensions are combined in a 5-digit code that describes the respondent's health state. EQ-5D-5L health states can be summarised using the 5-digit code or represented by a single summary number (index value), which reflects how good or bad a health state is according to the preferences of the general population of a

country/region. An EQ-5D index value is derived by applying a formula that attaches values (weights) to each of the levels in each dimension. The EQ-VAS is administered with the EQ-5D-5L and records the patient's self-rated health on a vertical visual analogue scale (VAS) (0- 100), where the endpoints are labelled 'The best health you can imagine' and 'The worst health you can imagine'. The VAS can be used as a quantitative measure of health outcome that reflects the patient's own judgement. The EQ-5D-5L has largely replaced the EQ-5D-3L. The biggest change has been in refining the 'granularity' of the five dimensions by replacing the 3-category response scale with a 5-category response scale.

References in online supplement.

Appendix 5 The validation of translated health-related quality of life questionnaires

Overall, 32 studies used a translated HRQoL questionnaire. The translated questionnaires included: QOL-B, BHQ, SGRQ, LCQ, CAT, SOLQ, and SF-36. An additional 14 studies were included since the original review: translated QOL-B was used in Spain (Olveira et al., 2014a, Doña et al., 2018, Máiz et al., 2018), Germany (Ringshausen et al., 2018, Sokol et al. 2019) and Brazil (De Camargo et al., 2020); BHQ in Denmark (Gissel et al., 2020) and Korea (Kim et al., 2020); SGRQ in Egypt (Abd-Elazeem et al., 2018), China (Gao et al., 2014b); LCQ in Spain (Máiz et al., 2018), Turkey (Ozalp et al., 2019, Cakmak et al., 2020) and China (Gao et al., 2014b); SOLQ in Turkey (Bulcun et al., 2015); and SF-36 in Turkey (Bekir et al., 2017). All of these studies reported or referenced a validation of the translated questionnaire. These studies validated the translated questionnaires in patients with bronchiectasis, with only exceptions being the Arabic SGRQ (COPD-bronchiectasis overlap syndrome) (Abd-Elazeem et al., 2018).

For references see main paper and online supplement.

Appendix 6 Discriminant validity of health-related quality of life questionnaires

Bronchiectasis specific HRQoL questionnaires

QoL-B respiratory symptoms domain was most commonly used in clinical trials and was able to discriminate patients based on demographics (Polverino et al., 2020), disease severity (Olveira et al., 2014a, Quittner et al., 2015, Visser et al., 2019; Liu et al., 2019), exacerbations (Olveira et al., 2014a, Polverino et al., 2018, Dhar et al., 2019, Visser et al., 2019, Brown et al., 2019), bacteriology (Olveira et al., 2014a, Terpstra et al., 2019, Sibila et al., 2019, Polverino et al., 2018, Dhar et al., 2019, Brown et al., 2019, Ewen et al., 2019, Asakura et al., 2020), sputum volume (Polverino et al., 2018, Dhar et al., 2019), dyspnoea (Dhar et al., 2019, Polverino et al., 2018), haemoptysis (Olveira et al., 2014a) and exercise capacity (Quittner et al., 2015). Discriminant validity for the other 7 domains was also reported. Studies reported that some of the QoL-B domain scores were able to discriminate patients based on demographics (Polverino et al., 2018, Finch et al., 2017, Olveira et al., 2014a, McCullough et al., 2011, Terpstra et al., 2019), disease severity (Dhar et al., 2019, Quittner et al., 2014, Quittner et al., 2015, Olveira et al., 2014a, Visser et al., 2019, Liu et al., 2019), exacerbations (Dhar et al., 2019, Visser et al., 2019, Olveira et al., 2014a, Alcaraz-Serrano et al., 2020), bacteriology (Polverino et al., 2018, Visser et al., 2019, Olveira et al., 2014a, Asakura et al., 2020), haemoptysis (Olveira et al., 2014a), dyspnoea (De Camargo et al., 2020), adherence to treatments (McCullough et al., 2014, McCullough et al., 2015, Polverino et al., 2018) and exercise capacity (Quittner et al., 2015). The BHQ was able to discriminate patients based on demographics (Choi et al., 2020, Diggins et al., 2020), disease severity, exacerbations and hospital admissions in the preceding 12 months (Spinou et al., 2017b).

Respiratory specific HRQoL questionnaires

Studies reporting SGRQ total scores were able to discriminate based on demographics (Bellelli et al., 2016, Chalmers et al., 2014), disease severity (Chalmers et al., 2014, Eshed et al., 2007, Martinez-Garcia et al., 2005, McDonnell et al., 2016), exacerbations (Altenburg et al., 2016, Murray et al., 2009a, Chalmers et al., 2014, Martinez-Garcia et al., 2005, Guan et al., 2014, Chalmers et al., 2018, Brown et al., 2019), bacteriology (Chalmers et al., 2014, Martinez-Garcia et al., 2005, Wilson et al., 1997a, Hill et al., 2015a, Araujo et al., 2018, Aliberti et al., 2016, Brown et al., 2019, Basavaraj et al., 2019), sputum volume (Chan et al., 2002, Martinez-Garcia et al., 2005), dyspnoea (Chalmers et al., 2014, Wilson et al., 1997b), chronic rhinosinusitis (Guilemany et al., 2009), sleep quality (Gao et al., 2014a), anxiety and depression (Gao et al., 2018) and exercise capacity (Guan et al., 2015a). Studies reporting SGRQ domain scores were able to discriminate based on disease severity (McDonnell et al., 2016, Eshed et al., 2007), exacerbations (Murray et al., 2009a, Guan et al., 2015b, Chalmers et al., 2018), bacteriology (Wilson et al., 1997a), sputum volume (Chan et al., 2002), chronic rhinosinusitis (Guilemany et al., 2009), wheeze (Wilson et al., 1997a), sleep quality (Gao et al., 2014a), and exercise capacity (Guan et al., 2015a). SGRQ total and domain scores were able to discriminate patients with depression (Gao et al., 2018) and SGRQ total and impact score was able to discriminate patients with anxiety (Gao et al., 2018).

LCQ total score was able to discriminate patients based on disease severity (Guan et al., 2014, Munoz et al., 2016, Murray et al., 2009b), exacerbations (Altenburg et al., 2016, Munoz et al., 2016, Murray et al., 2009b), bacteriology (Aliberti et al., 2016, Guan et al., 2014, Wang et al., 2018), sputum purulence and volume (Guan et al., 2014), duration of symptoms (Guan et al., 2014), patients with/without airway reflux

(Mandal et al., 2013) and with/without capsaicin cough hypersensitivity (Guan et al., 2014). LCQ domain scores were able to discriminate patients based on disease severity (Murray et al., 2009b) and exacerbations (Munoz et al., 2016, Murray et al., 2009b). The CAT was able to discriminate patients based on disease severity (Lanza et al., 2018, De la Rosa Carrillo et al., 2020), exacerbation rate (Finch et al., 2020), bacteriology (Feliu et al., 2018), dyspnoea (Lanza et al., 2018), and exercise capacity (Lanza et al., 2018). The CRDQ was able to discriminate patients based on exacerbation status (Courtney et al., 2008). The SNOT-20 total score was able to discriminate patients with/without chronic rhinosinusitis (Guilemany et al., 2009).The SOLQ physical function and coping skills domains were able to discriminate patients with/without bronchial hyperreactivity (Bulcun et al., 2015). Discriminant validity was not reported for the CQLQ.

Generic HRQoL questionnaires

SF-36 total score was able to discriminate patients based on disease severity (Bekir et al., 2017), exacerbations (Altenburg et al., 2016) and depression (Bekir et al., 2017). SF-36 domain scores were able to discriminate patients based on demographics (Bulcun et al., 2015), bacteriology (Wilson et al., 1997a), sputum volume (Chan et al., 2002), depression (Bekir et al., 2017), patients with/without chronic rhinosinusitis (Guilemany et al., 2009) and with/without bronchial hyperreactivity (Bulcun et al., 2015). The EQ-5D-5L Visual Analogue Scale (VAS) component was able to discriminate patients based on disease severity (Zanini et al., 2015a). The EQ-5D index score and mobility, usual activity, pain/discomfort and anxiety/depression component scores were able to discriminate patients based on demographics (Yang et al., 2020). Discriminant validity was not reported for the NHP.

For references see main paper and online supplement.

Appendix 7 Floor/ceiling effects and missing data

Floor and ceiling effects were reported for QoL-B (Quittner et al., 2014, Quittner et al., 2015, Olveira et al., 2014a, Sokol et al., 2019, De Camargo et al., 2020), BHQ (Spinou et al., 2017b), SGRQ (Wilson et al., 1997a, Martínez García et al., 2005), LCQ Spanish (Munoz et al., 2016), LCQ Mandarin (Gao et al., 2014b), CAT (De la Rosa Carrillo et al., 2020, Qi et al., 2019) and CRDQ (Vodanovich et al., 2015). All studies met the standards for floor effects (<15% participants) for all questionnaires. For QoL-B, 4 out of 5 studies failed to meet the standards for ceiling effects (>15% participants) (Quittner et al., 2014, Quittner et al., 2015, Olveira et al., 2014a, De Camargo et al., 2020). There were ceiling effects in the emotional functioning (24%) (Quittner et al., 2015), social functioning (22%) (Olveira et al., 2014a), 19% (De Camargo et al., 2020), role functioning (18%) (Olveira et al., 2014a) and treatment burden (17%) (Quittner et al., 2014), (16%) (De Camargo et al., 2020) domains. For LCQ in the social domain, 1 out of 2 studies failed to meet the standards for ceiling effects (>15%) (Munoz et al., 2016), however, no ceiling effects were present for the total or other domain scores. All other questionnaires met the standards for ceiling effects (<15% participants) for total or domain scores. Missing data were reported for QoL-B (up to 8.7%) (Quittner et al., 2014, Quittner et al., 2015), BHQ (7.3%) (Spinou et al., 2017b), SGRQ (1.3-7.9%) (Martínez García et al., 2005, Chan et al., 2002), and CAT (1-38%) (Brill et al., 2015, De la Rosa Carrillo et al., 2020).

For references see main paper and online supplement.

Table E1 Inclusion and Exclusion Criteria and Psychometric Properties Assessed

Inclusion Criteria	Exclusion Criteria
– Adult patients (≥18 years old)<br>– Clinical and/or radiological diagnosis of bronchiectasis<br>– Studies published in English language<br>– Studies:<br>– Reporting psychometric properties of HRQoL questionnaires and/or association of HRQoL with other clinical measures<br>– Acute exacerbations if they meet inclusion criteria<br>– Mixed populations providing results are distinguishable by age/disease category<br>– Interview/ focus group methodology if they reported on the psychometric properties of HRQoL	– Diagnosis of cystic fibrosis<br>– Reviews<br>– Protocols<br>– Abstracts already covered by full text manuscripts

Psychometric Properties
– Internal consistency (Cronbach's α) and test-retest reliability (intraclass correlation coefficient), including timescale of test-retest reliability.<br>– Translational validity.<br>– Discriminant ability based on demographics, disease severity, exacerbations and hospital admissions, sputum and bacteriology, signs and symptoms, adherence to treatment, exercise capacity.<br>– Associations with other clinical measures: demographics (age, sex, BMI), symptoms (cough, dyspnoea, wheeze, fatigue, anxiety, depression), disease severity (BSI, FACED, CT bronchiectasis score, CT lung score), lung function and exercise capacity (FEV_1 & FVC % predicted, exercise capacity), sputum bacteriology (bacteriology, *Pseudomonas aeruginosa* (*Pa*) colonization, inflammatory markers), healthcare utilisation (infection/exacerbation rate, hospital admissions rate), sputum volume, oxygen saturation and co-morbidities.<br>– Associations with other HRQoL questionnaires.<br>– Responsiveness (effect sizes).<br>– Floor/ceiling effects and missing data.<br>– Minimal clinically important differences (MCID).

Table E2 Search terms and number of new studies retrieved in each database published
‘non-cystic fibrosis bronchiectasis’ used instead of ‘bronchiectasis’

**Keywords**	**Pubmed**	**Embase**	**Medline**	**Cochrane**	**PsycINFO**
Bronchiectasis AND quality of life	243	#198	345	81	2
Bronchiectasis AND QOL	40	157	53	19	0
Bronchiectasis AND health status	142	71	33	11	0
Bronchiectasis AND psychometrics	7	2	9	3	0
Bronchiectasis AND well being	#117	17	6	8	0
Bronchiectasis AND psychology	47	19	55	6	0
Bronchiectasis AND daily living	4	19	7	2	0
Bronchiectasis AND HRQoL	238	55	29	3	0
Bronchiectasis AND questionnaire	258	604	158	36	1
Bronchiectasis AND validation	39	145	56	48	0
Bronchiectasis AND validity	19	66	26	48	0
**Total**	1154	1353	777	265	3
**Total after duplicates removed**	1571				

The search was conducted in five databases: Pubmed, Embase, Medline, Cochrane Library and PsycINFO. The keywords for search were: non cystic fibrosis bronchiectasis/bronchiectasis, quality of life/QOL/HRQoL, health status, well-being, daily living, questionnaire, validation/validity, psychology, and psychometrics. Search was from 6th November 2014 up until 31st December 2020.
Pubmed: searched all fields, Embase: searched .mp., Medline: searched .mp., Cochrane: searched keyword, title, abstract, PsycINFO: searched .mp.

Table E3 Characteristics of new studies included in the literature review

Author 1st, year	HRQoL tools	Country	N	Age (year)	Female (%)	FEV$_1$ % (pred)	Most common bronchiectasis aetiology	(%)	Relevant study objectives
McCullough et al., 2014, McCullough et al., 2015	QoL-B	UK	75	64	68	61	Post-infective	43	Determine the association between adherence to treatment and health outcomes (pulmonary exacerbations, lung function and HRQoL).<br><br>Determine if baseline beliefs about treatment clinical factors and HRQoL predicted adherence to treatment.
Olveira et al., 2014a, Doña et al., 2018	QoL-B (Spanish)	ES	30	56	60	66	NR		Assess effects of PR alone versus PR plus an oral nutritional supplement, enriched with HMB, on exercise capacity, respiratory symptoms, HRQoL, psychological symptoms, and functional respiratory parameters.
Chaurasia et al., 2017	QoL-B	IN	44	NR	NR	NR	NR		Determine predictors of HRQoL.<br><br>Relationship between HRQoL and other outcome parameters.
Finch et al., 2017	QoL-B	EU	8389	68*	58	NR	NR		Determine sex differences in HRQoL scores.
Polverino et al., 2018, Polverino et al., 2020	QoL-B	EU	11204	65	60		Idiopathic	37	Relationship between HRQoL and clinical factors.
Ringshausen et al., 2018, Sokol et al., 2019	QoL-B (German)	DE	1043	61*	59	NR	NR		Validate the German version of the QoL-B questionnaire.<br><br>Relationship between the QoL-B scales and symptom burden as well as markers of disease severity.
Dhar et al., 2019	QoL-B	IN	2195	56	43	61	Post-tuberculosis	36	To describe the characteristics, severity of disease, microbiology, and treatment of patients with bronchiectasis in India.
Ewen et al., 2019	QoL-B	DE	1000	61	64	NR	Primary Ciliary Dyskinesia	26	Compare subjects with a history of nontuberculous mycobacterium and/or a positive respiratory culture at baseline to subjects without such history and no isolation.
Haworth et al., 2019, Chalmers et al., 2020a	QoL-B	AU,CA,DE, HU,IRL,IL, IT,KR,LV, PL,RO,ES,TW,UK,US,ZA	582	64	60	67	NR		Investigate efficacy and safety of inhaled liposomal ciprofloxacin in the reduction of the burden of *Pa* infection.<br><br>To examine whether respiratory symptoms improved during on-treatment periods and the

		, FR, GE, PE							relationship of changes in QOL-B respiratory symptoms domain to changes in bacterial load using a mixed model repeated measures approach.
Liu et al., 2019, Tong et al., 2020	QoL-B	AU	131	62	74	68	NR		Confirm the reliability and validity and to establish and evaluate the MCID and sensitivity of the QoL-B within the Australian population.
Magge et al., 2019	QoL-B	US	19	67	84	68	Idiopathic	79	Determine improvements in HRQoL following treatment.
Pathak et al., 2019	QoL-B	UK	7	57*	56	NR	NR		Evaluate whether the content of QOL-B respiratory symptom domain relates to patients experiences of symptoms included in the dose finding study for tobramycin inhalation powder in non-CF bronchiectasis, iBEST-1.
Shoemark et al., 2019	QoL-B	ES, UK	124	69*	57*	69*	Idiopathic	52	Test the predictive value of a commercially available point-of-care neutrophil elastase assay (NEATstik®) to predict clinically relevant outcomes in patients with bronchiectasis.
Sibila et al., 2019	QoL-B	UK	189	65	57	72	Idiopathic	46	Determine the association between bacterial load and HRQoL, disease severity, FEV1, and airway neutrophil biomarkers.
Terpstra et al., 2019	QoL-B v3.1	NL	200	70	57	84	Post-infective	40	Determine if HRQoL is related to the specific aetiologies and disease severity.
Visser et al., 2019	QoL-B	AU	589	71*	71	75*	Idiopathic	33	Assess the influence of disease severity and exacerbation phenotype on HRQoL and health-care utilisation.
Asakura et al., 2020	QoL-B	JP	963	69	80		Idiopathic	73	Clarify the aetiology and disease burden in Japanese patients with non-CF bronchiectasis and nontuberculous mycobacterial pulmonary disease.
Li et al., 2020	QoL-B	CN	128	57	65.6	72.2	Post-infective	46	Identify the role of serum albumin/ pre-albumin level and BMI in reflecting the disease severity and clinical presentation of patients with non-CF bronchiectasis.
Shteinberg et al., 2020	QoL-B	IL	49	NR	NR	NR	NR		Compare effects of two methods of airway clearance- autogenic drainage and oscillating positive airway pressure on LCI, spirometry, sputum quantity, and QoL.
Bradley et al., 2015	QoL-B, LCQ	UK	55	63	60	76	NR		Relationship between sedentary behaviour and physical activity and correlates of these behaviours.

									Relationship between physical activity levels and clinical characteristics (disease severity, exercise capacity, HRQoL and other symptoms of their disease) and constructs of the transtheoretical model (stages of change, self-efficacy, decisional balance and processes of change).
Aksamit et al., 2018, De Soyza et al., 2018, Speck et al., 2018	QoL-B, SGRQ	US	521	60	58	56	Idiopathic	54	Evaluate the efficacy and safety of ciprofloxacin dry powder for inhalation in prolonging time to first exacerbation and reducing the frequency of exacerbations.
Máiz et al., 2018	QoL-B-Sp-V3.0, LCQ	ES	137	63	64	76	Post-infective	42	Evaluate the tolerability and effectiveness of the combination of 7% HS supplemented with 0.1% HA in patients were intolerant to treatment with 7% HS.
Spinou et al., 2018	QoL-B, BHQ	UK	43	56*	70	NR	NR		Assess convergent validity, completion rates and internal consistency of the QoL-B and BHQ.
Bondarenko, 2019	QoL-B, BHQ, LCQ	AU	60	NR	NR	73	NR		Relationship between symptoms of chronic rhinosinusitis, HRQoL and health status<br><br>Identify predictors of HRQoL.
Brown et al., 2019	QoL-B, SGRQ	UK	1403	65	62	NR	NR		Evaluate the convergent validity of QoL-B and SGRQ questionnaires.
Galli et al., 2019, Santos et al., 2019	QoL-B, LCQ, SF-36	BR	26	56	NR	NR	NR		Evaluate anxiety, depression, and HRQoL in patients with bronchiectasis, using QoL-B, and correlate with disease severity.
Herrero-Cortina et al., 2019	QoL-B, LCQ	ES	20	60	65	NR	NR		Determine the efficacy of a 12-month home-based airway clearance programme on cough severity in people with bronchiectasis.
Alcaraz-Serrano et al., 2020	QoL-B, LCQ	ES	64	63	67	72	Post-infective	45	Investigate the association between (i) any physical activity variable and (ii) sedentary time with hospitalisation due to exacerbation in adults with bronchiectasis.
Chalmers et al. 2020b	QoL-B, SGRQ, LCQ	AU, BE, BG, DK, DE, IT, NL, NZ, PL, SG, KR, ES, UK, US	256	64	68	68	NR		Examine whether brensocatib reduces the incidence of exacerbations of bronchiectasis.
De Camargo et al., 2020	QoL-B, SGRQ	BR	108	48	56.5	55	NR		Test the psychometric properties of the Brazilian Portuguese version of the QoL-B.
Hester et al., 2020	QoL-B, SGRQ, EQ-	UK	62	65*	61	68*	Idiopathic	35	Establish the feasibility of conducting a multi-centre RCT to determine effect of the information resource on understanding, self-management and

	5D-5L								health outcomes.
Choi et al., 2020	BHQ	KR	436	NR	NR	NR	NR		Compare respiratory symptoms, co-morbidities, lung function, radiologic features, QoL, exacerbations, and disease severity in bronchiectasis patients with asthma, versus those without asthma
Diggins et al., 2020	BHQ	NZ	141	67	57	69	NR		Assess bronchiectasis severity and QoL in patients in the New Zealand Bronchiectasis registry.
Spinou et al., 2017a	BHQ, SGRQ, LCQ	UK	54	61*	69	71	Idiopathic	46	Investigate cough frequency with an objective cough monitor and assess its association with self-reported cough severity and HRQoL.
Spinou et al., 2017b	BHQ, SGRQ	UK	206	59	65	75*	Idiopathic	41	Develop and validate a brief and easy to administer and interpret HRQoL tool with a single overall score.
Nicolini et al., 2019	BHQ, LCQ, CAT	IT	60	NR	NR	NR	NR		Compare bronchiectasis patients treated with SmartVest and a novel machine (RespIn 11) (high frequency chest wall oscillation techniques) with pharmacological therapy alone.
Gissel et al., 2020	BHQ, SGRQ	DK	111	NR	NR	NR	NR		Translate and validate the BHQ in Danish using established cross validation methods.
Kim et al., 2020	BHQ-Korean, CAT	KR	126	64	53	60	Post-tuberculosis	45	Report on the process of the development of the Korean version of the BHQ using a standardized methodology.
									Evaluate the validity of the Korean version of the BHQ in Korean patients with bronchiectasis.
Liu et al., 2014	SGRQ	CN	52	48	47	67	NR		Assess the effect of roxithromycin on inflammation media in induced sputum, dilated bronchial wall thickness, HRQoL and exacerbations.
Guan et al., 2015b	SGRQ	CN	49	43	61	66	Idiopathic	39	Examine variations in airway and systemic inflammation, spirometry, and HRQoL during steady state, exacerbations, and convalescence.
Hill et al., 2015a, Hill et al., 2015b	SGRQ	UK	286	NR	NR	NR	Idiopathic	43	Compare clinical outcomes, including HRQoL with *Pa* infection who cleared the bacteria vs those who were never infected.
Khodosh et al., 2015	SGRQ	UA	45	NR	NR	NR	NR		Investigate the efficacy of combined inhaled drugs on HRQoL, microbiological and functional parameters.
Panyarath, 2016	SGRQ	TH	30	NR	NR	NR	NR		Determine the effects of roflumilast on

									exacerbation frequency.
Svenningsen et al., 2017	SGRQ	CA	15	69	73	69	NR		Evaluate and compare bronchiectasis airways observed on CT with pulmonary ventilation using MRI.<br>Investigate the effect of ACT on regional MRI ventilation measurements.
Balteanu et al., 2017	SGRQ	RO	31	60	24	50	NR		Analyze effects of a rehabilitation program on HRQoL, physical performance and pulmonary function.
Chalmers et al., 2017	SGRQ	UK	433	67*	61	72*	Idiopathic	45	Determine the association of elastase activity and desmosine with exacerbations and lung function decline.
Dal Corso et al., 2017	SGRQ	BR	39	46	NR	48	NR		Investigate the impact of HBPR on functional capacity, HRQoL, and peripheral muscle strength.
Pumphrey et al., 2017	SGRQ	UK	257	67*	58	72*	NR		Determine if NETs are present in the airway.<br>Determine if NETs are associated with disease severity.
Abd-Elazeem et al., 2018	SGRQ (Arabic)	ES	68	41	41	28	NR		Impact of overlapping between COPD and bronchiectasis on clinical, physiological, radiological parameters, inflammatory markers and HRQoL.
Cartlidge et al., 2018	SGRQ	UK	94	66	54	72	Idiopathic	70	Assess ISWT as a reliable indicator of disease severity, its response to treatment as an objective clinical end point, and its MCID.
Atalay et al., 2019	SGRQ	TR	13	50	NR	NR	NR		Compare the effect of aerobic exercise with whole body vibration on exercise capacity, respiratory function, dyspnoea perception and QoL in patients with bronchiectasis.
Basavaraj et al., 2019	SGRQ	US	118	NR	NR	NR	NR		Characterize differences in clinical phenotype among bronchiectasis subjects with and without nontuberculous mycobacterium positive cultures.
Drew et al., 2019	SGRQ	US	227	61	71	NR	NR		Understand the risk of mortality among patients with bronchiectasis.
Cordova-Rivera et al., 2019	SGRQ	AU	61	68*	87	77	NR		Relationship between HRQoL and extrapulmonary characteristics, including physical activity, muscle strength, comorbidities, systemic inflammation and anxiety and depression with HRQoL.
Pehlivan et al., 2019	SGRQ	TR	19	48*	63	56	NR		Investigate the effects of PR on the level of physical activity and general clinical status.

Raats et al., 2019	SGRQ	BE	60	62	57	NR	Idiopathic	49	Explore the additional value of calprotectin as a biomarker for exacerbation in bronchiectasis.
Varol et al., 2019, Sahin et al., 2019	SGRQ	TR	183	NR	NR	NR	NR		Compare the two validated bronchiectasis scoring systems according to the 6MWD and SGRQ questionnaire.
Watz et al., 2019	SGRQ	UK, DE, IT, ES	94	66	47	56 (pre-bronchodilator), 59 (post-bronchodilator)	Post-infective	52	Assess the safety and efficacy of treatment with oral BAY 85-8501.
Cakmak et al., 2020	SGRQ (Turkish)	TR	41	38	68	69	NR		Compare physical activity levels between patients with bronchiectasis and healthy individuals.
Gao et al., 2020	SGRQ	UK	333	68*	63	72*	Idiopathic	48	Examine the relationship between daily symptoms and exacerbations over 12 months after baseline and to investigate the time to first exacerbation.
Liu et al., 2020	SGRQ	CN	115	NR	NR	NR	NR		Determine if exhaled breath condensate pH could be used as a validated biomarker of disease severity in bronchiectasis.
McKeough et al., 2020	SGRQ	AU	33	74	48	60	NR		Compare levels of self-reported sedentary behaviour in people with COPD and in people with bronchiectasis.
Varughese et al., 2020	SGRQ	IN	82	56	53.7	NR	NR		Compare the severity of bronchiectasis as measured by FACED and BSI scores with the QoL measured by SGRQ scores.
Gao et al., 2014b, Guan et al., 2015c	SGRQ, LCQ (Mandarin)	CN	144	45	60	67	NR		Validate the Mandarin Chinese version of the LCQ (LCQ-MC).<br>Investigate differences in inflammatory indices, lung function and HRQoL associated with different PPMs.
Guan et al., 2014, Guan et al., 2016, Guan et al., 2015a, Gao et al., 2018	SGRQ, LCQ	CN	163	46	63	67	Post-infective	28	Relationship between 6MWD and clinical parameters, including demographics, lung function, aetiology, sputum bacteriology, dyspnoea scale and HRQoL.<br>Identify the factors associated with reduced 6MWD.<br>Investigate the factors associated with anxiety and depression.<br>Evaluate the effect of anxiety and depression on HRQoL.

Rogers et al., 2014	SGRQ, LCQ	AU	86	63	58	68	NR		Assess effects of erythromycin on the composition of respiratory microbiota.<br>Assess whether this effect was associated with changes in clinical outcome measures of pulmonary exacerbations, resulting in changes in airway microbiology.
Brill et al., 2015	SGRQ, CAT	UK	32	61	84	78	Post-infective	50	Investigate changes in lung function, symptoms, health status and inflammation before, during and after community treated exacerbations.
Gao et al., 2015	SGRQ, LCQ, CAT	CN	119	45	65	65	Idiopathic/post-infective	59	Investigate the clinical differences between virus-positive and virus-negative exacerbations.
Aliberti et al., 2016, Araujo et al., 2018, Chalmers et al., 2018, Bellelli et al., 2016	SGRQ, LCQ	UK, IRL, BE, IT, GR, RS, IL, ES	2596	67*	61	74*	Idiopathic	42	Evaluate clinical, radiological, microbiological, and functional characteristics, along with the disease severity and clinical outcomes, in adults and elderly patients with bronchiectasis.
McDonnell et al., 2016	SGRQ, QoL-B	UK, IT	744	70	60	74	NR		Evaluate the predictive ability of the BSI and FACED score in assessing clinically relevant disease outcomes.
Bedi et al., 2017	SGRQ, LCQ	UK	32	65	66	57	Idiopathic	72	Assess whether atorvastatin could reduce cough severity and inflammation in patients with *Pa.*<br>Assess the mechanisms (in vitro studies) by which statin drugs may modulate neutrophilic inflammation.
Dudgeon et al., 2017	SGRQ, LCQ, QoL-B	UK	70	NR	NR	NR	NR	NR	Determine what contributes to HRQoL in bronchiectasis patients and to gather patient views and opinions on how existing questionnaires reflect their HRQoL. Investigate determinants of HRQoL, and the extent to which current questionnaires capture disease impact.
Kumar et al., 2017	SGRQ, LCQ	IN	42	NR	NR	NR	NR		Determine efficacy of a PR program.
Lanza et al., 2018	SGRQ, CAT	BR	100	48*	59	52	NR		Validate the CAT questionnaire Relationship between HRQoL and disease severity, dyspnoea, aerobic and functional capacity, physical activity.
Munoz et al., 2018	SGRQ, LCQ	ES	44	65	52	61	Idiopathic	39	Evaluate the efficacy of ELTGOL technique and daily productive cough.
Chalmers et al., 2019, Finch et al., 2020	SGRQ, CAT, LCQ	UK	27	68	74	81	NR		Determine benefits of rehabilitation following exacerbations.

Wong et al., 2019	SGRQ, QoL-B	NZ	36	NR	NR	NR	NR		Assess the anti-inflammatory effects of oral and transdermal clonidine.
Brockwell et al., 2020	SGRQ, EQ-5D-3L	UK	220	67	69	79	NR		Determine whether, in people with bronchiectasis, the use of our bronchiectasis patient self-management plans – Bronchiectasis Empowerment Tool, compared to standard care, would improve self-efficacy.
Bulcun et al., 2015	SOLQ (Turkish), SF-36	TR	78	48	59	79	NR		Validate the SOLQ.<br>Compare the HRQoL of subjects with bronchiectasis and bronchial hyper-responsiveness with the HRQoL of those without to identify the effective factors in HRQoL.
Vodanovich et al., 2015	CRDQ, SGRQ, LCQ	AU	85	64	72	73	NR		Validate the CRDQ.
Patel et al., 2019, Patel et al., 2020	CRDQ	UK	213	70	64	68	NR		Examine the response to PR in term of change in exercise capacity and HRQoL.<br>Examine adherence and completion rates.
Abdelhalim et al., 2016	LCQ	ES	30	52	33	56	NR		Compare efficacy of twice daily physiotherapy using active cycles of the breathing method with postural drainage vs. conventional chest physiotherapy.
Herrero-Cortina et al., 2016	LCQ	ES	31	60	71	63	Idiopathic	48	Determine effects of mucus clearance and chronic expectoration.<br>Evaluate differences between techniques related to the impact on cough, lung function and patient preference.
Herrero-Cortina et al., 2018a	LCQ	ES	28	64	64	61	Post-infective	43	Compare HA+HS, HS alone vs. IS and analyze whether a combined session of saline soutions and ACT is better than saline soutions alone in enhancing sputum quantity.<br>Evaluate the short-term tolerability and safety of HA+HS vs. HS alone.
Herrero-Cortina et al., 2018b	LCQ	ES	12	61	NR	NR	NR		Assess the ability of computerized respiratory sound, crackles and wheezes, to significantly change following an airway clearance techniques (ACTs) programme.<br>Analyse their responsiveness.
Wang et al., 2018	LCQ	CN	1188	57*	55	73	NR		Evaluate the distribution, characteristics and prognostic value of *Pa.*

Yildiz et al., 2018	LCQ	TR	41	44	66	70	Childhood illnesses	76	Describe determinants of ISWT distance as a maximal exercise capacity measurement.
Ozalp et al., 2019	LCQ (Turkish)	TR	45	44	73	73	Childhood infection	60	Explore effects of high-intensity IMT and low-intensity IMT on exercise capacity.
Hall et al., 2015	LCQ, CAT	AU	18	72	78	64	NR		Determine whether the benefits of 2 respiratory physiotherapy outpatient sessions were sustained over time.
Munoz et al., 2016	LCQ (Spanish), SGRQ	ES	259	58	60	66	Post-infective	36	Validate Spanish version of the LCQ.
Bartley et al., 2018	LCQ, SNOT-20	NZ	32	60	56	66 (pre-bronchodilation)<br>70 (post-bronchodilation)	NR		Assess serum 25(OH)D levels in a New Zealand bronchiectasis cohort.<br>Assess the effect of vitamin D3 supplementation on their 25(OH)D levels.
Key et al., 2019	LCQ	UK	6	72	33.4	57	NR		Assess cough as a key treatment target by examining cough rates and investigating the association between day and night time cough rates and cough-related quality of life (LCQ) and sputum volume.
Ferri et al., 2020	LCQ	IT	119	65*	80	NR	Idiopathic	57	Investigate if cough may affect sleep quality and evaluate its correlation with sleep impairment.
Mese et al., 2020	LCQ	TR	26	NR	NR	NR	NR		Investigate the relationship between walking speed, disease severity, exercise capacity, physical activity level, muscle strength and QoL in patients with bronchiectasis.
Zhong et al., 2020	LCQ	CN	27	52	48	58	NR		Investigate whether inhaled 0.9% normal saline by ultrasonic nebuliser had an effect in stable non-CF bronchiectasis patients with purulent sputum.
Ailiyaer et al., 2018	CAT	CN	152	57	58	56	Post-infective	65	Determine the efficiency and adverse effects of nebulized amikacin treatment along with intravenous antibiotic therapy.
Feliu et al., 2018	CAT	ES	108	NR	NR	NR	NR		Determine the utility of CAT questionnaire in the diagnosis of bacterial colonization.
Qi et al., 2019	CAT	CN	161	55	60	62	Idiopathic	60	Assess effects of oral N-acetylcysteine on exacerbations and HRQoL.
Sliwinski et al., 2019	CAT	PL	21	65	NR	NR	NR		Compare the clinical benefits of Simeox technology versus manual Chest Physiotherapy in non-CF bronchiectasis patients hospitalized for

									severe exacerbation who require airway clearance to reduce pulmonary congestion.
De la Rosa Carrillo et al., 2020	CAT	ES	96	62	79	79	Post-tuberculous	14	Validation of the CAT in bronchiectasis.
Finch et al., 2020	CAT	UK	83	71	54	52	NR		Validate the CAT questionnaire for use in bronchiectasis and to determine the minimum clinically important difference.
Artaraz et al., 2020	CAT, LCQ, SGRQ	UK	21	68	76	79	NR		Develop a novel symptom diary for bronchiectasis symptom burden and detection of exacerbations, named the BEST diary.
Bekir et al., 2017	SF-36 (Turkish)	TR	90	45*	59	NR	NR		To detect the impact of disease on HRQoL.<br>Relationship between psychological status and disease severity indexes such as BSI and FACED.
Machado et al., 2018	SF-36	BR	70	55	69	48	Idiopathic	44	Assess mortality rates and related factors.<br>Test the ability of BSI and FACED scores in predicting mortality.
Üzmezoğlu et al., 2018	SF-36	TR	40	54	55	66	NR		Compare the efficacy of home-based respiratory physiotherapy, the Flutter® device or ACT, on symptoms, sputum production, perception of dyspnoea, pulmonary functions, and HRQoL.
Saka et al., 2017	NHP	TR	17	NR	NR	NR	NR		Compare the differences for dyspnoea at activities of daily living, depression and physical activity level between the patients with asthma and bronchiectasis.
Zanini et al., 2015a	EQ-5D VAS	IT	108	71	55	76	NR		Examine effects of a PR program.<br>Determine predictors of a positive effect of a PR program.
dos Reis et al., 2018	EQ-5D-3L	BR	122	NR	72	NR	NR		Analyze HRQoL through descriptive system.
Yang et al., 2019, Yang et al., 2020	EQ-5D-5L	KR	78	55	41	82	NR		Evaluate the factors associated with bronchiectasis subjects compared with the general population without bronchiectasis and to investigate the prevalence of bronchiectasis and evaluate disease burden in terms of symptoms, QoL, and socioeconomic status by comparing subjects with bronchiectasis and those with COPD or controls using national representative data in Korea.

Data presented as means unless otherwise stated. *Median

Studies presented in the same row were combined for meta-analysis.
ACT, airway clearance therapy; AU, Australia; BE, Belgium; BHQ, Bronchiectasis Health Questionnaire; BR, Brazil; BSI, Bronchiectasis Severity Index; BG, Bulgaria; CA, Canada; CN, China; COPD, Chronic Obstructive Pulmonary Disease; CAT, COPD Assessment Test; CRDQ, Chronic Respiratory Disease Questionnaire; CT, computerised tomography; DE, Germany; DK, Denmark; ELTGOL, slow expiration with the glottis opened in the lateral posture; EQ-5D (VAS), EuroQoL-5D (Visual Analogue Scale); ES, Spain; FACED, an acronym for FEV_1, age, chronic colonisation, extension and dyspnoea; FEV_1, forced expiratory volume in one second; FR, France; GE, Georgia; GR, Greece; HA, hyaluronic acid; HMB, beta-hydroxy-beta-methylbutyrate; HRQoL health-related quality of life; HS, hypertonic saline; HU, Hungary; IL, Israel; IMT, inspiratory muscle training; IN, India; IRL, Ireland; IS, isotonic saline; ISWT, increment shuttle walk test; IT, Italy; KR, South Korea; LV, Latvia; LCQ; Leicester Cough Questionnaire; MCID, minimally clinically important difference; MRI, magnetic resonance imaging; NET, neutrophil extracellular trap; NHP, Nottingham Health Profile; NL, Netherlands; NR, not reported; NZ, New Zealand; *Pa*: *Pseudomonas aeruginosa*; PE, Peru; PL, Poland; PPMs, potentially pathological microorganism; PR, pulmonary rehabilitation; QoL-B, Quality of Life-Bronchiectasis Questionnaire; RO, Romania; RS, Serbia; SGRQ, St. Georges Respiratory Questionnaire; SF-36, 36-Item Short Form Survey; SG, Singapore; SNOT-20 Sino-Nasal Outcome Test; SOLQ, Seattle Obstructive Lung Disease Questionnaire; TH, Thailand; TR, Turkey; TW, Taiwan; UA, Ukraine; UK, United Kingdom; US, United States of America; 6MWD, six-minute walking distance; 25(OH)D, 25-hydroxyvitamin D; ZA, South Africa.

For references see main paper and online supplement.

Table E4. Average health-related quality of life scores for questionnaires.

Table E4-a. Quality of Life - Bronchiectasis (QoL-B)

**Author 1st, year**	**N**	**QoL-B**							
		**Resp. S.**	**Phys. F.**	**Vitality**	**Role F.**	**Health P.**	**Emot. F.**	**Soc. F.**	**Treat. B.**
McCullough, 2011	71	53.0	31.0	37.0	46.0	39.0	73.0	42.0	56.0
Olveira, 2014a, Olveira, 2014b	207	70.7	57.5	57.6	70.4	46.5	71.0	72.4	67.1
Quittner, 2014	89	49.5	44.7	46.7	58.4	42.1	79.8	47.8	66.2
Quittner, 2015	542	56.0	51.2	49.9	63.0	44.7	77.8	54.0	65.6
Alcaraz- Serrano, 2020	64	76.3	57.3	58.6	75.5	52.9	73.7	68.2	69.8
Bradley, 2015	55	70.0	59.0	63.0	56.0	45.0	83.0	60.0	39.0*
Brown, 2019	1403	61.0	NR	NR	NR	NR	NR	NR	NR
Dhar, 2019‡	2195	63.1	NR	NR	NR	NR	NR	NR	NR
Ewen, 2019‡	1000	54.4	NR	NR	NR	NR	NR	NR	NR
Finch, 2020	83	54	NR	NR	NR	NR	NR	NR	NR
Magge, 2019	19	62.5	48.2	45.6	60.4	41.4	78.5	49.1	68.8
McCullough, 2014, McCullough 2015	75	53.0	31.0	37.0	45.0	39.0	73.0	42.0	56.0
Pathak, 2019	7	55.6†	NR	NR	NR	NR	NR	NR	NR
Polverino, 2020	13512	58.4	NR	NR	NR	NR	NR	NR	NR
Shoemark, 2019	124	59.3†	NR	NR	NR	NR	NR	NR	NR
Sibila, 2019	440	55.9	NR	NR	NR	NR	NR	NR	NR
Spinou, 2018	43	56.9	57.8	42.5	63.9	42.6	67.5	51.6	67.0
Terpstra, 2019	200	64.2	53.4	54.2	68.0	50.4	80.6	73.1	71.8
Visser, 2019	414	67.0	83.0	56.0	73.0	60.0	83.0	58.0	78.0
Aksamit, 2018	521	51.2	NR	NR	NR	NR	NR	NR	NR
De Soyza, 2018	416	54.6	NR	NR	NR	NR	NR	NR	NR
Doña, 2018, Olveria, 2016‡	28	69.3	53.0	50.3	68.0	44.9	70.3	67.7	64.8
Haworth, 2019, Chalmers, 2020‡	582	55.8	NR	NR	NR	NR	NR	NR	NR
Hester, 2020‡	59	57.5[a]	49.9	43.9	64.2	44.5	84.5	58.9	71.5[b]
**Total/Mean**	**22149**	**59.6**	**52.1**	**49.4**	**62.5**	**45.6**	**76.6**	**57.3**	**64.7**

Table E4-b. Bronchiectasis Health Questionnaire (BHQ)

Author 1st, year	n	BHQ Total
Spinou, 2017b	206	60.0
Choi, 2020‡	436	51.2
Kim, 2020	125	49.0
**Total/Mean**	**767**	**53.4**

Table E4-c. St George's Respiratory Questionnaire (SGRQ).

Author 1st, year	n	SGRQ Total
Wilson, 1997a, O'Leary, 2002	111	44.4
Wilson, 1997a, Wilson, 1997b	87	44.4
Martínez-García, 2005	102	45.8
Eshed, 2007	46	41.7
Guilemany, 2009	80	34.2
Batchelor, 2011	608	42.6
Chalmers, 2014	19	19.1
Olveira, 2014a, Olveira 2014b	91	45.9
Loebinger, 2009	62	31.5
Lee, 2012	70	32.5
Girón Moreno, 2013	60	38.2
Rowan, 2014	144	32.3
Ozalp, 2012	32	45.7
Munoz, 2013	30	27.1
Bilton, 2013	343	37.2
Bilton, 2014	461	52.6
Diego, 2013	30	44.8
Drobnic, 2005	40	42.1
Haworth, 2014	144	56.5
Hernando, 2012	70	38.2
Lavery, 2011	64	49.3
Liaw, 2011	26	43.9
Maa, 2007	24	16.8
Mandal, 2012	27	39.7
Martínez-García, 2006	57	46.6
Nicolson, 2012	40	42.5
Serisier, 2013b	117	37.4
Stockley, 2013	38	58.8
Abd-Elazeem, 2018	68	70.6
Aliberti, 2016	389	39.0†
Araujo, 2018	987	41.2†
Artaraz, 2020	21	41.5
Brill, 2015	32	34.8
Brown, 2019	1403	42.2
Cakmak, 2020	41	43.9
Chalmers, 2017	433	44.3†
Cordova-Rivera, 2019	61	37.8

De Camargo, 2020	108	45.2
Drew, 2019	227	34
Finch, 2020	83	52.7
Gao, 2020	333	59.8#†
Guan, 2015a	141	33.8
Guan, 2015b‡	49	36.3
Guan, 2015c‡	144	33.2†
Guan, 2016	148	33.6
McKeough, 2020	33	45.0
Spinou, 2017a	54	40.1
Spinou, 2017b	206	43.0
Spinou, 2018	43	39.1
Chalmers, 2018‡	2572	45.3†
Bellelli, 2016‡	1258	39.8†
Hill, 2015b‡	286	44.4
Brockwell, 2020‡	115	42.4
Hester, 2020‡	57	45.5
Liu, 2014‡	43	57.5
Munoz, 2018‡	39	37.5
Pehlivan, 2019	19	46.4†
Rogers, 2014‡	86	38.2
Svenningsen, 2016	14	42.0
Vodanovich, 2015	85	37.0
Watz, 2019‡	94	41.4
**Total/Mean**	**12695**	**41.3**

Table E4-d. Leicester Cough Questionnaire (LCQ).

**Author 1st, year**	**n**	**LCQ Total**
Polley, 2008	26	14.1
Ozalp, 2012	20	14.7
Murray, 2009b	120	16.9
Munoz, 2013	259	15.1
Altenburg, 2016	30	17.2
Goeminne, 2014	63	15.3
Mandal, 2013	163	17.3
Bilton, 2013	343	15.1
Lee, 2014	85	14.8
Mandal, 2012	27	13.5
Nicolson, 2012	40	13.1
Serisier, 2013b	117	14.9
Alcaraz- Serrano, 2020	64	15.8
Bartley, 2018	32	15.0
Bradley, 2015	55	15.7
Ferri, 2020	119	18.0†

Gao, 2014b	144	12.6
Gao, 2020	333	14.3†
Guan, 2016	148	12.7
Munoz, 2016	259	15.3
Wang, 2018	1188	13.0†
Yildiz, 2018	41	15.5
Spinou, 2017a	54	15.3
Guan, 2014‡	135	12.6
Abdelhalim, 2016‡	30	9.5
Munoz, 2018‡	39	15.1
Ozalp, 2019‡	45	15.1
Rogers, 2014‡	86	14.9
Vodanovich, 2015	85	14.2
Zhong, 2020‡	54	12.3
**Total/Mean**	**4204**	**14.6**

Table E4-e. COPD assessment test (CAT).

**Author 1st, year**	**n**	**CAT Total**
Artaraz, 2020	21	19.1
Brill, 2015	32	16.6
De la Rosa Carrillo, 2020	96	14.8
Lanza, 2018	100	20.0†
Feliu, 2018	53	22.0
Ailiyaer, 2018‡	143	26.1
Sliwinski, 2019‡	18	22.4
Qi, 2019‡	161	19.4
**Total/Mean**	**707**	**20.3**

Table E4-f. Chronic Respiratory Disease Questionnaire (CRDQ).

**Author 1st, year**	**n**	**CRDQ**				
		**Total**	**Dyspnoea**	**Fatigue**	**Emotional function**	**Mastery**
Lee, 2014	85	90.0	NR	NR	NR	NR
Patel, 2019, Patel, 2020	213	79.4	15.6	13.9	30.8	19.1
Vodanovich, 2015	85	82.2	14.3	15.5	32.3	20.3
**Total/Mean**	**383**	**83.9**	**15.0**	**14.7**	**31.6**	**19.7**

Table E4-g. Short Form-36 (SF-36).

Author 1st, year	n	SF-36								
		Total	Physical functioning	Role Physical	Bodily pain	General health	Vitality	Social functioning	Role emotional	Mental health
Machado, 2018	70	NR	50.0	52.5	62.0	40.0	35.0	75.0	66.0	56.0
Bekir, 2017	90	62.01	NR	NR	NR	NR	NR	NR	NR	NR
**Total/Mean**	**160**	**62.01**	**50.0**	**52.5**	**62.0**	**40.0**	**35.0**	**75.0**	**66.0**	**56.0**

Table E4-h. EQ-5D-5L

Author 1st, year	n	EQ-5D-5L Total
Polley, 2008	26	0.70
Dos Reis, 2018	122	0.54§
Yang, 2019, Yang, 2020	52	0.87
Brockwell, 2020‡	204	0.71§
Hester, 2020‡	60	0.73
**Total/Mean**	**464**	**0.71**

Table E4-i. EQ-5D-5L Visual analogue scale (EQ-VAS)

Author 1st, year	n	EQ-VAS Total
Polley, 2008	26	67.4
Quittner, 2015	542	67.2
Zanini, 2015a	108	60
Hester, 2020‡	60	67
**Total/Mean**	**736**	**65.4**

Rows highlighted light grey include prospective studies and dark grey include RCTs reported in original review by Spinou et al. 2016. Rows highlighted white include prospective studies and light blue include RCTs identified in updated search. HRQoL scores not available from the included studies in the systematic review are not listed in this table.
Mean scores unless otherwise stated. † Data are expressed as median. ‡ HRQoL scores for the total population are calculated as the mean of groups/sub-populations at baseline. § Mean scores are from the EQ-5D-3L. # SGRQ symptom score.
*n= 41, [a] n=58, [b] n=43.Resp. S., Respiratory symptoms; Phys. F., Physical functioning; Role F., Role functioning; Health P., Health Perceptions; Emot. F., Emotional functioning; Soc. F., Social functioning; Treat. B., Treatment Burden. NR: Not reported.

For references see main paper and online supplement

Table E5 Studies reporting discriminant validity (p<0.05) of HRQoL questionnaires

HRQoL Questionnaire	Theme	HRQoL Domain	Discriminant validity, p value
Disease Specific			
**QoL-B**	Demographics (i.e. aetiology, age, gender)	Resp. S.	Bronchiectasis with vs. without asthma, p=0.009 (Polverino, 2020)
		Phys. F.	Males vs. females, p=0.046 (McCullough, 2011)
			Males vs. females, p<0.0001 (Finch, 2017); COPD-related vs. other aetiologies, p=0.000 to p=0.03 (Terpstra, 2019)
		Vitality	Males vs. females, p<0.0001 (Finch, 2017)
		Role. F.	NR
		Health P.	COPD-related vs. other aetiologies, p=0.005 to p=0.042 (Terpstra, 2019)
		Emot. F.	Males vs. females, p<0.05 (Olveira, 2014a)
			Males vs. females, p<0.0001 (Finch, 2017)
		Soc. F.	Males vs. females, p=0.007 (Finch, 2017); COPD-related vs. other aetiologies, p= 0.041 to p=0.05 (Terpstra, 2019)
		Treat. B.	Males vs. females, p<0.05 (Olveira, 2014a)
			Males vs. females, p=0.001 (Polverino, 2018)
	Disease severity (e.g. lung function, BSI, CT score)	Resp. S.	FEV_1 <30% vs. 30-49%, 50-80%, >80%, p<0.01 (Olveira, 2014a); FEV_1 <50% vs. 50-80%, >80%, p=0.004 (Quittner, 2015): Bhalla score >13 vs. <13, p<0.01 (Olveira, 2014a);
			Normal vs. obstruction vs. restrictive spirometry pattern, p<0.001 (Visser, 2019); FEV_1%, p<0.05 (Liu, 2019)
		Phys. F.	FEV_1 <30% vs. 30-49%, 50-80%, >80%, p<0.01 (Olveira, 2014a); FEV_1 <50% vs. >50%, p=0.002 (Quittner, 2014); FEV_1 <50% vs. ≥80%, p<0.001 (Quittner, 2015); Bhalla score >13 vs. <13, p<0.01 (Olveira, 2014a);
			Normal vs. obstruction vs. restrictive spirometry pattern, p<0.001 (Visser, 2019); FEV_1%, p<0.05 (Liu, 2019)
		Vitality	Bhalla score >13 vs. <13, p<0.01 (Olveira, 2014a);
			Normal vs. obstruction vs. restrictive spirometry pattern, p=0.01 (Visser, 2019)
		Role F.	FEV_1 <50% vs. >50%, p=0.04 (Quittner, 2014a); FEV_1 <50% vs. 50-80%, >80%, p=0.005 (Quittner, 2015) ; Bhalla score >13 vs. <13, p<0.01 (Olveira, 2014a)
			Normal vs. obstruction vs. restrictive spirometry pattern, p<0.001 (Visser, 2019)
		Health P.	FEV_1 <30% vs. 30-49%, 50-80%, >80%, p<0.01 (Olveira, 2014a); FEV_1 <50% vs. 50-80%, >80%, p=0.030 (Quittner, 2015) ; Bhalla score >13 vs. <13, p<0.01 (Olveira, 2014a)
			Normal vs. obstruction vs. restrictive spirometry pattern, p<0.001 (Visser, 2019); FEV_1%, p<0.05 (Liu, 2019)
		Emot. F.	FEV_1 <30% vs. 30-49%, 50-80%, >80%, p<0.01 (Olveira, 2014a)
			Normal vs. obstruction vs. restrictive spirometry pattern, p=0.049 (Visser, 2019)
		Soc. F.	FEV_1 <30% vs. 30-49%, 50-80%, >80%, p<0.05 (Olveira, 2014a); Bhalla score >13 vs. <13, p<0.01 (Olveira, 2014a)
			Normal vs. obstruction vs. restrictive spirometry pattern, p=0.004 (Visser, 2019)
		Treat. B.	Normal vs. obstruction vs. restrictive spirometry pattern, p<0.001 (Visser, 2019)
	Exacerbations & Hospital Admissions	Resp. S.	Exacerbation vs. convalescence, p<0.05 (Olveira, 2014a)
			Frequent vs. non-frequent exacerbations p<0.01 (Polverino, 2018); p=0.035 (Dhar, 2019); p<0.0001 (Brown, 2019); <3 vs. ≥3 in preceding year, p<0.001 (Visser, 2019)
		Phys. F.	Exacerbation vs. convalescence, p<0.05 (Olveira, 2014a);
			<3 vs. ≥3 exacerbations in preceding year, p<0.001 (Visser, 2019)
		Vitality	Exacerbation vs. convalescence, p<0.05 (Olveira, 2014a);
			<3 vs. ≥3 exacerbations in preceding year, p=0.001 (Visser, 2019); hospitalised vs. non-hospitalised p=0.033 (Alcaraz-Serrano, 2020)
		Role F.	Exacerbation vs. convalescence, p<0.05 (Olveira, 2014a);

		<3 vs. ≥3 exacerbations in preceding year, p<0.001 (Visser, 2019); hospitalised vs. non-hospitalised p=0.001 (Alcaraz-Serrano, 2020)
	Health P.	Exacerbation vs. convalescence, p<0.05 (Olveira, 2014a);
		<3 vs. ≥3 exacerbations in preceding year, p<0.001 (Visser, 2019); hospitalised vs. non-hospitalised p=0.007 (Alcaraz-Serrano, 2020)
	Emot. F.	<3 vs. ≥3 exacerbations in preceding year, p=0.005 (Visser, 2019)
	Soc. F.	Exacerbation vs. convalescence, p<0.05 (Olveira, 2014a);
		<3 vs. ≥3 exacerbations in preceding year, p<0.001 (Visser, 2019)
	Treat. B.	Exacerbation vs. convalescence, p<0.05 (Olveira, 2014a);
		<3 vs. ≥3 exacerbations in preceding year, p=0.001 (Visser, 2019)
Sputum & Bacteriology	Resp. S.	With vs. without *Haemophilus influenzae*, p<0.05, with vs. without *Pa*, p<0.01 (Olveira, 2014a); with vs. without haemoptysis, p<0.01 (Olveira, 2014a);
		Pa vs. *Haemophilus influenzae*, NS (Terpstra, 2019); high vs. moderate, low bacterial load, p=0.01 (Sibila, 2019); with vs. without *Pa*, p<0.01 (Polverino, 2018); with vs. without Enterobacteriaceae, p=0.0014 (Dhar, 2019); high vs. low sputum volume, p<0.01 (Polverino, 2018); p<0.0001 (Dhar, 2019); with vs. without chronic *Pa*, p<0.0001 (Brown, 2019); with vs. without nontuberculous mycobacteria, p=0.014 (Ewen, 2019); p<0.05 (Asakura, 2020)
	Phys. F	With vs. without *Haemophilus influenzae*, p<0.05 (Olveira, 2014a); with vs. without *Pa*, p<0.05 (Olveira, 2014a)
	Vitality	With vs. without haemoptysis, p<0.01 (Olveira, 2014a); with vs. without *Pa*, p<0.05 (Olveira, 2014a)
	Role F	With vs. without haemoptysis, p<0.05 (Olveira, 2014a); with vs. without *Pa*, p<0.05 (Olveira, 2014a);
		With vs. without nontuberculous mycobacteria, p<0.01 (Polverino, 2018)
	Health P.	With vs. without haemoptysis, p<0.05 (Olveira, 2014a); with vs. without *Pa*, p<0.01 (Olveira, 2014a);
		With vs. without nontuberculous mycobacteria, p<0.01 (Polverino, 2018)
	Emot. F.	With vs. without haemoptysis, p<0.05 (Olveira, 2014a);
		With vs. without nontuberculous mycobacteria, p<0.01 (Polverino, 2018)
	Soc. F.	With vs. without haemoptysis, p<0.05 (Olveira, 2014a);
		With vs. without nontuberculous mycobacteria, p<0.01 (Polverino, 2018); p<0.05 (Asakura, 2020)
	Treat. B.	*Pa* vs. *Haemophilus influenzae* or no infection, p=0.03 (Visser, 2019); with vs. without nontuberculous mycobacteria, p<0.01 (Polverino, 2018); *Pa* vs. *Haemophilus influenzae*, NS (Terpstra, 2019)
Signs & Symptoms	Resp. S.	With vs. without haemoptysis, p<0.01 (Olveira, 2014a);
		Severe vs. mild dyspnoea, p<0.0001(Dhar, 2019), p<0.01 (Polverino, 2018)
	Phys. F.	With vs. without haemoptysis, p<0.05 (Olveira, 2014a)
		Severe vs. mild dyspnoea, p<0.001 (De Camargo, 2020)
	Vitality	With vs. without haemoptysis, p<0.01 (Olveira, 2014a)
	Role F.	With vs. without haemoptysis, p<0.05 (Olveira, 2014a)
		Severe vs. mildyspnoea, p=0.014 (De Camargo, 2020)
	Health P.	With vs. without haemoptysis, p<0.05 (Olveira, 2014a)
		Severe vs. mild dyspnoea, p=0.007 (De Camargo, 2020)
	Emot. F.	With vs. without haemoptysis, p<0.05 (Olveira, 2014a)
		Severe vs. mild dyspnoea, p=0.032 (De Camargo, 2020)
	Soc. F.	With vs. without haemoptysis, p<0.05 (Olveira, 2014a)
	Treat. B.	NS (Olveira, 2014a)
Adherence to treatment	Resp. S.	NS (McCullough, 2014, McCullough, 2015)
	Phys. F.	Adherent vs. non-adherent to ACT, p=0.05 (McCullough, 2014); adherent vs. non-adherent to other respiratory medicines, p=0.04 (McCullough, 2015)
	Vitality	NS (McCullough, 2014); NR (McCullough, 2015)
	Role F.	NS (McCullough, 2014); adherent vs. non-adherent to other respiratory medicines, p=0.04 (McCullough, 2015)
	Health. P.	NS (McCullough, 2014); NR (McCullough, 2015)
	Emot. F.	NS (McCullough, 2014); NR (McCullough, 2015)

		Soc. F.	NS (McCullough, 2014); NR (McCullough, 2015)
		Treat. B.	NS (McCullough, 2014, McCullough 2015); adherent vs. non-adherent to inhaled antibiotics p<0.05 (Polverino, 2018); adherent vs. non-adherent to daily chest physiotherapy, p<0.05 (Polverino, 2018)
	Exercise capacity	Resp. S.	6MWT below vs. above median, p<0.001 (Quittner, 2015)
		Phys. F	6MWT below vs. above median, p<0.001 (Quittner, 2015)
		Vitality	6MWT below vs. above median, p<0.001 (Quittner, 2015)
		Role F.	6MWT below vs. above median, p<0.001 (Quittner, 2015)
		Heath P.	6MWT below vs. above median, p=0.009 (Quittner, 2015)
		Emot. F.	6MWT below vs. above median, p=0.024 (Quittner, 2015)
		Soc. F	6MWT below vs. above median, NS (Quittner, 2015)
		Treat. B	6MWT below vs. above median, NS (Quittner, 2015)
**BHQ**	Demographics	Total	Bronchiectasis with vs. without asthma, p<0.001 (Choi, 2020); New Zealand Māori and Pacific peoples vs. other ethnicity (Diggins, 2020)
	Disease severity	Total	FEV_1 <50% vs. >80%, p=0.008 (Spinou, 2017b)
	Exacerbations & Hospital Admissions	Total	With vs. without exacerbations in preceding 12 months, p<0.001 (Spinou, 2017b); with vs. without hospital admissions in preceding 12 months, p<0.001 (Spinou, 2017b)
Respiratory Specific			
**SGRQ**	Demographics	Total	High vs. low BMI, p<0.0001, younger vs. older adults, p=0.008 (Chalmers, 2014);
			Younger adults vs. elderly, p<0.017 (Bellelli, 2016)
		Symptoms	NR
		Activity	NR
		Impact	NR
	Disease Severity	Total	FEV_1 <30% vs. 30-50%, 50-80%, >80%, p<0.0001 (Chalmers, 2014); post bronchodilator-FEV_1 (ml) greater vs. lower severity, p=0.0001 (Martinez-Garcia, 2005); low, intermediate vs. high BSI, p<0.0001 (Chalmers, 2014); greater vs. lowerdisease severity by chest CT, p=0.006 (Martinez-Garcia, 2005), p=0.024 (Eshed, 2007);
			Mild vs. moderate vs. severe BSI, p=0.003 (McDonnell, 2016)
		Symptoms	NR
		Activity	Greater vs. lower disease severity by chest CT, p=0.035 (Eshed, 2007)
		Impact	Greater vs. lower disease severity by chest CT, p=0.022 (Eshed, 2007)
	Exacerbations & Hospital Admissions	Total	Exacerbation vs. convalescence, p<0.001 (Altenburg, 2016), p<0.0001 (Murray, 2009a); frequent vs. non-frequent exacerbations, p<0.0001 (Chalmers, 2014), p=0.002 (Martinez-Garcia, 2005); exacerbation vs. stable, p=0.02, vs. convalescence, p<0.01 (Guan, 2015b); frequent vs. non-frequent exacerbations, p<0.0001 (Chalmers, 2018); hospitalized vs. non-hospitalized exacerbations, p<0.0001 (Chalmers, 2018)
			Frequent vs. non-frequent exacerbations, p<0.0001 (Brown, 2019)
		Symptoms	Exacerbation vs. convalescence, p<0.0001 (Murray, 2009a); 1-2 vs. 3-6 and >6 infections, p≤0.01 (Wilson, 1997a);
			Exacerbation vs. steady state, vs. convalescence, p<0.01 (Guan, 2015b); frequent vs. non-frequent exacerbations, p<0.0001 (Chalmers, 2018)
		Activity	Exacerbation vs. convalescence, p=0.01 (Murray, 2009a);
			Frequent vs. non-frequent exacerbations, p<0.0001(Chalmers, 2018)
		Impact	Exacerbation vs. convalescence, p<0.001 (Murray, 2009a);
			Frequent vs. non-frequent exacerbations, p<0.0001 (Chalmers, 2018)
	Sputum & Bacteriology	Total	With vs. without chronic colonization, p<0.0001, with vs. without *Pa*, p<0.05, *Moraxella catarrhalis*, p<0.05, *Staphylococcus aureus*, p<0.05, MRSA, p<0.05, Gram-negative enterobacteriaceae, p<0.05 (Chalmers, 2014); with vs. without *Pa*, p=0.003 (Martinez-Garcia, 2005); *Pa* vs. other/no infections, p<0.01 infections with other species vs. *Haemophilus influenzae*, p<0.05 and vs. no bacterial growth, p<0.02 (Wilson,1997b); high vs. low sputum volume, p<0.001 (Chan, 2002); p=0.002 (Martinez-Garcia, 2005)

			Isolated or chronic vs. no *Pa*, p<0.001 (Hill, 2015a); chronic *Pa* vs. other chronic infections, daily sputum vs. dry bronchiectasis, p=0.001 (Aliberti, 2016); chronic *Pa* vs. other/no chronic infections, p <0.001 (Araujo, 2018); with vs. without chronic *Pa*, p<0.0001 (Brown, 2019); with vs. without nontuberculous mycobacteria, p<0.001 (Basavaraj, 2019)
		Symptoms	High vs. low sputum volume, p<0.001 (Chan, 2002)
		Activity	*Pa* vs. other/no infections, p<0.01 (Wilson, 1997b); high vs. low sputum volume, p=0.001 (Chan, 2002)
		Impact	*Pa* vs. other/no infections, p<0.01, infections with other species vs. *Haemophilus influenzae*, p<0.05 and vs. no bacterial growth, p<0.02 (Wilson,1997b); high vs. low sputum volume, p<0.001 (Chan, 2002)
	Signs & Symptoms	Total	Severe vs. mild dyspnoea, p<0.0001 (Chalmers, 2014), p≤0.001 (Wilson, 1997a); with vs. without chronic rhinosinusitis, p<0.001 (Guilemany, 2009); Good vs. poor sleepers, p<0.001 (Gao, 2014a)
			High vs. low HADS anxiety score, p=0.004, high vs. low HADS depression score, p<0.0001 (Gao, 2018)
		Symptoms	Severe vs. mild dyspnoea, p≤0.001 (Wilson 1997a); wheeze most days vs. occasional/no wheeze, p<0.001 (Wilson, 2009); good vs. poor sleepers, p=0.003 (Gao, 2014a); with vs. without chronic rhinosinusitis, p<0.001 (Guilemany, 2009);
			High vs. low HADS depression score, p=0.044 (Gao, 2018)
		Activity	Severe vs. mild dyspnoea, p≤0.001 (Wilson, 1997a); good vs. poor sleepers, p=0.002 (Gao, 2014a); with vs. without chronic rhinosinusitis, p<0.001 (Guilemany, 2009);
			High vs. low HADS depression score, p=0.01 (Gao, 2018)
		Impact	Severe vs. mild dyspnoea, p≤0.001 (Wilson, 1997a); good vs. poor sleepers, p<0.001 (Gao, 2014a); with vs. without chronic rhinosinusitis, p<0.001 (Guilemany, 2009);
			High vs. low HADS anxiety score, p<0.0001; high vs. low HADS depression score, p<0.0001 (Gao, 2018)
	Exercise capacity	Total	Low vs. normal 6MWD score, p<0.01 (Guan, 2015a);
			High vs. low improvement in ISWT, p=0.02 (Cartlidge, 2018)
		Symptoms	Low vs. normal 6MWD score, p<0.01 (Guan, 2015a)
		Activity	NR
		Impact	NR
**LCQ**	Demographics	Total	NR
		Physical	NR
		Psychological	NR
		Social	NR
	Disease severity	Total	Severe vs. mild, moderate bronchiectasis, p<0.0001 (Murray 2009b);
			High vs. low BSI, p=0.02 (Guan, 2014), p<0.001 (Munoz, 2016); high vs. low severity by FACED score, p<0.001 (Munoz, 2016); high vs. low severity by HRCT score, p<0.01 (Guan, 2014); long vs. short duration of bronchiectasis symptoms p<0.01 (Guan, 2014)
		Physical	Severe vs. mild, moderate bronchiectasis, p<0.0001 (Murray, 2009b)
		Psychological	Severe vs. mild, moderate bronchiectasis, p<0.0001 (Murray, 2009b)
		Social	Severe vs. mild, moderate bronchiectasis, p<0.0001 (Murray, 2009b)
	Exacerbations & Hospital Admissions	Total	Exacerbation vs. convalescence, p=0.001 (Altenburg, 2016), p<0.0001 (Murray, 2009b);
			Stable vs. exacerbation, p<0.001 (Munoz, 2016)
		Physical	Exacerbation vs. convalescence, p<0.0001 (Murray, 2009b);
			Stable vs. exacerbation, p<0.001 (Munoz, 2016)
		Psychological	Exacerbation vs. convalescence, p<0.0001 (Murray, 2009b);
			Stable vs. exacerbation, p<0.001 (Munoz, 2016)
		Social	Exacerbation vs. convalescence, p<0.0001 (Murray, 2009b);
			Stable vs. exacerbation, p<0.001 (Munoz, 2016)
	Sputum & Bacteriology	Total	Commensals vs. other potentially pathogenic microorganisms vs. *Pa*, p=0.03 (Guan, 2014); with vs. without *Pa*, p<0.001 (Wang, 2018); chronic *Pa* vs. other chronic infections, daily sputum vs. dry bronchiectasis, p=0.004 (Aliberti, 2016); high vs. low sputum volume p<0.01, high vs. low sputum purulence p<0.01 (Guan, 2014)
		Physical	NR
		Psychological	NR

		Social	NR
	Signs & Symptoms	Total	With vs. without airway reflux, p<0.001) (Mandal, 2013);<br>Long vs. short duration of symptoms p<0.01, with vs. without capsaicin cough hypersensitivity p=0.03 (Guan, 2014)
		Physical	NR
		Psychological	NR
**CAT**	Disease severity	Total	Mild vs. moderate e-FACED, p=0.002; mild vs. severe BSI, p=0.05 (De la Rosa Carrillo, 2020); low vs. high e-FACED, p<0.05 (Lanza, 2018)
	Exacerbations & Hospital Admissions	Total	Frequent vs. non-frequent exacerbations, p=0.0054 (Finch, 2020)
	Sputum & Bacteriology	Total	With vs. without potentially pathogenic microorganisms, p=0.001 (Feliu, 2018)
	Signs & Symptoms	Total	Severe vs. mild dyspnoea, p<0.05 (Lanza, 2018)
	Exercise capacity	Total	High vs. low walk distance, VO_2, workload, number of steps, p<0.05 (Lanza, 2018)
**CRDQ**	Exacerbations & Hospital Admissions	Total	NR
		Dyspnoea	Exacerbation vs. convalescence, p=0.004 (Courtney, 2008)
		Fatigue	NS (Courtney, 2008)
		Emotional function	Exacerbation vs. convalescence, p=0.004 (Courtney, 2008)
		Mastery	Exacerbation vs. convalescence, p=0.04 (Courtney, 2008)
**SNOT-20**	Signs & Symptoms	Total	With vs. without chronic rhinosinusitis, p<0.001 (Guilemany, 2009)
		Total	With vs. without chronic rhinosinusitis, p<0.001 (Guilemany, 2009)
**SOLQ**	Signs & Symptoms	Phys. F.	With vs. without bronchial hyperreactivity, p=0.01 (Bulcun, 2015)
		Emot. F.	NS (Bulcun, 2015)
		Coping Skills	With vs. without bronchial hyperreactivity, p=0.01 (Bulcun, 2015)
		Treatment Satisfaction	NS (Bulcun, 2015)
Generic			
**SF-36**	Demographics	Total	NR
		Phys. F.	Bronchiectasis vs. age and sex matched healthy controls, p=0.001 (Bulcun, 2015)
		Role physical	NR
		Bodily pain	NR
		General health	NR
		Vitality	NR
		Soc. F.	NR
		Role emotional	NR
		Mental health	Bronchiectasis vs. age and sex matched healthy controls, p=0.005 (Bulcun, 2015)
	Disease severity	Total	Mild vs. moderate-high FACED, p= 0.001 (Bekir, 2017)
		Phys. F.	NR
		Role physical	NR
		Bodily pain	NR
		General health	NR
		Vitality	NR
		Soc. F.	NR
		Role emotional	NR
		Mental health	NR
	Exacerbations & Hospital Admissions	Total	Exacerbation vs. convalescence, p=0.002 (Altenburg, 2016)
		Phys. F.	NR
		Role physical	NR
		Bodily pain	NR
		General health	NR

		Vitality	NR
		Soc. F.	NR
		Role emotional	NR
		Mental health	NR
	Sputum & Bacteriology	Total	NR
		Phys. F.	*Pa* vs. other infections, p<0.05, other species vs. *Haemophilus influenzae*, p<0.01 (Wilson, 1997)
		Role physical	NR
		Bodily pain	High vs. low sputum volume, p=0.006 (Chan, 2002)
		General health	High vs. low sputum volume, p=0.001 (Chan, 2002)
		Vitality	NR
		Soc. F.	NR
		Role emotional	NR
		Mental health	NR
	Signs & Symptoms	Total	With vs. without depression, p=0.012 (Bekir, 2017)
		Phys. F.	With vs. without chronic rhinosinusitis, p<0.05 (Guilemany, 2009)
			With vs. without bronchial hyperreactivity, p=0.008 (Bulcun, 2015)
		Role physical	NR
		Bodily pain	NR
		General health	NR
		Vitality	NR
		Soc. F.	NR
		Role emotional	NR
		Mental health	With vs. without chronic rhinosinusitis, p<0.05 (Guilemany, 2009)
			With vs. without bronchial hyperreactivity, p=0.02 (Bulcun, 2015)
**EQ-5D-5L**	Demographics	VAS component	Males vs. females after pulmonary rehabilitation, p=0.043 (Zanini, 2015a)
		Index	With vs. without bronchiectasis, p<0.001 (Yang, 2020)
		Mobility	With vs. without bronchiectasis, p=0.04 (Yang, 2020)
		Self-care	NS (Yang, 2020)
		Usual activity	With vs. without bronchiectasis, p<0.001 (Yang, 2020)
		Pain/discomfort	With vs. without bronchiectasis, p=0.001 (Yang, 2020)
		Anxiety/depression	With vs. without bronchiectasis, p=0.004 (Yang, 2020)
	Disease severity	VAS component	With vs. without airflow obstruction, p=0.011 (Zanini, 2015a)
		Index	NR
		Mobility	NR
		Self-care	NR
		Usual activity	NR
		Pain/discomfort	NR
		Anxiety/depression	NR

Rows highlighted grey include studies reported in prior review by Spinou et al. (2016). Rows highlighted white include studies identified in updated search.
ACT, airway clearance therapy; BHQ, Bronchiectasis Health Questionnaire; BMI, body mass index; BSI, Bronchiectasis Severity Index; COPD, Chronic Obstructive Pulmonary Disease; CAT, COPD Assessment Tool; Emot. F., Emotional functioning; EQ-5D-5L (VAS), EuroQoL 5-level (Visual Analogue Scale) FACED, an acronym for FEV_1, age, chronic colonisation, extension and dyspnoea; e-FACED, FACED plus exacerbations; FEV_1, forced expiratory volume in one second; HADS, Hospital Anxiety and Depression Scale; Health P., Health perceptions; HRCT, high-resolution computed tomography; HRQoL, health-related quality of life; ISWT, increment shuttle walk test; LCQ, Leicester Cough Questionnaire; MRSA, methicillin-resistant Staphylococcus aureus; NR, not reported; NS, not significant; Phys. F., Physical functioning; *Pa, Pseudomonas aeruginosa*; p, p value; QoL-B, Quality of Life-Bronchiectasis Questionnaire; Resp. S, Respiratory symptoms; Role F., Role functioning; SF-36, Medical Outcomes Study 36-item Short-Form Health Survey; SGRQ, St George's Respiratory Questionnaire; SNOT-20, Sino-Nasal Outcome Test-20; Soc. F., Social functioning; SOLQ, Seattle Obstructive Lung Disease; Treat. B., Treatment burden; VO_2, maximal oxygen uptake; vs, versus; 6MWD, six-minute walking distance

For references see main paper and online supplement.

Table E6 Associations between health-related quality of life questionnaires

Author 1st, year	HRQoL questionnaire 1	HRQoL questionnaire 2	Domain	r-value	p-value
Quittner, 2014	QoL-B V2.0	SGRQ	Symptoms		
	Resp. S.			-0.73	<0.001
	Phys .F.			-0.68	<0.001
	Vitality			-0.58	<0.001
	Role. F.			NR	NR
	Health. P.			NR	NR
	Emot. F.			-0.38	<0.001
	Soc. F.			-0.64	<0.001
	Treat. B.			NR	NR
	QoL-B V2.0	SGRQ	Activity		
	Resp. S.			-0.53	<0.001
	Phys. F.			-0.85	<0.001
	Vitality			-0.64	<0.001
	Role. F.			NR	NR
	Health. P.			NR	NR
	Soc. F.			-0.48	<0.001
	Emot. F.			-0.41	<0.001
	Treat. B.			NR	NR
	QoL-B V2.0	SGRQ	Impact		
	Resp. S.			-0.62	<0.001
	Phys. F.			-0.76	<0.001
	Vitality			-0.68	<0.001
	Role. F.			NR	NR
	Health. P.			NR	NR
	Emot. F.			-0.54	<0.001
	Soc. F.			-0.69	<0.001
	Treat. B.			NR	NR
Quittner, 2015 AIR-BX1	QoL-B V3.0	EQ-5D-5L	EQ-5D VAS		
	Resp. S.			0.512	<0.001
	Phys. F.			0.568	<0.001
	Vitality			0.546	<0.001
	Role. F.			0.621	<0.001
	Health. P.			0.294	<0.001
	Soc. F.			0.376	<0.001
	Emot. F.			NR	NR
	Treat. B.			0.463	<0.001
Quittner, 2015 AIR-BX2	QoL-B V3.0	EQ-5D-5L	EQ-5D VAS		
	Resp. S.			0.519	<0.001
	Phys. F.			0.633	<0.001
	Vitality			0.631	<0.001
	Role F.			0.663	<0.001
	Health. P.			0.640	<0.001

	Soc. F.			0.378	<0.001
	Emot. F.			0.432	<0.001
	Treat. B			0.331	<0.001
Olveira, 2014a	QoL-B-Sp-V3.0	SGRQ	Total		
	Resp. S			-0.69	<0.01
	Phys. F.			-0.81	<0.01
	Vitality			-0.67	<0.01
	Role. F.			-0.77	<0.01
	Health. P.			-0.68	<0.01
	Social. F.			-0.53	<0.01
	Emot. F			-0.64	<0.01
	Treat. B			-0.34	<0.01
Brown, 2019	QOL-B	SGRQ	Total		
	Resp. S			-0.74	<0.0001
	Phys. F.			NR	NR
	Vitality			NR	NR
	Role. F.			NR	NR
	Health. P.			NR	NR
	Social. F.			NR	NR
	Emot. F			NR	NR
	Treat. B			NR	NR
De Camargo, 2020	QOL-B Brazilian Portuguese	SGRQ	Symptoms		
	Resp. S			-0.46	<0.01
	Phys. F.			-0.32	<0.01
	Vitality			-0.38	<0.01
	Role. F.			-0.26	<0.01
	Health. P.			-0.30	<0.01
	Social. F.			-0.31	<0.01
	Emot. F			-0.26	<0.01
	Treat. B			-0.08	NR
	QOL-B Brazilian Portuguese	SGRQ	Activity		
	Resp. S			-0.40	<0.01
	Phys. F.			-0.7	<0.01
	Vitality			-0.40	<0.01
	Role. F.			-0.31	<0.01
	Health. P.			-0.62	<0.01
	Social. F.			-0.35	<0.01
	Emot. F			-0.34	<0.01
	Treat. B			0.05	NR
	QOL-B Brazilian Portuguese	SGRQ	Impact		
	Resp. S			-0.54	<0.01
	Phys. F.			-0.65	<0.01
	Vitality			-0.38	<0.01
	Role. F.			-0.43	<0.01
	Health. P.			-0.51	<0.01

	Social. F.			-0.54	<0.01
	Emot. F			-0.47	<0.01
	Treat. B			0.07	NR
	QOL-B Brazilian Portuguese	SGRQ	Total		
	Resp. S			-0.54	<0.01
	Phys. F.			-0.69	<0.01
	Vitality			-0.44	<0.01
	Role. F.			-0.4	<0.01
	Health. P.			-0.55	<0.01
	Social. F.			-0.48	<0.01
	Emot. F			-0.45	<0.01
	Treat. B			0.01	NR
Liu, 2019	QoL-B	SGRQ	Total	-0.704	<0.01
	Resp. S			NR	NR
	Phys. F.			NR	NR
	Vitality			NR	NR
	Role. F.			NR	NR
	Health. P.			NR	NR
	Social. F.			NR	NR
	Emot. F			NR	NR
	Treat. B			NR	NR
Gissel, 2020	BHQ- Danish	SGRQ	Total	-0.826	0.0001
			Symptoms	NR	NR
			Activity	NR	NR
			Impact	NR	NR
Kim, 2020	BHQ- Korean	CAT- Korean	Total	-0.656	<0.001
Wilson, 1997a	SGRQ Total	SF-36	Physical	-0.75	<0.0001
			Mental	-0.35	<0.001
	SGRQ Symptoms	SF-36	Physical	-0.58	<0.0001
	SGRQ Activity	SF-36	Physical	-0.70	<0.0001
	SGRQ Impact	SF-36	Physical	-0.68	<0.0001
Chan, 2002	SGRQ-HK Total	SF-36	Physical	-0.68	<0.001
	SGRQ-HK Symptoms	SF-36	Physical	-0.33	<0.001
	SGRQ-HK Activity	SF-36	Physical	-0.78	<0.001
	SGRQ-HK Impact	SF-36	Physical	-0.59	<0.001
Lee, 2010	LCQ-Total	SGRQ	Total	-0.70	<0.01
		CRDQ	Total	0.51	<0.01
		CQLQ	Total	-0.88	<0.001
Polley, 2008	LCQ-Total	EQ-5D-5L	EQ-5D index value	0.67	<0.001
			EQ-5D VAS	0.52	0.01
Gao, 2014b	LCQ-MC Total	SGRQ	Total	-0.600	<0.001
			Symptoms	-0.493	<0.001
			Activity	-0.388	<0.001
			Impact	-0.627	<0.001
	LCQ-MC Physical	SGRQ	Total	-0.564	<0.001
			Symptoms	-0.474	<0.001

			Activity	-0.367	<0.001
			Impact	-0.590	<0.001
	LCQ-MC Psychological	SGRQ	Total	-0.535	<0.001
			Symptoms	-0.446	<0.001
			Activity	-0.295	<0.001
			Impact	-0.587	<0.001
	LCQ-MC Social	SGRQ	Total	-0.589	<0.001
			Symptoms	-0.498	<0.001
			Activity	-0.428	<0.001
			Impact	-0.585	<0.001
Munoz, 2016	LCQ-Sp Total	SGRQ	Total	-0.66	<0.01
			Symptoms	-0.52	<0.01
			Activity	-0.53	<0.01
			Impact	-0.68	<0.01
	LCQ-Sp Physical	SGRQ	Total	-0.67	<0.01
			Symptoms	-0.55	<0.01
			Activity	-0.54	<0.01
			Impact	-0.65	<0.01
	LCQ-Sp Psychological	SGRQ	Total	-0.59	<0.01
			Symptoms	-0.46	<0.01
			Activity	-0.45	<0.01
			Impact	-0.64	<0.01
	LCQ-Sp Social	SGRQ	Total	-0.65	<0.01
			Symptoms	-0.52	<0.01
			Activity	-0.53	<0.01
			Impact	-0.68	<0.01
Brill, 2015	CAT	SGRQ	Total	0.799	<0.001
Lanza, 2018	CAT	SGRQ	Total	0.74	<0.001
			Symptoms	0.61	<0.001
			Activity	0.61	<0.001
			Impact	0.72	<0.001
De la Rosa Carrillo, 2020	CAT	QoL-B	Resp. S.	-0.68	<0.001
			Phys. F.	-0.68	<0.001
			Soc. F.	-0.45	<0.001
			Vitality	-0.15	NS
			Role. F.	-0.17	NS
			Health. P.	0.52	<0.001
			Emot. F.	-0.44	<0.001
			Treat. B.	0.34	<0.001
		BHQ	Total	-0.70	<0.001
		SGRQ	Total	0.75	<0.001
		SGRQ	Symptoms	0.59	<0.001
		SGRQ	Activity	0.65	<0.001
		SGRQ	Impact	0.67	<0.001
Finch, 2020	CAT	SGRQ[1]	Total	0.90	< 0.0001

		SGRQ[2]	Total	0.87	< 0.0001
		SGRQ[2]	Symptoms	0.68	< 0.0001
		SGRQ[2]	Activity	0.84	< 0.0001
		SGRQ[2]	Impact	0.83	< 0.0001
		QOL-B[2]	Resp. S.	-0.75	< 0.0001
			Phys. F.	NR	NR
			Soc. F.	NR	NR
			Vitality	NR	NR
			Role. F.	NR	NR
			Health. P.	NR	NR
			Emot. F.	NR	NR
			Treat. B.	NR	NR
		LCQ[2]	Total	-0.77	< 0.0001
			Physical	NR	NR
			Psychological	NR	NR
			Social	NR	NR
Vodanovich, 2015	CRDQ Total	SGRQ	Total	-0.493	<0.01
			Symptoms	-0.367	<0.05
			Activity	-0.346	<0.05
			Impact	-0.482	<0.01
	CRDQ Dyspnoea		Total	0.28	NS
			Symptoms	0.38	<0.05
			Activity	0.215	NS
			Impact	0.26	NS
	CRDQ Fatigue		Total	-0.541	<0.01
			Symptoms	-0.319	<0.05
			Activity	-0.63	<0.01
			Impact	-0.383	<0.05
	CRDQ Emotional		Total	-0.471	<0.01
			Symptoms	-0.342	<0.05
			Activity	-0.306	<0.05
			Impact	-0.521	<0.01
	CRDQ Mastery		Total	-0.685	<0.01
			Symptoms	-0.559	<0.01
			Activity	-0.463	<0.01
			Impact	-0.684	<0.01
	CRDQ Total	LCQ	Total	0.512	<0.01
			Physical	0.571	<0.01
			Psychological	0.467	<0.01
			Social	0.153	NS
	CRDQ Dyspnoea		Total	0.00	NS
			Physical	0.06	NS
			Psychological	-0.04	NS
			Social	0.098	NS
	CRDQ Fatigue		Total	0.406	<0.01
			Physical	0.551	<0.01

			Psychological	0.334	<0.05
			Social	0.177	NS
	CRDQ Emotional		Total	0.561	<0.01
			Physical	0.53	<0.05
			Psychological	0.559	<0.01
			Social	0.170	NS
	CRDQ Mastery		Total	0.619	<0.01
			Physical	0.669	<0.01
			Psychological	0.556	<0.01
			Social	0.188	NS
Bulcun, 2015	SOLQ Phys. F.	SF-36	Physical	0.53	<0.001
			Mental	0.52	<0.001
	SOLQ Emot. F.	SF-36	Physical	0.55	<0.001
			Mental	0.53	<0.001
	SOLQ Coping Skills	SF-36	Physical	0.47	<0.001
			Mental	0.46	<0.001
	SOLQ Treatment Satisfaction	SF-36	Physical	0.40	<0.001
			Mental	0.50	<0.001

Rows highlighted grey include values reported in original review by Spinou et al. 2016. Rows highlighted white include values reported in studies identified in updated search.

[1]Data from the TRIBE cohort, [2]Data from the validation cohort.

BHQ, Bronchiectasis Health Questionnaire; CRDQ, Chronic Respiratory Disease Questionnaire; Emot. F., Emotional functioning; EQ-VAS, EuroQoL EQ-5D (Visual Analogue Scale);CAT, COPD Assessment Test; Health P., Health perceptions; HK-Hong-Kong; LCQ; Leicester Cough Questionnaire; MC, Mandarin Chinese; Phys. F., Physical functioning; QOL-B, Quality of life-Bronchiectasis; Resp. S, Respiratory symptoms; Role F., Role functioning; SGRQ, St. Georges Respiratory Questionnaire; SF-36, 36-Item Short Form Survey; Soc. F., Social functioning; SOLQ, Seattle Obstructive Lung Disease Questionnaire; Sp, Spanish; Treat. B., Treatment burden; NR, not reported.

For references see main paper and online supplement.

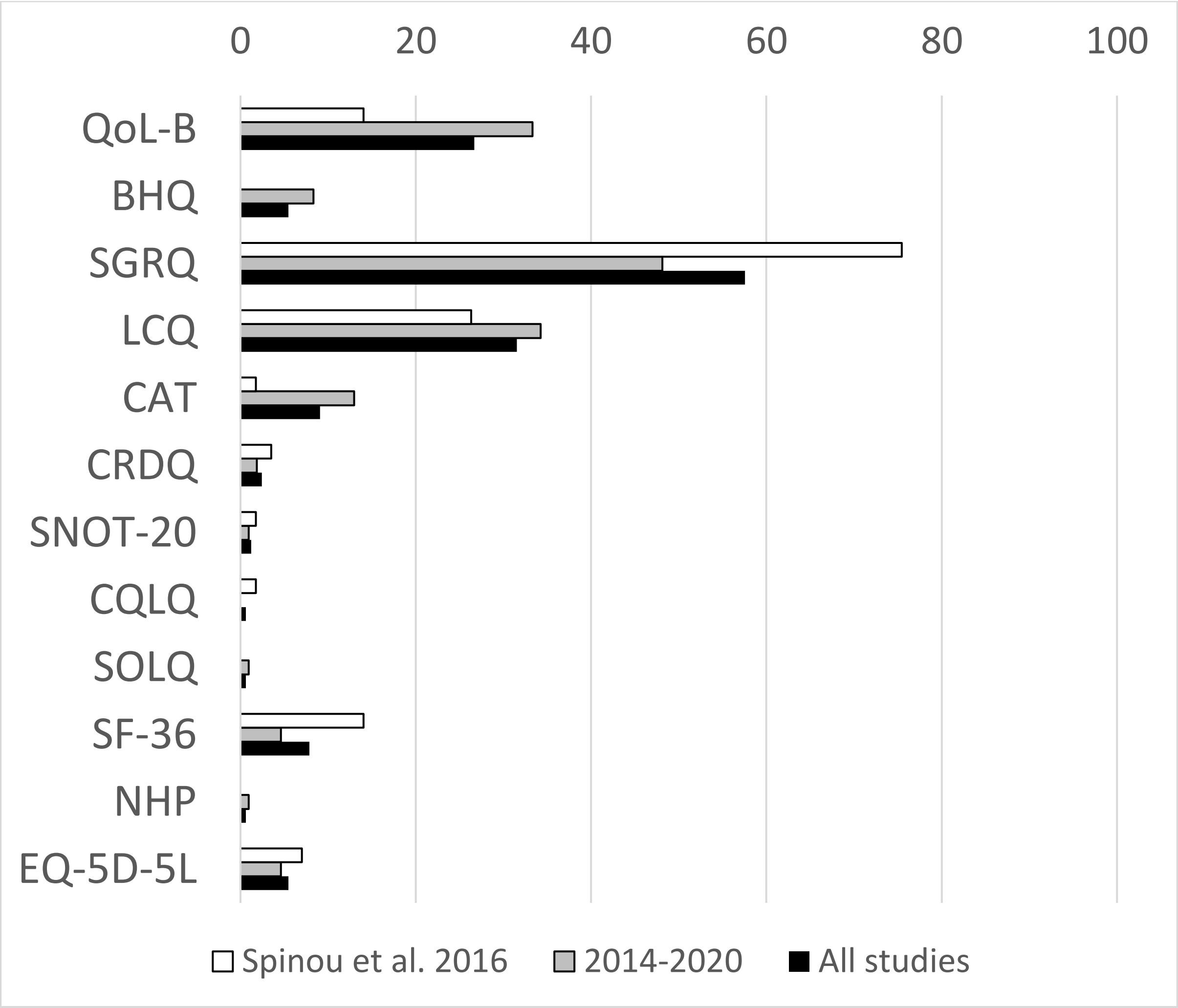
0
20
40
60
80
100
QoL-B
BHQ
SGRQ
LCQ
CAT
CRDQ
SNOT-20
CQLQ
SOLQ
SF-36
NHP
EQ-5D-5L
Spinou et al. 2016
2014-2020
All studies

Figure E1

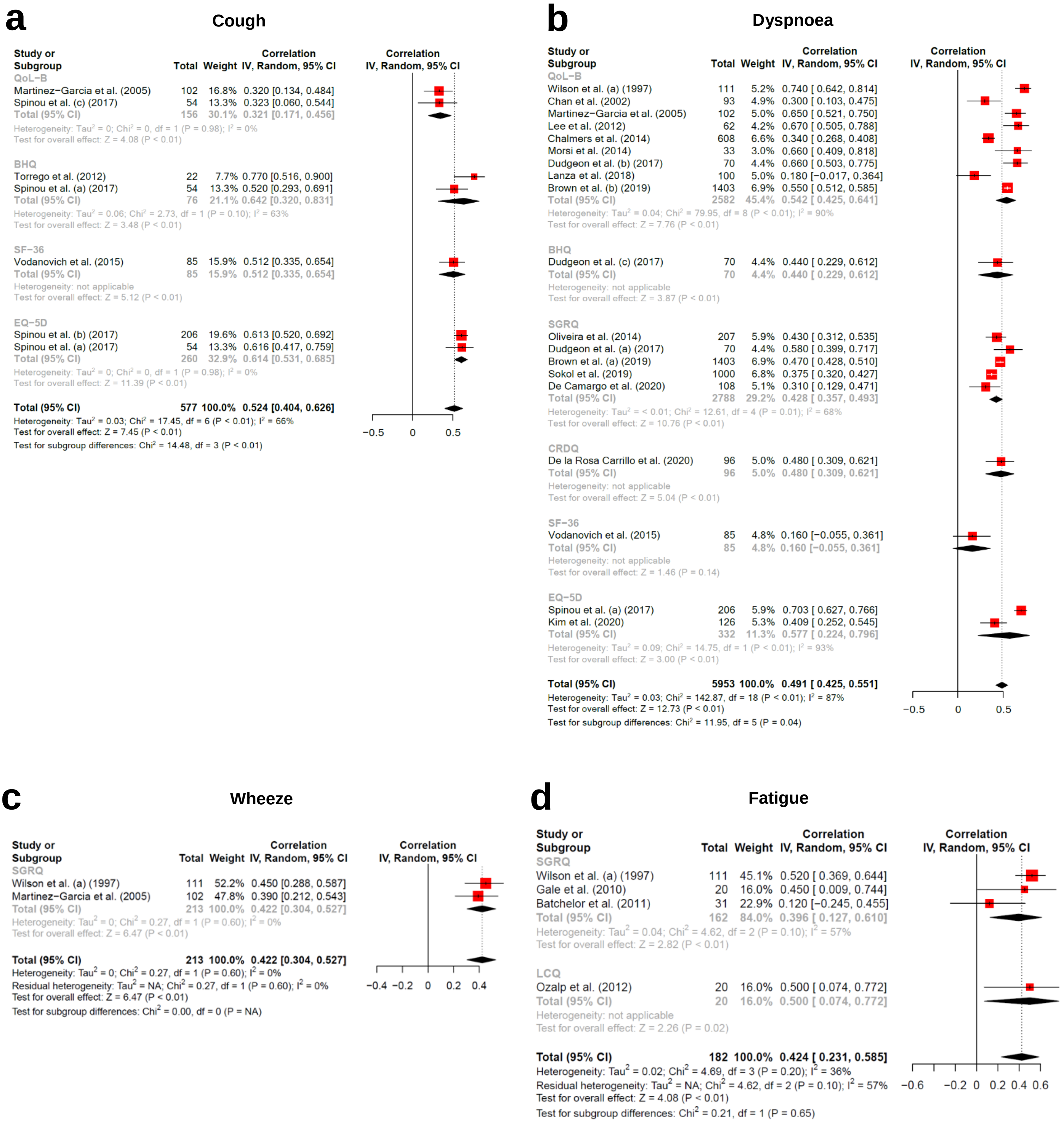
a
Cough
Study or Subgroup | Total | Weight | Correlation IV, Random, 95% CI
QoL−B
Martinez−Garcia et al. (2005) 102 16.8% 0.320 [0.134, 0.484]
Spinou et al. (c) (2017) 54 13.3% 0.323 [0.060, 0.544]
Total (95% CI) 156 30.1% 0.321 [0.171, 0.456]
Heterogeneity: Tau^2 = 0; Chi^2 = 0, df = 1 (P = 0.98); I^2 = 0%
Test for overall effect: Z = 4.08 (P < 0.01)
BHQ
Torrego et al. (2012) 22 7.7% 0.770 [0.516, 0.900]
Spinou et al. (a) (2017) 54 13.3% 0.520 [0.293, 0.691]
Total (95% CI) 76 21.1% 0.642 [0.320, 0.831]
Heterogeneity: Tau^2 = 0.06; Chi^2 = 2.73, df = 1 (P = 0.10); I^2 = 63%
Test for overall effect: Z = 3.48 (P < 0.01)
SF−36
Vodanovich et al. (2015) 85 15.9% 0.512 [0.335, 0.654]
Total (95% CI) 85 15.9% 0.512 [0.335, 0.654]
Heterogeneity: not applicable
Test for overall effect: Z = 5.12 (P < 0.01)
EQ−5D
Spinou et al. (b) (2017) 206 19.6% 0.613 [0.520, 0.692]
Spinou et al. (a) (2017) 54 13.3% 0.616 [0.417, 0.759]
Total (95% CI) 260 32.9% 0.614 [0.531, 0.685]
Heterogeneity: Tau^2 = 0; Chi^2 = 0, df = 1 (P = 0.98); I^2 = 0%
Test for overall effect: Z = 11.39 (P < 0.01)
Total (95% CI) 577 100.0% 0.524 [0.404, 0.626]
Heterogeneity: Tau^2 = 0.03; Chi^2 = 17.45, df = 6 (P < 0.01); I^2 = 66%
Test for overall effect: Z = 7.45 (P < 0.01)
Test for subgroup differences: Chi^2 = 14.48, df = 3 (P < 0.01)
−0.5 0 0.5
b
Dyspnoea
Study or Subgroup | Total | Weight | Correlation IV, Random, 95% CI
QoL−B
Wilson et al. (a) (1997) 111 5.2% 0.740 [0.642, 0.814]
Chan et al. (2002) 93 4.9% 0.300 [0.103, 0.475]
Martinez−Garcia et al. (2005) 102 5.0% 0.650 [0.521, 0.750]
Lee et al. (2012) 62 4.2% 0.670 [0.505, 0.788]
Chalmers et al. (2014) 608 6.6% 0.340 [0.268, 0.408]
Morsi et al. (2014) 33 3.0% 0.660 [0.409, 0.818]
Dudgeon et al. (b) (2017) 70 4.4% 0.660 [0.503, 0.775]
Lanza et al. (2018) 100 5.0% 0.180 [−0.017, 0.364]
Brown et al. (b) (2019) 1403 6.9% 0.550 [0.512, 0.585]
Total (95% CI) 2582 45.4% 0.542 [0.425, 0.641]
Heterogeneity: Tau^2 = 0.04; Chi^2 = 79.95, df = 8 (P < 0.01); I^2 = 90%
Test for overall effect: Z = 7.76 (P < 0.01)
BHQ
Dudgeon et al. (c) (2017) 70 4.4% 0.440 [0.229, 0.612]
Total (95% CI) 70 4.4% 0.440 [0.229, 0.612]
Heterogeneity: not applicable
Test for overall effect: Z = 3.87 (P < 0.01)
SGRQ
Oliveira et al. (2014) 207 5.9% 0.430 [0.312, 0.535]
Dudgeon et al. (a) (2017) 70 4.4% 0.580 [0.399, 0.717]
Brown et al. (a) (2019) 1403 6.9% 0.470 [0.428, 0.510]
Sokol et al. (2019) 1000 6.8% 0.375 [0.320, 0.427]
De Camargo et al. (2020) 108 5.1% 0.310 [0.129, 0.471]
Total (95% CI) 2788 29.2% 0.428 [0.357, 0.493]
Heterogeneity: Tau^2 = < 0.01; Chi^2 = 12.61, df = 4 (P = 0.01); I^2 = 68%
Test for overall effect: Z = 10.76 (P < 0.01)
CRDQ
De la Rosa Carrillo et al. (2020) 96 5.0% 0.480 [0.309, 0.621]
Total (95% CI) 96 5.0% 0.480 [0.309, 0.621]
Heterogeneity: not applicable
Test for overall effect: Z = 5.04 (P < 0.01)
SF−36
Vodanovich et al. (2015) 85 4.8% 0.160 [−0.055, 0.361]
Total (95% CI) 85 4.8% 0.160 [−0.055, 0.361]
Heterogeneity: not applicable
Test for overall effect: Z = 1.46 (P = 0.14)
EQ−5D
Spinou et al. (a) (2017) 206 5.9% 0.703 [0.627, 0.766]
Kim et al. (2020) 126 5.3% 0.409 [0.252, 0.545]
Total (95% CI) 332 11.3% 0.577 [0.224, 0.796]
Heterogeneity: Tau^2 = 0.09; Chi^2 = 14.75, df = 1 (P < 0.01); I^2 = 93%
Test for overall effect: Z = 3.00 (P < 0.01)
Total (95% CI) 5953 100.0% 0.491 [0.425, 0.551]
Heterogeneity: Tau^2 = 0.03; Chi^2 = 142.87, df = 18 (P < 0.01); I^2 = 87%
Test for overall effect: Z = 12.73 (P < 0.01)
Test for subgroup differences: Chi^2 = 11.95, df = 5 (P = 0.04)
−0.5 0 0.5
c
Wheeze
Study or Subgroup | Total | Weight | Correlation IV, Random, 95% CI
SGRQ
Wilson et al. (a) (1997) 111 52.2% 0.450 [0.288, 0.587]
Martinez−Garcia et al. (2005) 102 47.8% 0.390 [0.212, 0.543]
Total (95% CI) 213 100.0% 0.422 [0.304, 0.527]
Heterogeneity: Tau^2 = 0; Chi^2 = 0.27, df = 1 (P = 0.60); I^2 = 0%
Test for overall effect: Z = 6.47 (P < 0.01)
Total (95% CI) 213 100.0% 0.422 [0.304, 0.527]
Heterogeneity: Tau^2 = 0; Chi^2 = 0.27, df = 1 (P = 0.60); I^2 = 0%
Residual heterogeneity: Tau^2 = NA; Chi^2 = 0.27, df = 1 (P = 0.60); I^2 = 0%
Test for overall effect: Z = 6.47 (P < 0.01)
Test for subgroup differences: Chi^2 = 0.00, df = 0 (P = NA)
−0.4 −0.2 0 0.2 0.4
d
Fatigue
Study or Subgroup | Total | Weight | Correlation IV, Random, 95% CI
SGRQ
Wilson et al. (a) (1997) 111 45.1% 0.520 [0.369, 0.644]
Gale et al. (2010) 20 16.0% 0.450 [0.009, 0.744]
Batchelor et al. (2011) 31 22.9% 0.120 [−0.245, 0.455]
Total (95% CI) 162 84.0% 0.396 [0.127, 0.610]
Heterogeneity: Tau^2 = 0.04; Chi^2 = 4.62, df = 2 (P = 0.10); I^2 = 57%
Test for overall effect: Z = 2.82 (P < 0.01)
LCQ
Ozalp et al. (2012) 20 16.0% 0.500 [0.074, 0.772]
Total (95% CI) 20 16.0% 0.500 [0.074, 0.772]
Heterogeneity: not applicable
Test for overall effect: Z = 2.26 (P = 0.02)
Total (95% CI) 182 100.0% 0.424 [0.231, 0.585]
Heterogeneity: Tau^2 = 0.02; Chi^2 = 4.69, df = 3 (P = 0.20); I^2 = 36%
Residual heterogeneity: Tau^2 = NA; Chi^2 = 4.62, df = 2 (P = 0.10); I^2 = 57%
Test for overall effect: Z = 4.08 (P < 0.01)
Test for subgroup differences: Chi^2 = 0.21, df = 1 (P = 0.65)
−0.6 −0.2 0 0.2 0.4 0.6

Figure E2

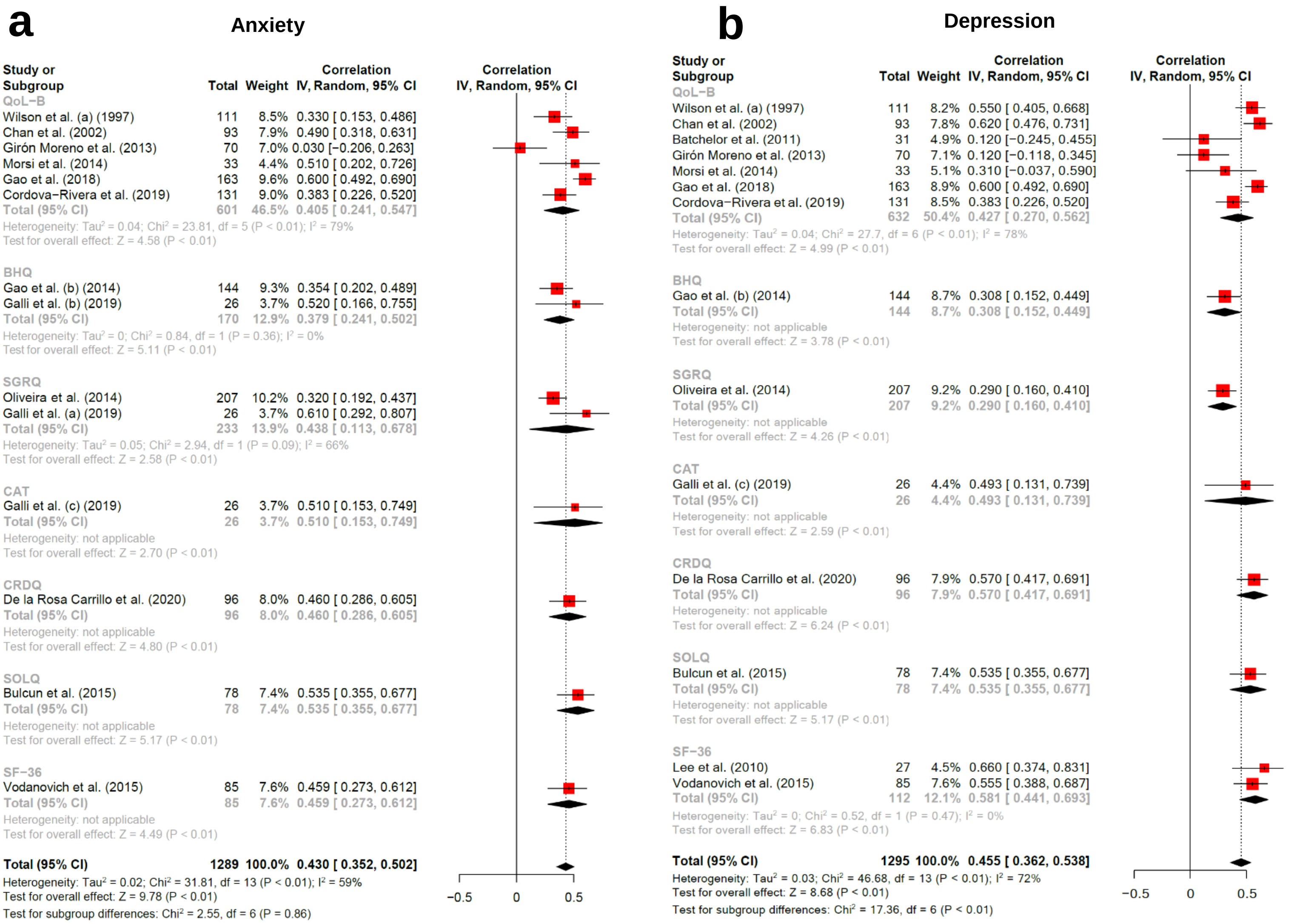
a
Anxiety
Study or Subgroup
Total
Weight
Correlation IV, Random, 95% CI
Correlation IV, Random, 95% CI
QoL−B
Wilson et al. (a) (1997) 111 8.5% 0.330 [0.153, 0.486]
Chan et al. (2002) 93 7.9% 0.490 [0.318, 0.631]
Girón Moreno et al. (2013) 70 7.0% 0.030 [−0.206, 0.263]
Morsi et al. (2014) 33 4.4% 0.510 [0.202, 0.726]
Gao et al. (2018) 163 9.6% 0.600 [0.492, 0.690]
Cordova−Rivera et al. (2019) 131 9.0% 0.383 [0.226, 0.520]
Total (95% CI) 601 46.5% 0.405 [0.241, 0.547]
Heterogeneity: Tau^2 = 0.04; Chi^2 = 23.81, df = 5 (P < 0.01); I^2 = 79%
Test for overall effect: Z = 4.58 (P < 0.01)
BHQ
Gao et al. (b) (2014) 144 9.3% 0.354 [0.202, 0.489]
Galli et al. (b) (2019) 26 3.7% 0.520 [0.166, 0.755]
Total (95% CI) 170 12.9% 0.379 [0.241, 0.502]
Heterogeneity: Tau^2 = 0; Chi^2 = 0.84, df = 1 (P = 0.36); I^2 = 0%
Test for overall effect: Z = 5.11 (P < 0.01)
SGRQ
Oliveira et al. (2014) 207 10.2% 0.320 [0.192, 0.437]
Galli et al. (a) (2019) 26 3.7% 0.610 [0.292, 0.807]
Total (95% CI) 233 13.9% 0.438 [0.113, 0.678]
Heterogeneity: Tau^2 = 0.05; Chi^2 = 2.94, df = 1 (P = 0.09); I^2 = 66%
Test for overall effect: Z = 2.58 (P < 0.01)
CAT
Galli et al. (c) (2019) 26 3.7% 0.510 [0.153, 0.749]
Total (95% CI) 26 3.7% 0.510 [0.153, 0.749]
Heterogeneity: not applicable
Test for overall effect: Z = 2.70 (P < 0.01)
CRDQ
De la Rosa Carrillo et al. (2020) 96 8.0% 0.460 [0.286, 0.605]
Total (95% CI) 96 8.0% 0.460 [0.286, 0.605]
Heterogeneity: not applicable
Test for overall effect: Z = 4.80 (P < 0.01)
SOLQ
Bulcun et al. (2015) 78 7.4% 0.535 [0.355, 0.677]
Total (95% CI) 78 7.4% 0.535 [0.355, 0.677]
Heterogeneity: not applicable
Test for overall effect: Z = 5.17 (P < 0.01)
SF−36
Vodanovich et al. (2015) 85 7.6% 0.459 [0.273, 0.612]
Total (95% CI) 85 7.6% 0.459 [0.273, 0.612]
Heterogeneity: not applicable
Test for overall effect: Z = 4.49 (P < 0.01)
Total (95% CI) 1289 100.0% 0.430 [0.352, 0.502]
Heterogeneity: Tau^2 = 0.02; Chi^2 = 31.81, df = 13 (P < 0.01); I^2 = 59%
Test for overall effect: Z = 9.78 (P < 0.01)
Test for subgroup differences: Chi^2 = 2.55, df = 6 (P = 0.86)
−0.5 0 0.5
b
Depression
Study or Subgroup
Total
Weight
Correlation IV, Random, 95% CI
Correlation IV, Random, 95% CI
QoL−B
Wilson et al. (a) (1997) 111 8.2% 0.550 [0.405, 0.668]
Chan et al. (2002) 93 7.8% 0.620 [0.476, 0.731]
Batchelor et al. (2011) 31 4.9% 0.120 [−0.245, 0.455]
Girón Moreno et al. (2013) 70 7.1% 0.120 [−0.118, 0.345]
Morsi et al. (2014) 33 5.1% 0.310 [−0.037, 0.590]
Gao et al. (2018) 163 8.9% 0.600 [0.492, 0.690]
Cordova−Rivera et al. (2019) 131 8.5% 0.383 [0.226, 0.520]
Total (95% CI) 632 50.4% 0.427 [0.270, 0.562]
Heterogeneity: Tau^2 = 0.04; Chi^2 = 27.7, df = 6 (P < 0.01); I^2 = 78%
Test for overall effect: Z = 4.99 (P < 0.01)
BHQ
Gao et al. (b) (2014) 144 8.7% 0.308 [0.152, 0.449]
Total (95% CI) 144 8.7% 0.308 [0.152, 0.449]
Heterogeneity: not applicable
Test for overall effect: Z = 3.78 (P < 0.01)
SGRQ
Oliveira et al. (2014) 207 9.2% 0.290 [0.160, 0.410]
Total (95% CI) 207 9.2% 0.290 [0.160, 0.410]
Heterogeneity: not applicable
Test for overall effect: Z = 4.26 (P < 0.01)
CAT
Galli et al. (c) (2019) 26 4.4% 0.493 [0.131, 0.739]
Total (95% CI) 26 4.4% 0.493 [0.131, 0.739]
Heterogeneity: not applicable
Test for overall effect: Z = 2.59 (P < 0.01)
CRDQ
De la Rosa Carrillo et al. (2020) 96 7.9% 0.570 [0.417, 0.691]
Total (95% CI) 96 7.9% 0.570 [0.417, 0.691]
Heterogeneity: not applicable
Test for overall effect: Z = 6.24 (P < 0.01)
SOLQ
Bulcun et al. (2015) 78 7.4% 0.535 [0.355, 0.677]
Total (95% CI) 78 7.4% 0.535 [0.355, 0.677]
Heterogeneity: not applicable
Test for overall effect: Z = 5.17 (P < 0.01)
SF−36
Lee et al. (2010) 27 4.5% 0.660 [0.374, 0.831]
Vodanovich et al. (2015) 85 7.6% 0.555 [0.388, 0.687]
Total (95% CI) 112 12.1% 0.581 [0.441, 0.693]
Heterogeneity: Tau^2 = 0; Chi^2 = 0.52, df = 1 (P = 0.47); I^2 = 0%
Test for overall effect: Z = 6.83 (P < 0.01)
Total (95% CI) 1295 100.0% 0.455 [0.362, 0.538]
Heterogeneity: Tau^2 = 0.03; Chi^2 = 46.68, df = 13 (P < 0.01); I^2 = 72%
Test for overall effect: Z = 8.68 (P < 0.01)
Test for subgroup differences: Chi^2 = 17.36, df = 6 (P < 0.01)
−0.5 0 0.5

Figure E3

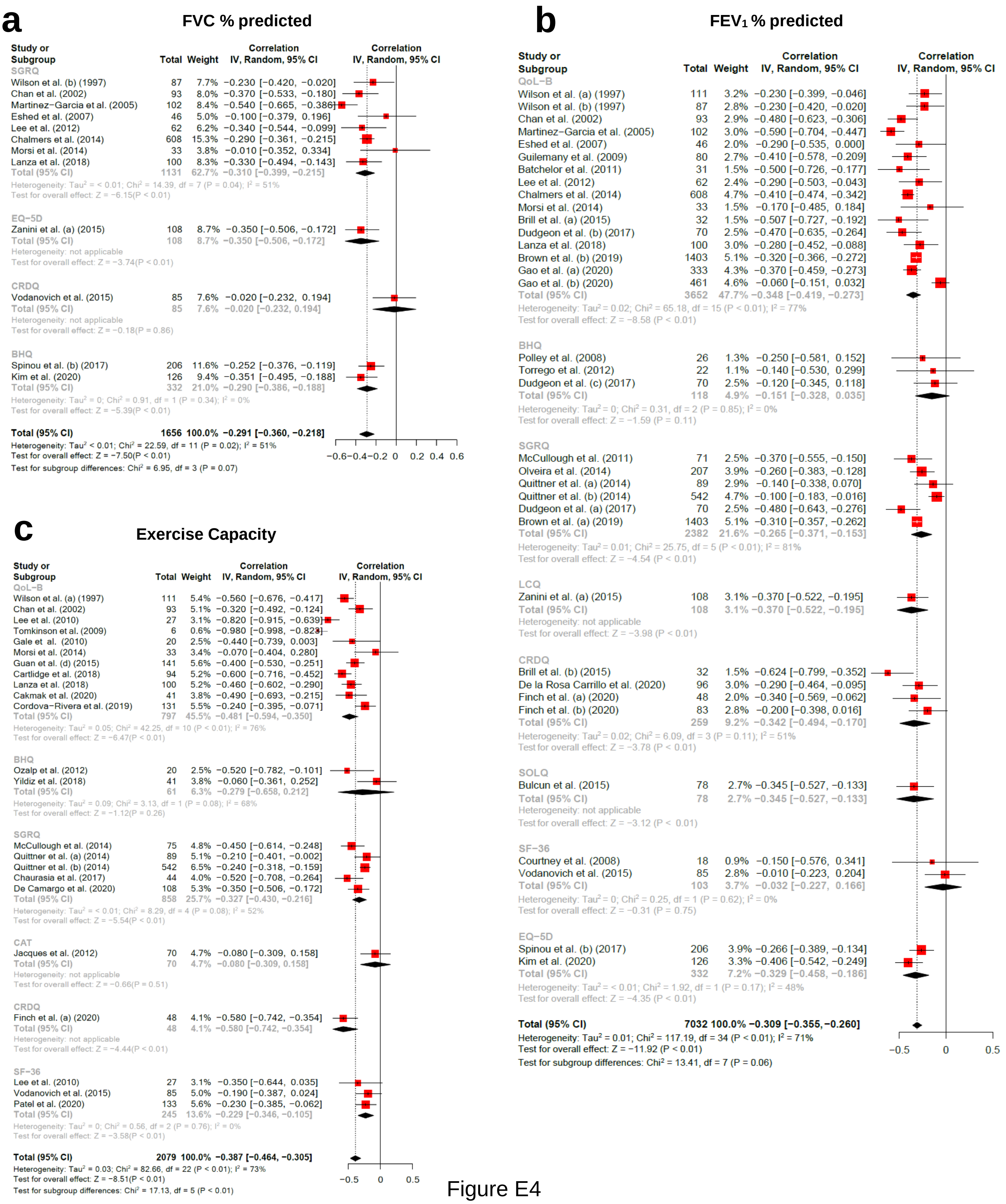
a
FVC % predicted
Study or Subgroup
Total
Weight
Correlation IV, Random, 95% CI
Correlation IV, Random, 95% CI
SGRQ
Wilson et al. (b) (1997) 87 7.7% −0.230 [−0.420, −0.020]
Chan et al. (2002) 93 8.0% −0.370 [−0.533, −0.180]
Martinez−Garcia et al. (2005) 102 8.4% −0.540 [−0.665, −0.386]
Eshed et al. (2007) 46 5.0% −0.100 [−0.379, 0.196]
Lee et al. (2012) 62 6.2% −0.340 [−0.544, −0.099]
Chalmers et al. (2014) 608 15.3% −0.290 [−0.361, −0.215]
Morsi et al. (2014) 33 3.8% −0.010 [−0.352, 0.334]
Lanza et al. (2018) 100 8.3% −0.330 [−0.494, −0.143]
Total (95% CI) 1131 62.7% −0.310 [−0.399, −0.215]
Heterogeneity: Tau² = < 0.01; Chi² = 14.39, df = 7 (P = 0.04); I² = 51%
Test for overall effect: Z = −6.15(P < 0.01)
EQ−5D
Zanini et al. (a) (2015) 108 8.7% −0.350 [−0.506, −0.172]
Total (95% CI) 108 8.7% −0.350 [−0.506, −0.172]
Heterogeneity: not applicable
Test for overall effect: Z = −3.74(P < 0.01)
CRDQ
Vodanovich et al. (2015) 85 7.6% −0.020 [−0.232, 0.194]
Total (95% CI) 85 7.6% −0.020 [−0.232, 0.194]
Heterogeneity: not applicable
Test for overall effect: Z = −0.18(P = 0.86)
BHQ
Spinou et al. (b) (2017) 206 11.6% −0.252 [−0.376, −0.119]
Kim et al. (2020) 126 9.4% −0.351 [−0.495, −0.188]
Total (95% CI) 332 21.0% −0.290 [−0.386, −0.188]
Heterogeneity: Tau² = 0; Chi² = 0.91, df = 1 (P = 0.34); I² = 0%
Test for overall effect: Z = −5.39(P < 0.01)
Total (95% CI) 1656 100.0% −0.291 [−0.360, −0.218]
Heterogeneity: Tau² < 0.01; Chi² = 22.59, df = 11 (P = 0.02); I² = 51%
Test for overall effect: Z = −7.50(P < 0.01)
Test for subgroup differences: Chi² = 6.95, df = 3 (P = 0.07)
−0.6 −0.4 −0.2 0 0.2 0.4 0.6
b
FEV₁ % predicted
Study or Subgroup
Total
Weight
Correlation IV, Random, 95% CI
Correlation IV, Random, 95% CI
QoL−B
Wilson et al. (a) (1997) 111 3.2% −0.230 [−0.399, −0.046]
Wilson et al. (b) (1997) 87 2.8% −0.230 [−0.420, −0.020]
Chan et al. (2002) 93 2.9% −0.480 [−0.623, −0.306]
Martinez−Garcia et al. (2005) 102 3.0% −0.590 [−0.704, −0.447]
Eshed et al. (2007) 46 2.0% −0.290 [−0.535, 0.000]
Guilemany et al. (2009) 80 2.7% −0.410 [−0.578, −0.209]
Batchelor et al. (2011) 31 1.5% −0.500 [−0.726, −0.177]
Lee et al. (2012) 62 2.4% −0.290 [−0.503, −0.043]
Chalmers et al. (2014) 608 4.7% −0.410 [−0.474, −0.342]
Morsi et al. (2014) 33 1.5% −0.170 [−0.485, 0.184]
Brill et al. (a) (2015) 32 1.5% −0.507 [−0.727, −0.192]
Dudgeon et al. (b) (2017) 70 2.5% −0.470 [−0.635, −0.264]
Lanza et al. (2018) 100 3.0% −0.280 [−0.452, −0.088]
Brown et al. (b) (2019) 1403 5.1% −0.320 [−0.366, −0.272]
Gao et al. (a) (2020) 333 4.3% −0.370 [−0.459, −0.273]
Gao et al. (b) (2020) 461 4.6% −0.060 [−0.151, 0.032]
Total (95% CI) 3652 47.7% −0.348 [−0.419, −0.273]
Heterogeneity: Tau² = 0.02; Chi² = 65.18, df = 15 (P < 0.01); I² = 77%
Test for overall effect: Z = −8.58 (P < 0.01)
BHQ
Polley et al. (2008) 26 1.3% −0.250 [−0.581, 0.152]
Torrego et al. (2012) 22 1.1% −0.140 [−0.530, 0.299]
Dudgeon et al. (c) (2017) 70 2.5% −0.120 [−0.345, 0.118]
Total (95% CI) 118 4.9% −0.151 [−0.328, 0.035]
Heterogeneity: Tau² = 0; Chi² = 0.31, df = 2 (P = 0.85); I² = 0%
Test for overall effect: Z = −1.59 (P = 0.11)
SGRQ
McCullough et al. (2011) 71 2.5% −0.370 [−0.555, −0.150]
Olveira et al. (2014) 207 3.9% −0.260 [−0.383, −0.128]
Quittner et al. (a) (2014) 89 2.9% −0.140 [−0.338, 0.070]
Quittner et al. (b) (2014) 542 4.7% −0.100 [−0.183, −0.016]
Dudgeon et al. (a) (2017) 70 2.5% −0.480 [−0.643, −0.276]
Brown et al. (a) (2019) 1403 5.1% −0.310 [−0.357, −0.262]
Total (95% CI) 2382 21.6% −0.265 [−0.371, −0.153]
Heterogeneity: Tau² = 0.01; Chi² = 25.75, df = 5 (P < 0.01); I² = 81%
Test for overall effect: Z = −4.54 (P < 0.01)
LCQ
Zanini et al. (a) (2015) 108 3.1% −0.370 [−0.522, −0.195]
Total (95% CI) 108 3.1% −0.370 [−0.522, −0.195]
Heterogeneity: not applicable
Test for overall effect: Z = −3.98 (P < 0.01)
CRDQ
Brill et al. (b) (2015) 32 1.5% −0.624 [−0.799, −0.352]
De la Rosa Carrillo et al. (2020) 96 3.0% −0.290 [−0.464, −0.095]
Finch et al. (a) (2020) 48 2.0% −0.340 [−0.569, −0.062]
Finch et al. (b) (2020) 83 2.8% −0.200 [−0.398, 0.016]
Total (95% CI) 259 9.2% −0.342 [−0.494, −0.170]
Heterogeneity: Tau² = 0.02; Chi² = 6.09, df = 3 (P = 0.11); I² = 51%
Test for overall effect: Z = −3.78 (P < 0.01)
SOLQ
Bulcun et al. (2015) 78 2.7% −0.345 [−0.527, −0.133]
Total (95% CI) 78 2.7% −0.345 [−0.527, −0.133]
Heterogeneity: not applicable
Test for overall effect: Z = −3.12 (P < 0.01)
SF−36
Courtney et al. (2008) 18 0.9% −0.150 [−0.576, 0.341]
Vodanovich et al. (2015) 85 2.8% −0.010 [−0.223, 0.204]
Total (95% CI) 103 3.7% −0.032 [−0.227, 0.166]
Heterogeneity: Tau² = 0; Chi² = 0.25, df = 1 (P = 0.62); I² = 0%
Test for overall effect: Z = −0.31 (P = 0.75)
EQ−5D
Spinou et al. (b) (2017) 206 3.9% −0.266 [−0.389, −0.134]
Kim et al. (2020) 126 3.3% −0.406 [−0.542, −0.249]
Total (95% CI) 332 7.2% −0.329 [−0.458, −0.186]
Heterogeneity: Tau² = < 0.01; Chi² = 1.92, df = 1 (P = 0.17); I² = 48%
Test for overall effect: Z = −4.35 (P < 0.01)
Total (95% CI) 7032 100.0% −0.309 [−0.355, −0.260]
Heterogeneity: Tau² = 0.01; Chi² = 117.19, df = 34 (P < 0.01); I² = 71%
Test for overall effect: Z = −11.92 (P < 0.01)
Test for subgroup differences: Chi² = 13.41, df = 7 (P = 0.06)
−0.5 0 0.5
c
Exercise Capacity
Study or Subgroup
Total
Weight
Correlation IV, Random, 95% CI
Correlation IV, Random, 95% CI
QoL−B
Wilson et al. (a) (1997) 111 5.4% −0.560 [−0.676, −0.417]
Chan et al. (2002) 93 5.1% −0.320 [−0.492, −0.124]
Lee et al. (2010) 27 3.1% −0.820 [−0.915, −0.639]
Tomkinson et al. (2009) 6 0.6% −0.980 [−0.998, −0.823]
Gale et al. (2010) 20 2.5% −0.440 [−0.739, 0.003]
Morsi et al. (2014) 33 3.4% −0.070 [−0.404, 0.280]
Guan et al. (d) (2015) 141 5.6% −0.400 [−0.530, −0.251]
Cartlidge et al. (2018) 94 5.2% −0.600 [−0.716, −0.452]
Lanza et al. (2018) 100 5.2% −0.460 [−0.602, −0.290]
Cakmak et al. (2020) 41 3.8% −0.490 [−0.693, −0.215]
Cordova−Rivera et al. (2019) 131 5.5% −0.240 [−0.395, −0.071]
Total (95% CI) 797 45.5% −0.481 [−0.594, −0.350]
Heterogeneity: Tau² = 0.05; Chi² = 42.25, df = 10 (P < 0.01); I² = 76%
Test for overall effect: Z = −6.47(P < 0.01)
BHQ
Ozalp et al. (2012) 20 2.5% −0.520 [−0.782, −0.101]
Yildiz et al. (2018) 41 3.8% −0.060 [−0.361, 0.252]
Total (95% CI) 61 6.3% −0.279 [−0.658, 0.212]
Heterogeneity: Tau² = 0.09; Chi² = 3.13, df = 1 (P = 0.08); I² = 68%
Test for overall effect: Z = −1.12(P = 0.26)
SGRQ
McCullough et al. (2014) 75 4.8% −0.450 [−0.614, −0.248]
Quittner et al. (a) (2014) 89 5.1% −0.210 [−0.401, −0.002]
Quittner et al. (b) (2014) 542 6.5% −0.240 [−0.318, −0.159]
Chaurasia et al. (2017) 44 4.0% −0.520 [−0.708, −0.264]
De Camargo et al. (2020) 108 5.3% −0.350 [−0.506, −0.172]
Total (95% CI) 858 25.7% −0.327 [−0.430, −0.216]
Heterogeneity: Tau² = < 0.01; Chi² = 8.29, df = 4 (P = 0.08); I² = 52%
Test for overall effect: Z = −5.54(P < 0.01)
CAT
Jacques et al. (2012) 70 4.7% −0.080 [−0.309, 0.158]
Total (95% CI) 70 4.7% −0.080 [−0.309, 0.158]
Heterogeneity: not applicable
Test for overall effect: Z = −0.66(P = 0.51)
CRDQ
Finch et al. (a) (2020) 48 4.1% −0.580 [−0.742, −0.354]
Total (95% CI) 48 4.1% −0.580 [−0.742, −0.354]
Heterogeneity: not applicable
Test for overall effect: Z = −4.44(P < 0.01)
SF−36
Lee et al. (2010) 27 3.1% −0.350 [−0.644, 0.035]
Vodanovich et al. (2015) 85 5.0% −0.190 [−0.387, 0.024]
Patel et al. (2020) 133 5.6% −0.230 [−0.385, −0.062]
Total (95% CI) 245 13.6% −0.229 [−0.346, −0.105]
Heterogeneity: Tau² = 0; Chi² = 0.56, df = 2 (P = 0.76); I² = 0%
Test for overall effect: Z = −3.58(P < 0.01)
Total (95% CI) 2079 100.0% −0.387 [−0.464, −0.305]
Heterogeneity: Tau² = 0.03; Chi² = 82.66, df = 22 (P < 0.01); I² = 73%
Test for overall effect: Z = −8.51(P < 0.01)
Test for subgroup differences: Chi² = 17.13, df = 5 (P < 0.01)
−0.5 0 0.5

Figure E4

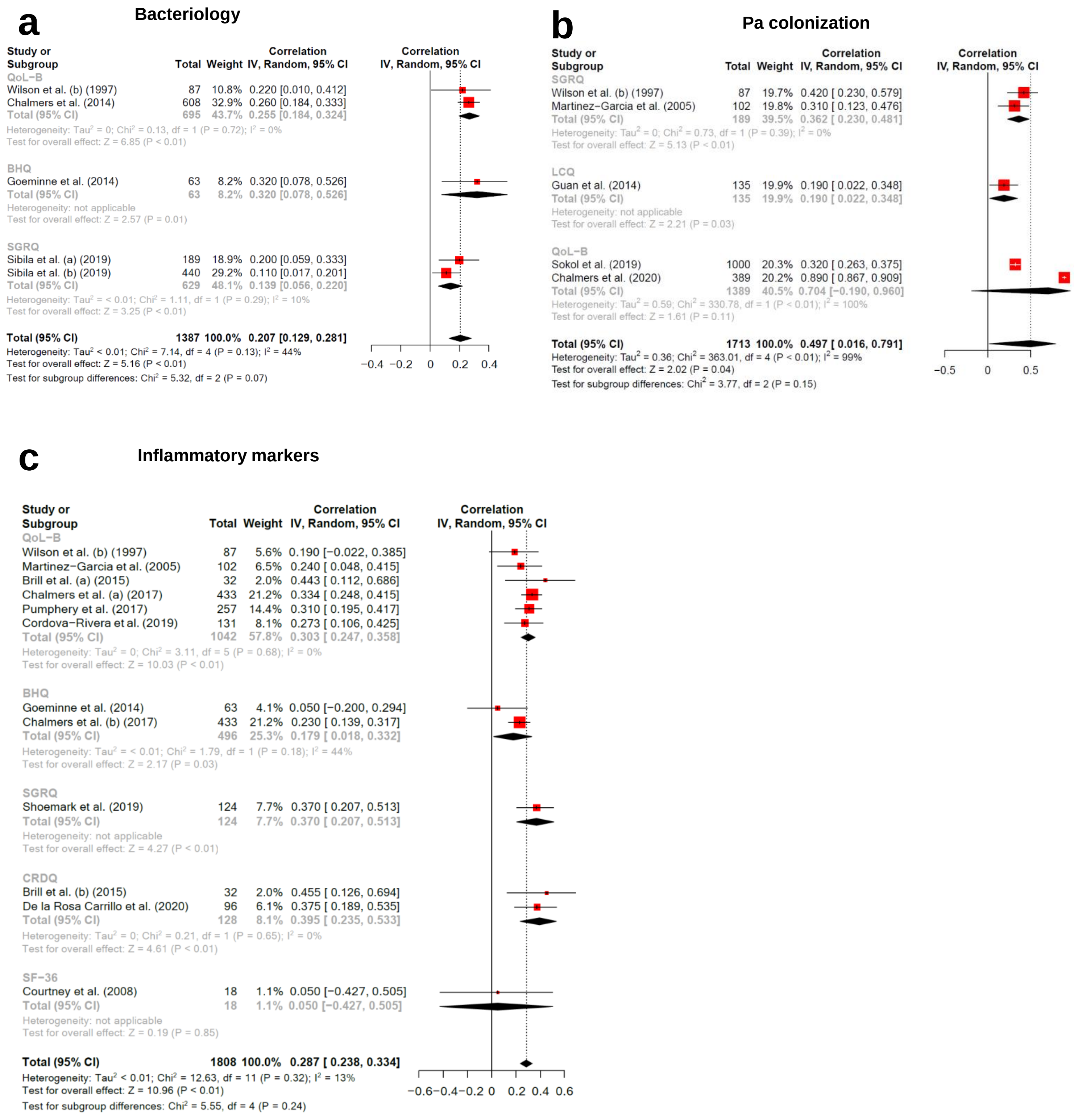
a
Bacteriology
Study or Subgroup
Total Weight Correlation IV, Random, 95% CI
Correlation IV, Random, 95% CI
QoL−B
Wilson et al. (b) (1997) 87 10.8% 0.220 [0.010, 0.412]
Chalmers et al. (2014) 608 32.9% 0.260 [0.184, 0.333]
Total (95% CI) 695 43.7% 0.255 [0.184, 0.324]
Heterogeneity: Tau² = 0; Chi² = 0.13, df = 1 (P = 0.72); I² = 0%
Test for overall effect: Z = 6.85 (P < 0.01)
BHQ
Goeminne et al. (2014) 63 8.2% 0.320 [0.078, 0.526]
Total (95% CI) 63 8.2% 0.320 [0.078, 0.526]
Heterogeneity: not applicable
Test for overall effect: Z = 2.57 (P = 0.01)
SGRQ
Sibila et al. (a) (2019) 189 18.9% 0.200 [0.059, 0.333]
Sibila et al. (b) (2019) 440 29.2% 0.110 [0.017, 0.201]
Total (95% CI) 629 48.1% 0.139 [0.056, 0.220]
Heterogeneity: Tau² = < 0.01; Chi² = 1.11, df = 1 (P = 0.29); I² = 10%
Test for overall effect: Z = 3.25 (P < 0.01)
Total (95% CI) 1387 100.0% 0.207 [0.129, 0.281]
Heterogeneity: Tau² < 0.01; Chi² = 7.14, df = 4 (P = 0.13); I² = 44%
Test for overall effect: Z = 5.16 (P < 0.01)
Test for subgroup differences: Chi² = 5.32, df = 2 (P = 0.07)
−0.4 −0.2 0 0.2 0.4
b
Pa colonization
Study or Subgroup
Total Weight Correlation IV, Random, 95% CI
Correlation IV, Random, 95% CI
SGRQ
Wilson et al. (b) (1997) 87 19.7% 0.420 [0.230, 0.579]
Martinez−Garcia et al. (2005) 102 19.8% 0.310 [0.123, 0.476]
Total (95% CI) 189 39.5% 0.362 [0.230, 0.481]
Heterogeneity: Tau² = 0; Chi² = 0.73, df = 1 (P = 0.39); I² = 0%
Test for overall effect: Z = 5.13 (P < 0.01)
LCQ
Guan et al. (2014) 135 19.9% 0.190 [0.022, 0.348]
Total (95% CI) 135 19.9% 0.190 [0.022, 0.348]
Heterogeneity: not applicable
Test for overall effect: Z = 2.21 (P = 0.03)
QoL−B
Sokol et al. (2019) 1000 20.3% 0.320 [0.263, 0.375]
Chalmers et al. (2020) 389 20.2% 0.890 [0.867, 0.909]
Total (95% CI) 1389 40.5% 0.704 [−0.190, 0.960]
Heterogeneity: Tau² = 0.59; Chi² = 330.78, df = 1 (P < 0.01); I² = 100%
Test for overall effect: Z = 1.61 (P = 0.11)
Total (95% CI) 1713 100.0% 0.497 [0.016, 0.791]
Heterogeneity: Tau² = 0.36; Chi² = 363.01, df = 4 (P < 0.01); I² = 99%
Test for overall effect: Z = 2.02 (P = 0.04)
Test for subgroup differences: Chi² = 3.77, df = 2 (P = 0.15)
−0.5 0 0.5
c
Inflammatory markers
Study or Subgroup
Total Weight Correlation IV, Random, 95% CI
Correlation IV, Random, 95% CI
QoL−B
Wilson et al. (b) (1997) 87 5.6% 0.190 [−0.022, 0.385]
Martinez−Garcia et al. (2005) 102 6.5% 0.240 [0.048, 0.415]
Brill et al. (a) (2015) 32 2.0% 0.443 [0.112, 0.686]
Chalmers et al. (a) (2017) 433 21.2% 0.334 [0.248, 0.415]
Pumphery et al. (2017) 257 14.4% 0.310 [0.195, 0.417]
Cordova−Rivera et al. (2019) 131 8.1% 0.273 [0.106, 0.425]
Total (95% CI) 1042 57.8% 0.303 [0.247, 0.358]
Heterogeneity: Tau² = 0; Chi² = 3.11, df = 5 (P = 0.68); I² = 0%
Test for overall effect: Z = 10.03 (P < 0.01)
BHQ
Goeminne et al. (2014) 63 4.1% 0.050 [−0.200, 0.294]
Chalmers et al. (b) (2017) 433 21.2% 0.230 [0.139, 0.317]
Total (95% CI) 496 25.3% 0.179 [0.018, 0.332]
Heterogeneity: Tau² = < 0.01; Chi² = 1.79, df = 1 (P = 0.18); I² = 44%
Test for overall effect: Z = 2.17 (P = 0.03)
SGRQ
Shoemark et al. (2019) 124 7.7% 0.370 [0.207, 0.513]
Total (95% CI) 124 7.7% 0.370 [0.207, 0.513]
Heterogeneity: not applicable
Test for overall effect: Z = 4.27 (P < 0.01)
CRDQ
Brill et al. (b) (2015) 32 2.0% 0.455 [0.126, 0.694]
De la Rosa Carrillo et al. (2020) 96 6.1% 0.375 [0.189, 0.535]
Total (95% CI) 128 8.1% 0.395 [0.235, 0.533]
Heterogeneity: Tau² = 0; Chi² = 0.21, df = 1 (P = 0.65); I² = 0%
Test for overall effect: Z = 4.61 (P < 0.01)
SF−36
Courtney et al. (2008) 18 1.1% 0.050 [−0.427, 0.505]
Total (95% CI) 18 1.1% 0.050 [−0.427, 0.505]
Heterogeneity: not applicable
Test for overall effect: Z = 0.19 (P = 0.85)
Total (95% CI) 1808 100.0% 0.287 [0.238, 0.334]
Heterogeneity: Tau² < 0.01; Chi² = 12.63, df = 11 (P = 0.32); I² = 13%
Test for overall effect: Z = 10.96 (P < 0.01)
Test for subgroup differences: Chi² = 5.55, df = 4 (P = 0.24)
−0.6 −0.4 −0.2 0 0.2 0.4 0.6

Figure E5

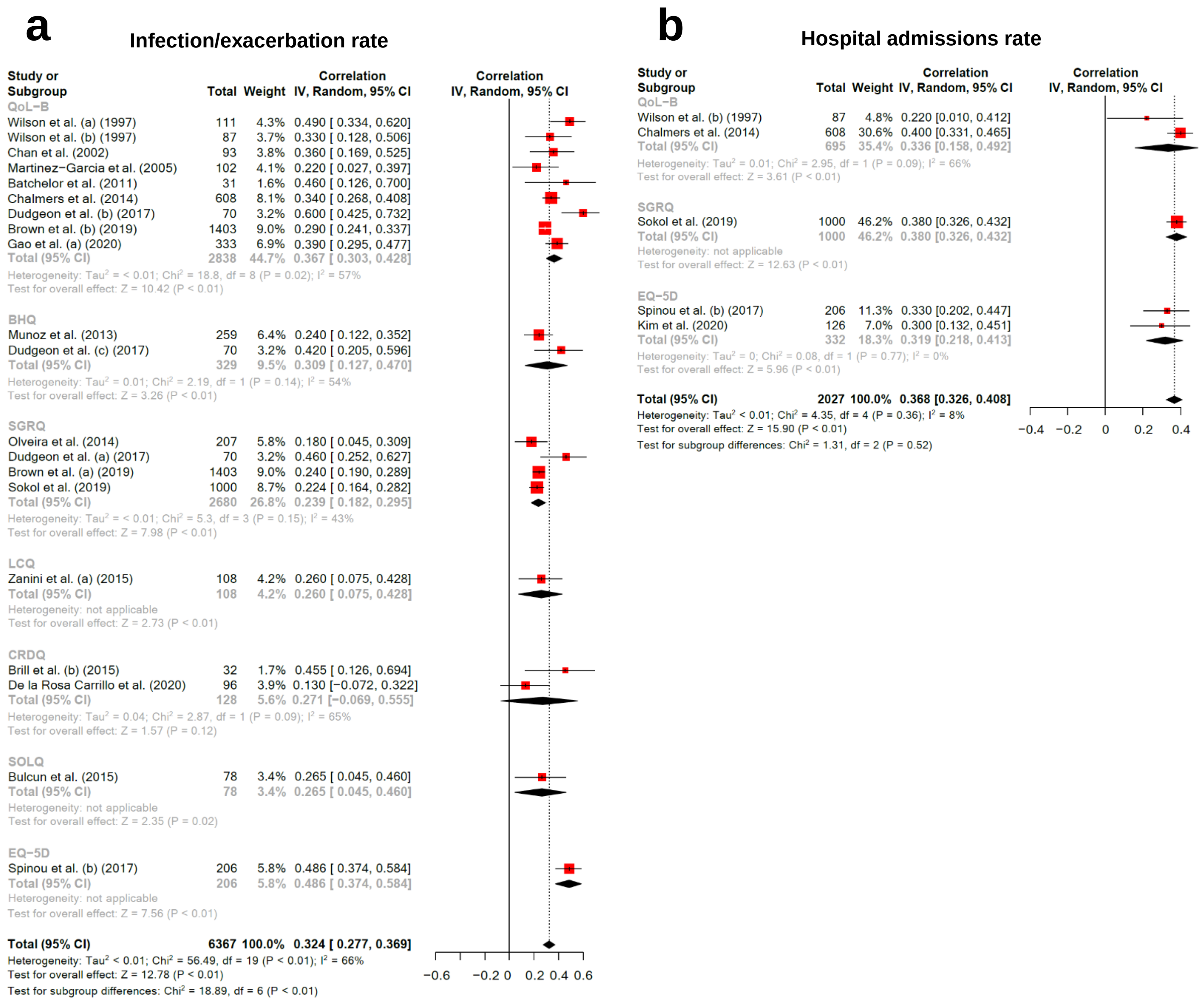
a
Infection/exacerbation rate
Study or Subgroup | Total | Weight | Correlation IV, Random, 95% CI
Correlation IV, Random, 95% CI
QoL−B
Wilson et al. (a) (1997) 111 4.3% 0.490 [0.334, 0.620]
Wilson et al. (b) (1997) 87 3.7% 0.330 [0.128, 0.506]
Chan et al. (2002) 93 3.8% 0.360 [0.169, 0.525]
Martinez−Garcia et al. (2005) 102 4.1% 0.220 [0.027, 0.397]
Batchelor et al. (2011) 31 1.6% 0.460 [0.126, 0.700]
Chalmers et al. (2014) 608 8.1% 0.340 [0.268, 0.408]
Dudgeon et al. (b) (2017) 70 3.2% 0.600 [0.425, 0.732]
Brown et al. (b) (2019) 1403 9.0% 0.290 [0.241, 0.337]
Gao et al. (a) (2020) 333 6.9% 0.390 [0.295, 0.477]
Total (95% CI) 2838 44.7% 0.367 [0.303, 0.428]
Heterogeneity: Tau² = < 0.01; Chi² = 18.8, df = 8 (P = 0.02); I² = 57%
Test for overall effect: Z = 10.42 (P < 0.01)
BHQ
Munoz et al. (2013) 259 6.4% 0.240 [0.122, 0.352]
Dudgeon et al. (c) (2017) 70 3.2% 0.420 [0.205, 0.596]
Total (95% CI) 329 9.5% 0.309 [0.127, 0.470]
Heterogeneity: Tau² = 0.01; Chi² = 2.19, df = 1 (P = 0.14); I² = 54%
Test for overall effect: Z = 3.26 (P < 0.01)
SGRQ
Olveira et al. (2014) 207 5.8% 0.180 [0.045, 0.309]
Dudgeon et al. (a) (2017) 70 3.2% 0.460 [0.252, 0.627]
Brown et al. (a) (2019) 1403 9.0% 0.240 [0.190, 0.289]
Sokol et al. (2019) 1000 8.7% 0.224 [0.164, 0.282]
Total (95% CI) 2680 26.8% 0.239 [0.182, 0.295]
Heterogeneity: Tau² = < 0.01; Chi² = 5.3, df = 3 (P = 0.15); I² = 43%
Test for overall effect: Z = 7.98 (P < 0.01)
LCQ
Zanini et al. (a) (2015) 108 4.2% 0.260 [0.075, 0.428]
Total (95% CI) 108 4.2% 0.260 [0.075, 0.428]
Heterogeneity: not applicable
Test for overall effect: Z = 2.73 (P < 0.01)
CRDQ
Brill et al. (b) (2015) 32 1.7% 0.455 [0.126, 0.694]
De la Rosa Carrillo et al. (2020) 96 3.9% 0.130 [−0.072, 0.322]
Total (95% CI) 128 5.6% 0.271 [−0.069, 0.555]
Heterogeneity: Tau² = 0.04; Chi² = 2.87, df = 1 (P = 0.09); I² = 65%
Test for overall effect: Z = 1.57 (P = 0.12)
SOLQ
Bulcun et al. (2015) 78 3.4% 0.265 [0.045, 0.460]
Total (95% CI) 78 3.4% 0.265 [0.045, 0.460]
Heterogeneity: not applicable
Test for overall effect: Z = 2.35 (P = 0.02)
EQ−5D
Spinou et al. (b) (2017) 206 5.8% 0.486 [0.374, 0.584]
Total (95% CI) 206 5.8% 0.486 [0.374, 0.584]
Heterogeneity: not applicable
Test for overall effect: Z = 7.56 (P < 0.01)
Total (95% CI) 6367 100.0% 0.324 [0.277, 0.369]
Heterogeneity: Tau² < 0.01; Chi² = 56.49, df = 19 (P < 0.01); I² = 66%
Test for overall effect: Z = 12.78 (P < 0.01)
Test for subgroup differences: Chi² = 18.89, df = 6 (P < 0.01)
−0.6 −0.2 0 0.2 0.4 0.6
b
Hospital admissions rate
Study or Subgroup | Total | Weight | Correlation IV, Random, 95% CI
Correlation IV, Random, 95% CI
QoL−B
Wilson et al. (b) (1997) 87 4.8% 0.220 [0.010, 0.412]
Chalmers et al. (2014) 608 30.6% 0.400 [0.331, 0.465]
Total (95% CI) 695 35.4% 0.336 [0.158, 0.492]
Heterogeneity: Tau² = 0.01; Chi² = 2.95, df = 1 (P = 0.09); I² = 66%
Test for overall effect: Z = 3.61 (P < 0.01)
SGRQ
Sokol et al. (2019) 1000 46.2% 0.380 [0.326, 0.432]
Total (95% CI) 1000 46.2% 0.380 [0.326, 0.432]
Heterogeneity: not applicable
Test for overall effect: Z = 12.63 (P < 0.01)
EQ−5D
Spinou et al. (b) (2017) 206 11.3% 0.330 [0.202, 0.447]
Kim et al. (2020) 126 7.0% 0.300 [0.132, 0.451]
Total (95% CI) 332 18.3% 0.319 [0.218, 0.413]
Heterogeneity: Tau² = 0; Chi² = 0.08, df = 1 (P = 0.77); I² = 0%
Test for overall effect: Z = 5.96 (P < 0.01)
Total (95% CI) 2027 100.0% 0.368 [0.326, 0.408]
Heterogeneity: Tau² < 0.01; Chi² = 4.35, df = 4 (P = 0.36); I² = 8%
Test for overall effect: Z = 15.90 (P < 0.01)
Test for subgroup differences: Chi² = 1.31, df = 2 (P = 0.52)
−0.4 −0.2 0 0.2 0.4

Figure E6

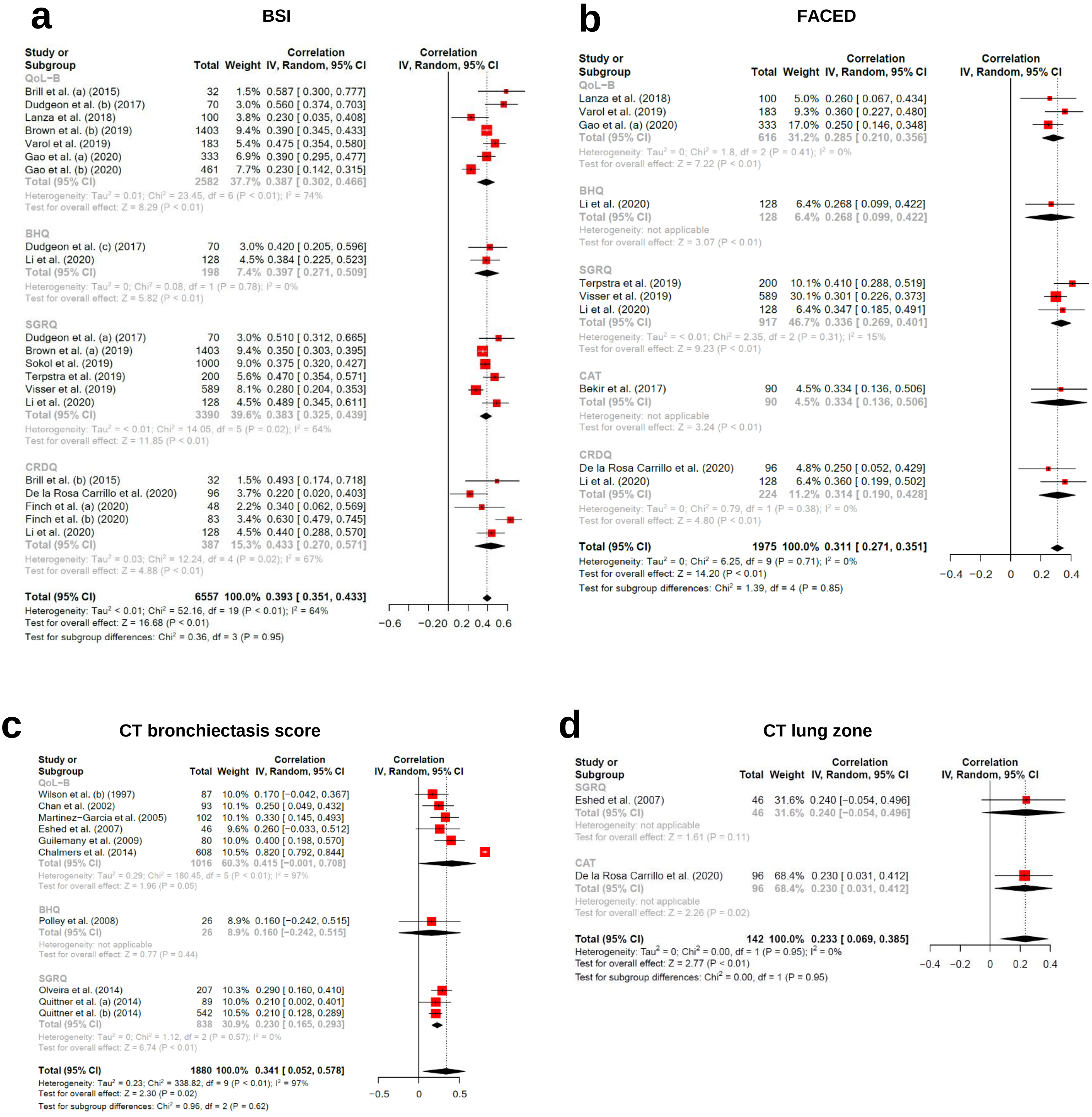

a
BSI
Study or Subgroup | Total | Weight | Correlation IV, Random, 95% CI
QoL−B
Brill et al. (a) (2015) 32 1.5% 0.587 [0.300, 0.777]
Dudgeon et al. (b) (2017) 70 3.0% 0.560 [0.374, 0.703]
Lanza et al. (2018) 100 3.8% 0.230 [0.035, 0.408]
Brown et al. (b) (2019) 1403 9.4% 0.390 [0.345, 0.433]
Varol et al. (2019) 183 5.4% 0.475 [0.354, 0.580]
Gao et al. (a) (2020) 333 6.9% 0.390 [0.295, 0.477]
Gao et al. (b) (2020) 461 7.7% 0.230 [0.142, 0.315]
Total (95% CI) 2582 37.7% 0.387 [0.302, 0.466]
Heterogeneity: Tau² = 0.01; Chi² = 23.45, df = 6 (P < 0.01); I² = 74%
Test for overall effect: Z = 8.29 (P < 0.01)
BHQ
Dudgeon et al. (c) (2017) 70 3.0% 0.420 [0.205, 0.596]
Li et al. (2020) 128 4.5% 0.384 [0.225, 0.523]
Total (95% CI) 198 7.4% 0.397 [0.271, 0.509]
Heterogeneity: Tau² = 0; Chi² = 0.08, df = 1 (P = 0.78); I² = 0%
Test for overall effect: Z = 5.82 (P < 0.01)
SGRQ
Dudgeon et al. (a) (2017) 70 3.0% 0.510 [0.312, 0.665]
Brown et al. (a) (2019) 1403 9.4% 0.350 [0.303, 0.395]
Sokol et al. (2019) 1000 9.0% 0.375 [0.320, 0.427]
Terpstra et al. (2019) 200 5.6% 0.470 [0.354, 0.571]
Visser et al. (2019) 589 8.1% 0.280 [0.204, 0.353]
Li et al. (2020) 128 4.5% 0.489 [0.345, 0.611]
Total (95% CI) 3390 39.6% 0.383 [0.325, 0.439]
Heterogeneity: Tau² = < 0.01; Chi² = 14.05, df = 5 (P = 0.02); I² = 64%
Test for overall effect: Z = 11.85 (P < 0.01)
CRDQ
Brill et al. (b) (2015) 32 1.5% 0.493 [0.174, 0.718]
De la Rosa Carrillo et al. (2020) 96 3.7% 0.220 [0.020, 0.403]
Finch et al. (a) (2020) 48 2.2% 0.340 [0.062, 0.569]
Finch et al. (b) (2020) 83 3.4% 0.630 [0.479, 0.745]
Li et al. (2020) 128 4.5% 0.440 [0.288, 0.570]
Total (95% CI) 387 15.3% 0.433 [0.270, 0.571]
Heterogeneity: Tau² = 0.03; Chi² = 12.24, df = 4 (P = 0.02); I² = 67%
Test for overall effect: Z = 4.88 (P < 0.01)
Total (95% CI) 6557 100.0% 0.393 [0.351, 0.433]
Heterogeneity: Tau² < 0.01; Chi² = 52.16, df = 19 (P < 0.01); I² = 64%
Test for overall effect: Z = 16.68 (P < 0.01)
Test for subgroup differences: Chi² = 0.36, df = 3 (P = 0.95)
−0.6 −0.20 0.2 0.4 0.6
b
FACED
Study or Subgroup | Total | Weight | Correlation IV, Random, 95% CI
QoL−B
Lanza et al. (2018) 100 5.0% 0.260 [0.067, 0.434]
Varol et al. (2019) 183 9.3% 0.360 [0.227, 0.480]
Gao et al. (a) (2020) 333 17.0% 0.250 [0.146, 0.348]
Total (95% CI) 616 31.2% 0.285 [0.210, 0.356]
Heterogeneity: Tau² = 0; Chi² = 1.8, df = 2 (P = 0.41); I² = 0%
Test for overall effect: Z = 7.22 (P < 0.01)
BHQ
Li et al. (2020) 128 6.4% 0.268 [0.099, 0.422]
Total (95% CI) 128 6.4% 0.268 [0.099, 0.422]
Heterogeneity: not applicable
Test for overall effect: Z = 3.07 (P < 0.01)
SGRQ
Terpstra et al. (2019) 200 10.1% 0.410 [0.288, 0.519]
Visser et al. (2019) 589 30.1% 0.301 [0.226, 0.373]
Li et al. (2020) 128 6.4% 0.347 [0.185, 0.491]
Total (95% CI) 917 46.7% 0.336 [0.269, 0.401]
Heterogeneity: Tau² = < 0.01; Chi² = 2.35, df = 2 (P = 0.31); I² = 15%
Test for overall effect: Z = 9.23 (P < 0.01)
CAT
Bekir et al. (2017) 90 4.5% 0.334 [0.136, 0.506]
Total (95% CI) 90 4.5% 0.334 [0.136, 0.506]
Heterogeneity: not applicable
Test for overall effect: Z = 3.24 (P < 0.01)
CRDQ
De la Rosa Carrillo et al. (2020) 96 4.8% 0.250 [0.052, 0.429]
Li et al. (2020) 128 6.4% 0.360 [0.199, 0.502]
Total (95% CI) 224 11.2% 0.314 [0.190, 0.428]
Heterogeneity: Tau² = 0; Chi² = 0.79, df = 1 (P = 0.38); I² = 0%
Test for overall effect: Z = 4.80 (P < 0.01)
Total (95% CI) 1975 100.0% 0.311 [0.271, 0.351]
Heterogeneity: Tau² = 0; Chi² = 6.25, df = 9 (P = 0.71); I² = 0%
Test for overall effect: Z = 14.20 (P < 0.01)
Test for subgroup differences: Chi² = 1.39, df = 4 (P = 0.85)
−0.4 −0.2 0 0.2 0.4
c
CT bronchiectasis score
Study or Subgroup | Total | Weight | Correlation IV, Random, 95% CI
QoL−B
Wilson et al. (b) (1997) 87 10.0% 0.170 [−0.042, 0.367]
Chan et al. (2002) 93 10.1% 0.250 [0.049, 0.432]
Martinez-Garcia et al. (2005) 102 10.1% 0.330 [0.145, 0.493]
Eshed et al. (2007) 46 9.6% 0.260 [−0.033, 0.512]
Guilemany et al. (2009) 80 10.0% 0.400 [0.198, 0.570]
Chalmers et al. (2014) 608 10.5% 0.820 [0.792, 0.844]
Total (95% CI) 1016 60.3% 0.415 [−0.001, 0.708]
Heterogeneity: Tau² = 0.29; Chi² = 180.45, df = 5 (P < 0.01); I² = 97%
Test for overall effect: Z = 1.96 (P = 0.05)
BHQ
Polley et al. (2008) 26 8.9% 0.160 [−0.242, 0.515]
Total (95% CI) 26 8.9% 0.160 [−0.242, 0.515]
Heterogeneity: not applicable
Test for overall effect: Z = 0.77 (P = 0.44)
SGRQ
Olveira et al. (2014) 207 10.3% 0.290 [0.160, 0.410]
Quittner et al. (a) (2014) 89 10.0% 0.210 [0.002, 0.401]
Quittner et al. (b) (2014) 542 10.5% 0.210 [0.128, 0.289]
Total (95% CI) 838 30.9% 0.230 [0.165, 0.293]
Heterogeneity: Tau² = 0; Chi² = 1.12, df = 2 (P = 0.57); I² = 0%
Test for overall effect: Z = 6.74 (P < 0.01)
Total (95% CI) 1880 100.0% 0.341 [0.052, 0.578]
Heterogeneity: Tau² = 0.23; Chi² = 338.82, df = 9 (P < 0.01); I² = 97%
Test for overall effect: Z = 2.30 (P = 0.02)
Test for subgroup differences: Chi² = 0.96, df = 2 (P = 0.62)
−0.5 0 0.5
d
CT lung zone
Study or Subgroup | Total | Weight | Correlation IV, Random, 95% CI
SGRQ
Eshed et al. (2007) 46 31.6% 0.240 [−0.054, 0.496]
Total (95% CI) 46 31.6% 0.240 [−0.054, 0.496]
Heterogeneity: not applicable
Test for overall effect: Z = 1.61 (P = 0.11)
CAT
De la Rosa Carrillo et al. (2020) 96 68.4% 0.230 [0.031, 0.412]
Total (95% CI) 96 68.4% 0.230 [0.031, 0.412]
Heterogeneity: not applicable
Test for overall effect: Z = 2.26 (P = 0.02)
Total (95% CI) 142 100.0% 0.233 [0.069, 0.385]
Heterogeneity: Tau² = 0; Chi² = 0.00, df = 1 (P = 0.95); I² = 0%
Test for overall effect: Z = 2.77 (P < 0.01)
Test for subgroup differences: Chi² = 0.00, df = 1 (P = 0.95)
−0.4 −0.2 0 0.2 0.4

Figure E7

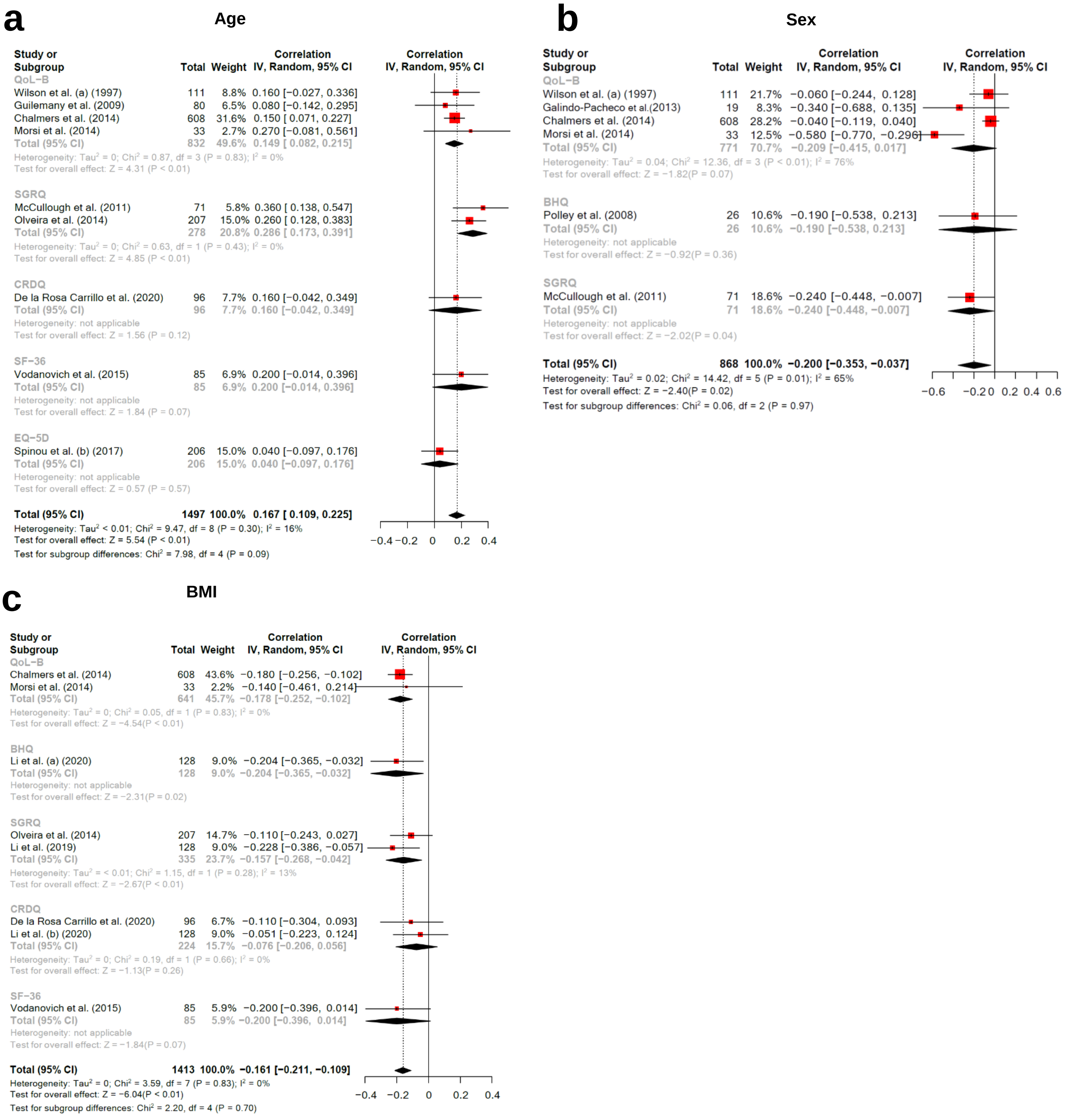

a
Age
Study or Subgroup
Total
Weight
Correlation IV, Random, 95% CI
Correlation IV, Random, 95% CI
QoL−B
Wilson et al. (a) (1997) 111 8.8% 0.160 [−0.027, 0.336]
Guilemany et al. (2009) 80 6.5% 0.080 [−0.142, 0.295]
Chalmers et al. (2014) 608 31.6% 0.150 [0.071, 0.227]
Morsi et al. (2014) 33 2.7% 0.270 [−0.081, 0.561]
Total (95% CI) 832 49.6% 0.149 [0.082, 0.215]
Heterogeneity: Tau² = 0; Chi² = 0.87, df = 3 (P = 0.83); I² = 0%
Test for overall effect: Z = 4.31 (P < 0.01)
SGRQ
McCullough et al. (2011) 71 5.8% 0.360 [0.138, 0.547]
Olveira et al. (2014) 207 15.0% 0.260 [0.128, 0.383]
Total (95% CI) 278 20.8% 0.286 [0.173, 0.391]
Heterogeneity: Tau² = 0; Chi² = 0.63, df = 1 (P = 0.43); I² = 0%
Test for overall effect: Z = 4.85 (P < 0.01)
CRDQ
De la Rosa Carrillo et al. (2020) 96 7.7% 0.160 [−0.042, 0.349]
Total (95% CI) 96 7.7% 0.160 [−0.042, 0.349]
Heterogeneity: not applicable
Test for overall effect: Z = 1.56 (P = 0.12)
SF−36
Vodanovich et al. (2015) 85 6.9% 0.200 [−0.014, 0.396]
Total (95% CI) 85 6.9% 0.200 [−0.014, 0.396]
Heterogeneity: not applicable
Test for overall effect: Z = 1.84 (P = 0.07)
EQ−5D
Spinou et al. (b) (2017) 206 15.0% 0.040 [−0.097, 0.176]
Total (95% CI) 206 15.0% 0.040 [−0.097, 0.176]
Heterogeneity: not applicable
Test for overall effect: Z = 0.57 (P = 0.57)
Total (95% CI) 1497 100.0% 0.167 [0.109, 0.225]
Heterogeneity: Tau² < 0.01; Chi² = 9.47, df = 8 (P = 0.30); I² = 16%
Test for overall effect: Z = 5.54 (P < 0.01)
Test for subgroup differences: Chi² = 7.98, df = 4 (P = 0.09)
−0.4 −0.2 0 0.2 0.4
b
Sex
Study or Subgroup
Total
Weight
Correlation IV, Random, 95% CI
Correlation IV, Random, 95% CI
QoL−B
Wilson et al. (a) (1997) 111 21.7% −0.060 [−0.244, 0.128]
Galindo-Pacheco et al.(2013) 19 8.3% −0.340 [−0.688, 0.135]
Chalmers et al. (2014) 608 28.2% −0.040 [−0.119, 0.040]
Morsi et al. (2014) 33 12.5% −0.580 [−0.770, −0.296]
Total (95% CI) 771 70.7% −0.209 [−0.415, 0.017]
Heterogeneity: Tau² = 0.04; Chi² = 12.36, df = 3 (P < 0.01); I² = 76%
Test for overall effect: Z = −1.82(P = 0.07)
BHQ
Polley et al. (2008) 26 10.6% −0.190 [−0.538, 0.213]
Total (95% CI) 26 10.6% −0.190 [−0.538, 0.213]
Heterogeneity: not applicable
Test for overall effect: Z = −0.92(P = 0.36)
SGRQ
McCullough et al. (2011) 71 18.6% −0.240 [−0.448, −0.007]
Total (95% CI) 71 18.6% −0.240 [−0.448, −0.007]
Heterogeneity: not applicable
Test for overall effect: Z = −2.02(P = 0.04)
Total (95% CI) 868 100.0% −0.200 [−0.353, −0.037]
Heterogeneity: Tau² = 0.02; Chi² = 14.42, df = 5 (P = 0.01); I² = 65%
Test for overall effect: Z = −2.40(P = 0.02)
Test for subgroup differences: Chi² = 0.06, df = 2 (P = 0.97)
−0.6 −0.2 0 0.2 0.4 0.6
c
BMI
Study or Subgroup
Total
Weight
Correlation IV, Random, 95% CI
Correlation IV, Random, 95% CI
QoL−B
Chalmers et al. (2014) 608 43.6% −0.180 [−0.256, −0.102]
Morsi et al. (2014) 33 2.2% −0.140 [−0.461, 0.214]
Total (95% CI) 641 45.7% −0.178 [−0.252, −0.102]
Heterogeneity: Tau² = 0; Chi² = 0.05, df = 1 (P = 0.83); I² = 0%
Test for overall effect: Z = −4.54(P < 0.01)
BHQ
Li et al. (a) (2020) 128 9.0% −0.204 [−0.365, −0.032]
Total (95% CI) 128 9.0% −0.204 [−0.365, −0.032]
Heterogeneity: not applicable
Test for overall effect: Z = −2.31(P = 0.02)
SGRQ
Olveira et al. (2014) 207 14.7% −0.110 [−0.243, 0.027]
Li et al. (2019) 128 9.0% −0.228 [−0.386, −0.057]
Total (95% CI) 335 23.7% −0.157 [−0.268, −0.042]
Heterogeneity: Tau² = < 0.01; Chi² = 1.15, df = 1 (P = 0.28); I² = 13%
Test for overall effect: Z = −2.67(P < 0.01)
CRDQ
De la Rosa Carrillo et al. (2020) 96 6.7% −0.110 [−0.304, 0.093]
Li et al. (b) (2020) 128 9.0% −0.051 [−0.223, 0.124]
Total (95% CI) 224 15.7% −0.076 [−0.206, 0.056]
Heterogeneity: Tau² = 0; Chi² = 0.19, df = 1 (P = 0.66); I² = 0%
Test for overall effect: Z = −1.13(P = 0.26)
SF−36
Vodanovich et al. (2015) 85 5.9% −0.200 [−0.396, 0.014]
Total (95% CI) 85 5.9% −0.200 [−0.396, 0.014]
Heterogeneity: not applicable
Test for overall effect: Z = −1.84(P = 0.07)
Total (95% CI) 1413 100.0% −0.161 [−0.211, −0.109]
Heterogeneity: Tau² = 0; Chi² = 3.59, df = 7 (P = 0.83); I² = 0%
Test for overall effect: Z = −6.04(P < 0.01)
Test for subgroup differences: Chi² = 2.20, df = 4 (P = 0.70)
−0.4 −0.2 0 0.2 0.4

Figure E8

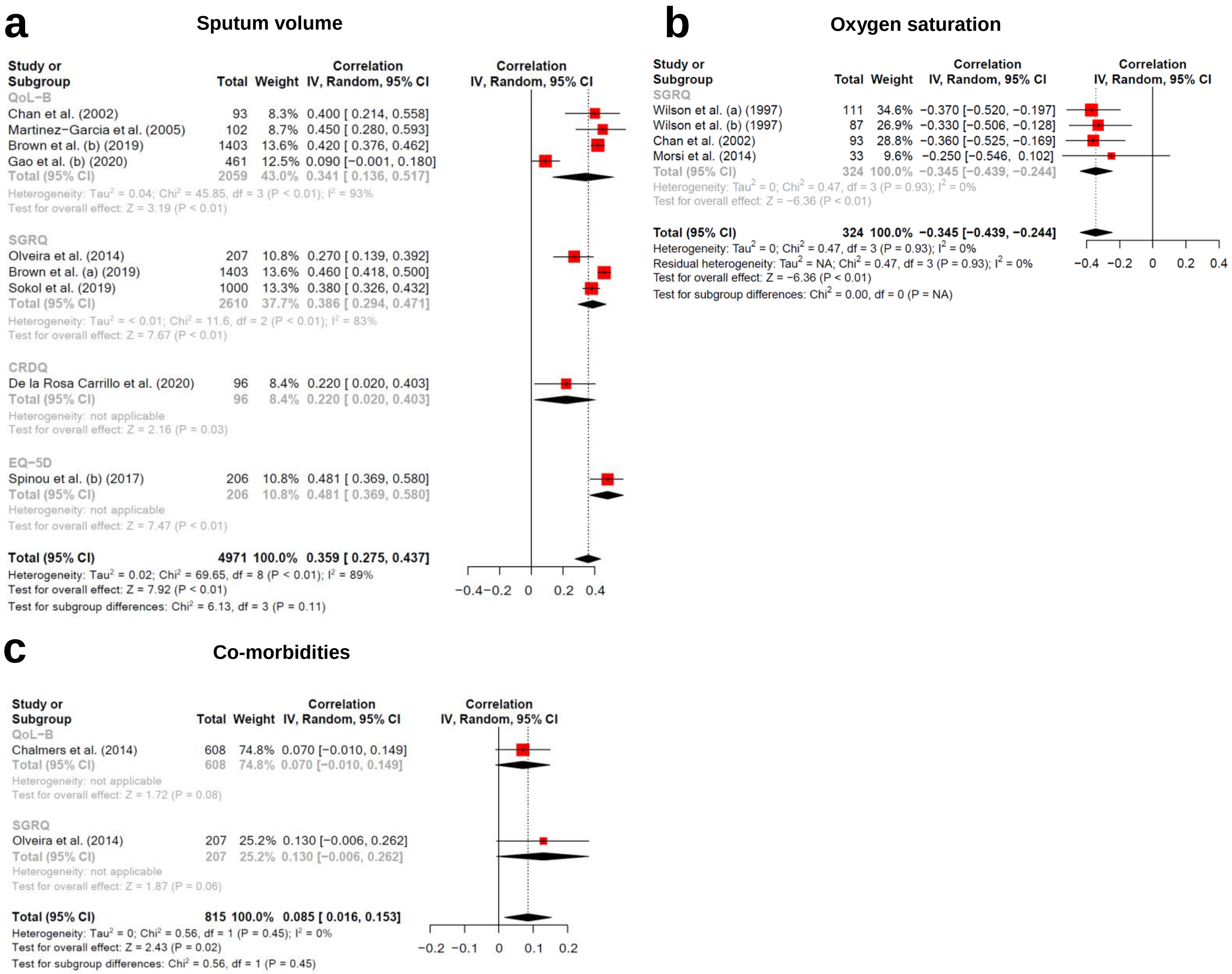
a
Sputum volume
Study or Subgroup
Total
Weight
Correlation IV, Random, 95% CI
QoL−B
Chan et al. (2002) 93 8.3% 0.400 [0.214, 0.558]
Martinez−Garcia et al. (2005) 102 8.7% 0.450 [0.280, 0.593]
Brown et al. (b) (2019) 1403 13.6% 0.420 [0.376, 0.462]
Gao et al. (b) (2020) 461 12.5% 0.090 [−0.001, 0.180]
Total (95% CI) 2059 43.0% 0.341 [0.136, 0.517]
Heterogeneity: Tau^2 = 0.04; Chi^2 = 45.85, df = 3 (P < 0.01); I^2 = 93%
Test for overall effect: Z = 3.19 (P < 0.01)
SGRQ
Olveira et al. (2014) 207 10.8% 0.270 [0.139, 0.392]
Brown et al. (a) (2019) 1403 13.6% 0.460 [0.418, 0.500]
Sokol et al. (2019) 1000 13.3% 0.380 [0.326, 0.432]
Total (95% CI) 2610 37.7% 0.386 [0.294, 0.471]
Heterogeneity: Tau^2 = < 0.01; Chi^2 = 11.6, df = 2 (P < 0.01); I^2 = 83%
Test for overall effect: Z = 7.67 (P < 0.01)
CRDQ
De la Rosa Carrillo et al. (2020) 96 8.4% 0.220 [0.020, 0.403]
Total (95% CI) 96 8.4% 0.220 [0.020, 0.403]
Heterogeneity: not applicable
Test for overall effect: Z = 2.16 (P = 0.03)
EQ−5D
Spinou et al. (b) (2017) 206 10.8% 0.481 [0.369, 0.580]
Total (95% CI) 206 10.8% 0.481 [0.369, 0.580]
Heterogeneity: not applicable
Test for overall effect: Z = 7.47 (P < 0.01)
Total (95% CI) 4971 100.0% 0.359 [0.275, 0.437]
Heterogeneity: Tau^2 = 0.02; Chi^2 = 69.65, df = 8 (P < 0.01); I^2 = 89%
Test for overall effect: Z = 7.92 (P < 0.01)
Test for subgroup differences: Chi^2 = 6.13, df = 3 (P = 0.11)
Correlation IV, Random, 95% CI
−0.4 −0.2 0 0.2 0.4
b
Oxygen saturation
Study or Subgroup
Total
Weight
Correlation IV, Random, 95% CI
SGRQ
Wilson et al. (a) (1997) 111 34.6% −0.370 [−0.520, −0.197]
Wilson et al. (b) (1997) 87 26.9% −0.330 [−0.506, −0.128]
Chan et al. (2002) 93 28.8% −0.360 [−0.525, −0.169]
Morsi et al. (2014) 33 9.6% −0.250 [−0.546, 0.102]
Total (95% CI) 324 100.0% −0.345 [−0.439, −0.244]
Heterogeneity: Tau^2 = 0; Chi^2 = 0.47, df = 3 (P = 0.93); I^2 = 0%
Test for overall effect: Z = −6.36 (P < 0.01)
Total (95% CI) 324 100.0% −0.345 [−0.439, −0.244]
Heterogeneity: Tau^2 = 0; Chi^2 = 0.47, df = 3 (P = 0.93); I^2 = 0%
Residual heterogeneity: Tau^2 = NA; Chi^2 = 0.47, df = 3 (P = 0.93); I^2 = 0%
Test for overall effect: Z = −6.36 (P < 0.01)
Test for subgroup differences: Chi^2 = 0.00, df = 0 (P = NA)
Correlation IV, Random, 95% CI
−0.4 −0.2 0 0.2 0.4
c
Co-morbidities
Study or Subgroup
Total
Weight
Correlation IV, Random, 95% CI
QoL−B
Chalmers et al. (2014) 608 74.8% 0.070 [−0.010, 0.149]
Total (95% CI) 608 74.8% 0.070 [−0.010, 0.149]
Heterogeneity: not applicable
Test for overall effect: Z = 1.72 (P = 0.08)
SGRQ
Olveira et al. (2014) 207 25.2% 0.130 [−0.006, 0.262]
Total (95% CI) 207 25.2% 0.130 [−0.006, 0.262]
Heterogeneity: not applicable
Test for overall effect: Z = 1.87 (P = 0.06)
Total (95% CI) 815 100.0% 0.085 [0.016, 0.153]
Heterogeneity: Tau^2 = 0; Chi^2 = 0.56, df = 1 (P = 0.45); I^2 = 0%
Test for overall effect: Z = 2.43 (P = 0.02)
Test for subgroup differences: Chi^2 = 0.56, df = 1 (P = 0.45)
Correlation IV, Random, 95% CI
−0.2 −0.1 0 0.1 0.2

Figure E9

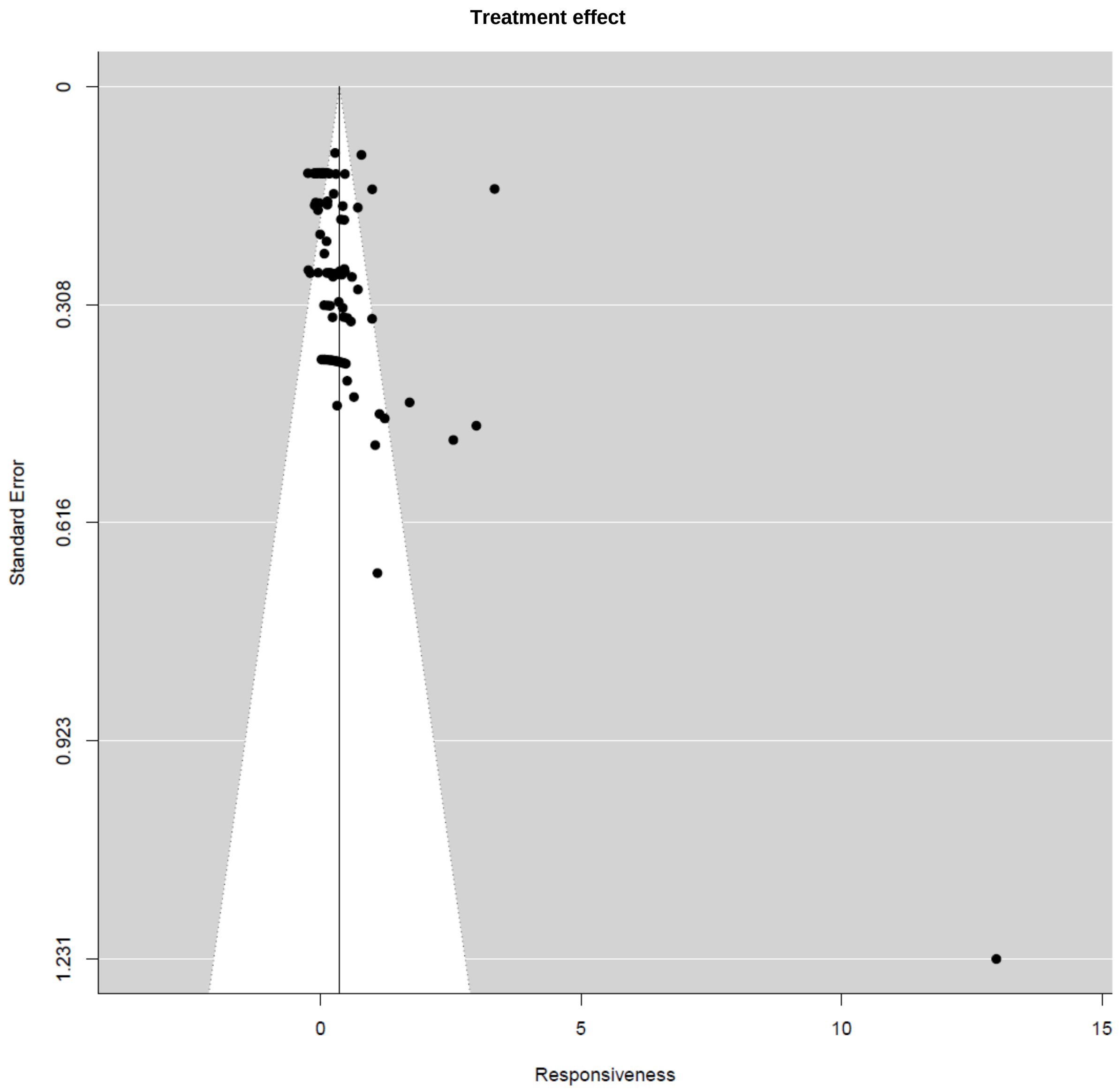

Treatment effect
0
0.308
0.616
0.923
1.231
Standard Error
0
5
10
15
Responsiveness

Figure E10a

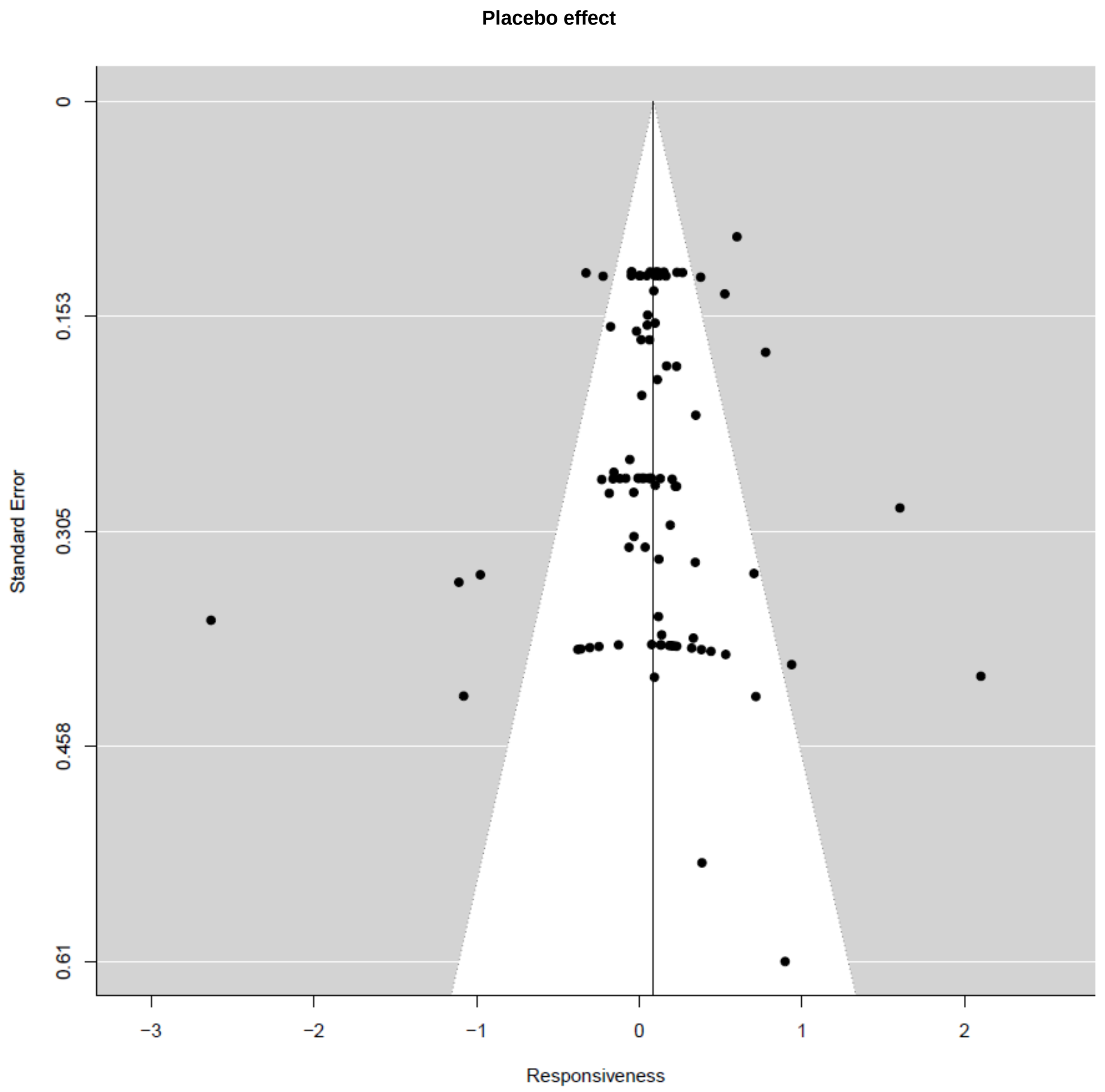
Placebo effect
Standard Error
0
0.153
0.305
0.458
0.61
−3
−2
−1
0
1
2
Responsiveness

Figure E10b

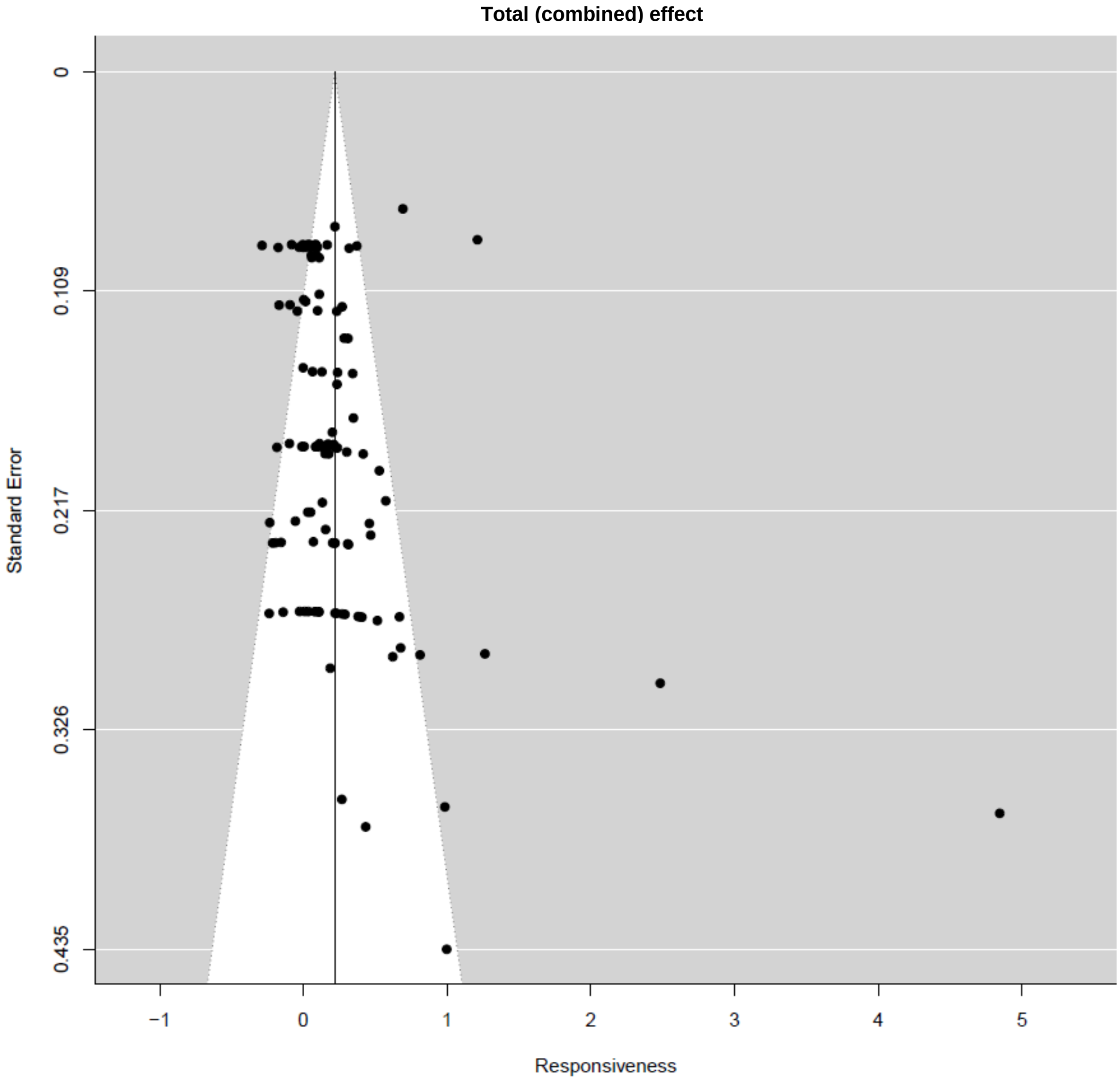
Total (combined) effect
Standard Error
0
0.109
0.217
0.326
0.435
−1
0
1
2
3
4
5
Responsiveness

Figure E10c

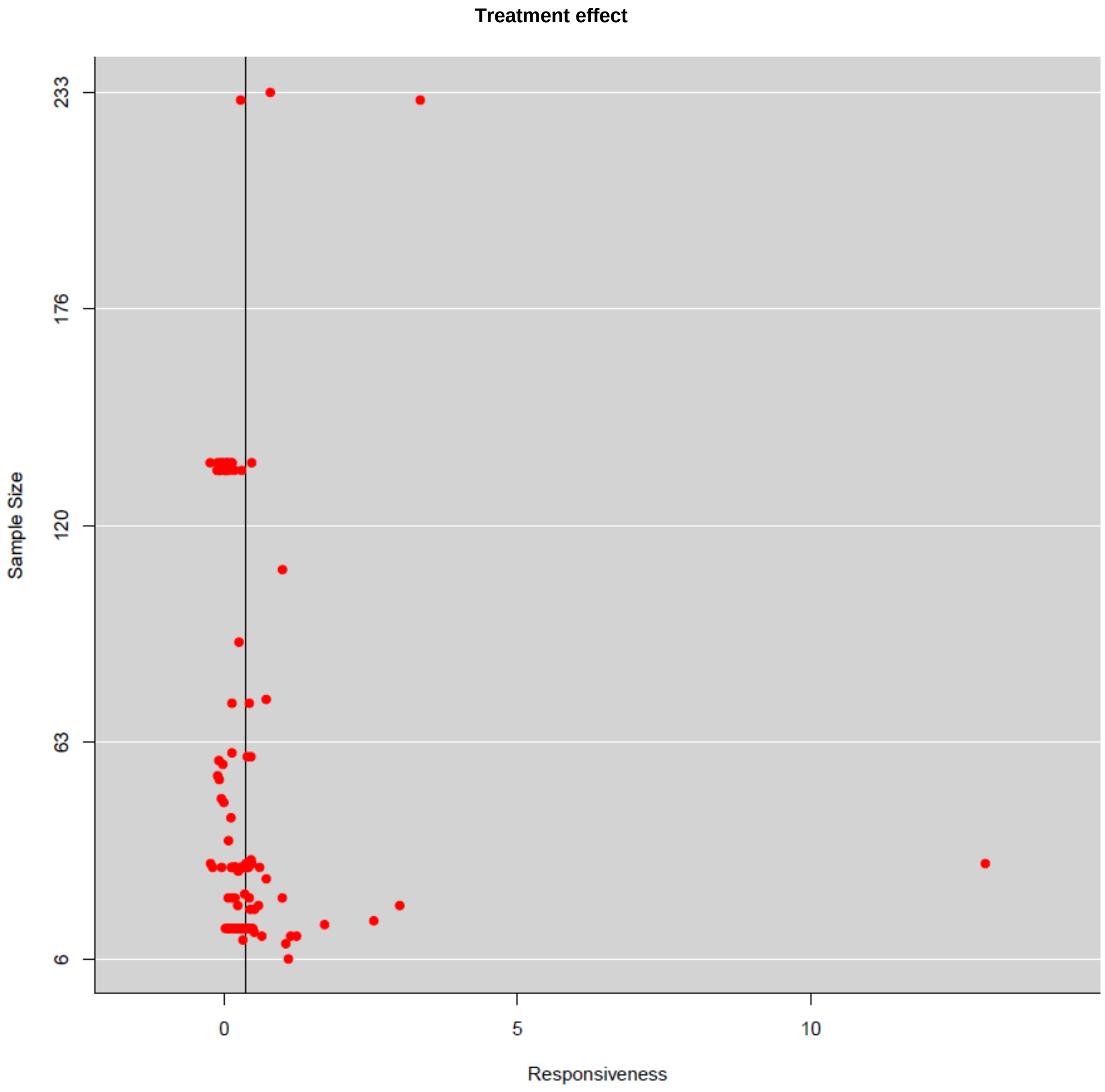

Treatment effect
Sample Size
233
176
120
63
6
0
5
10
Responsiveness

Figure E11a

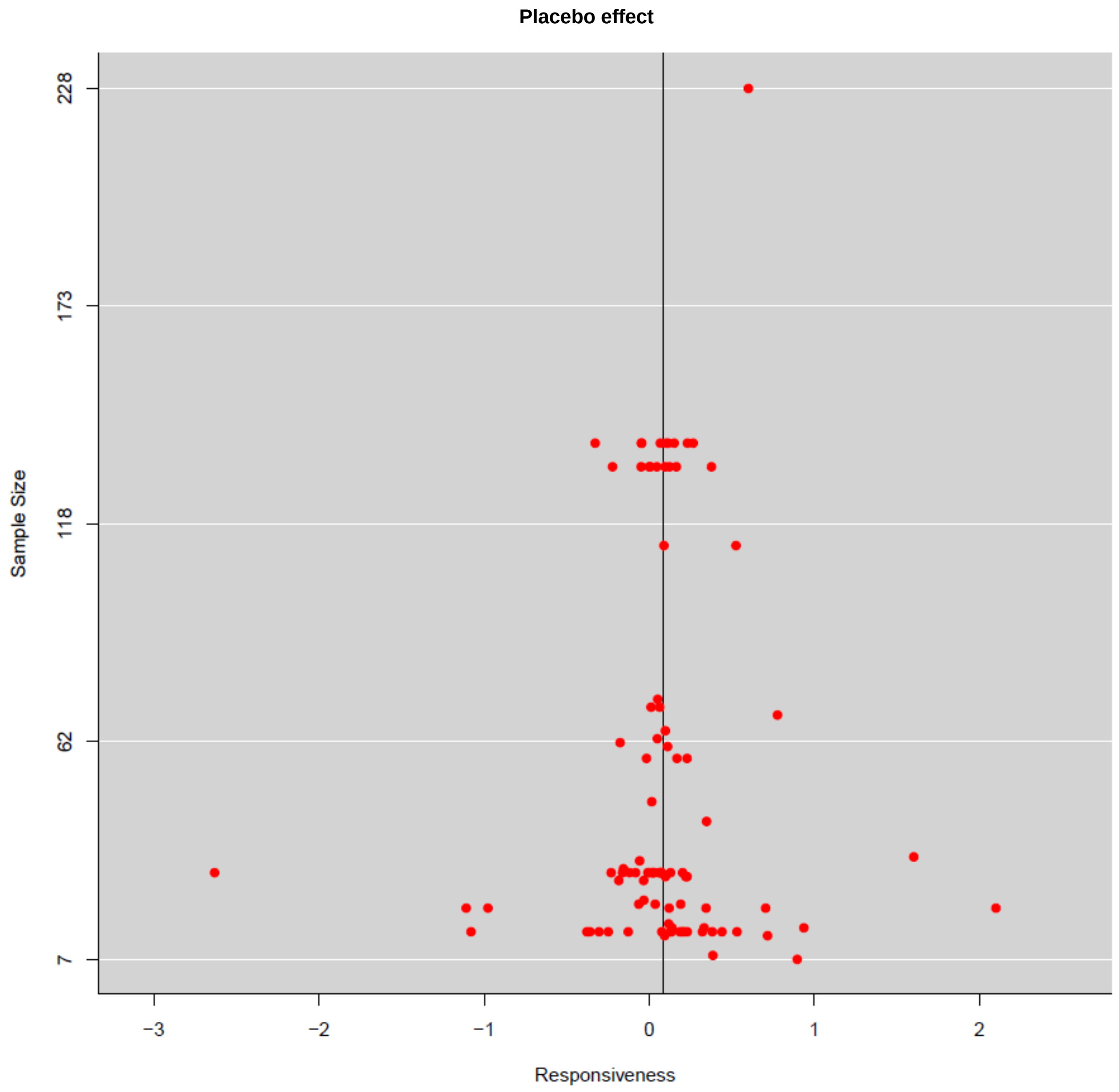
Placebo effect
Sample Size
228
173
118
62
7
−3
−2
−1
0
1
2
Responsiveness

Figure E11b

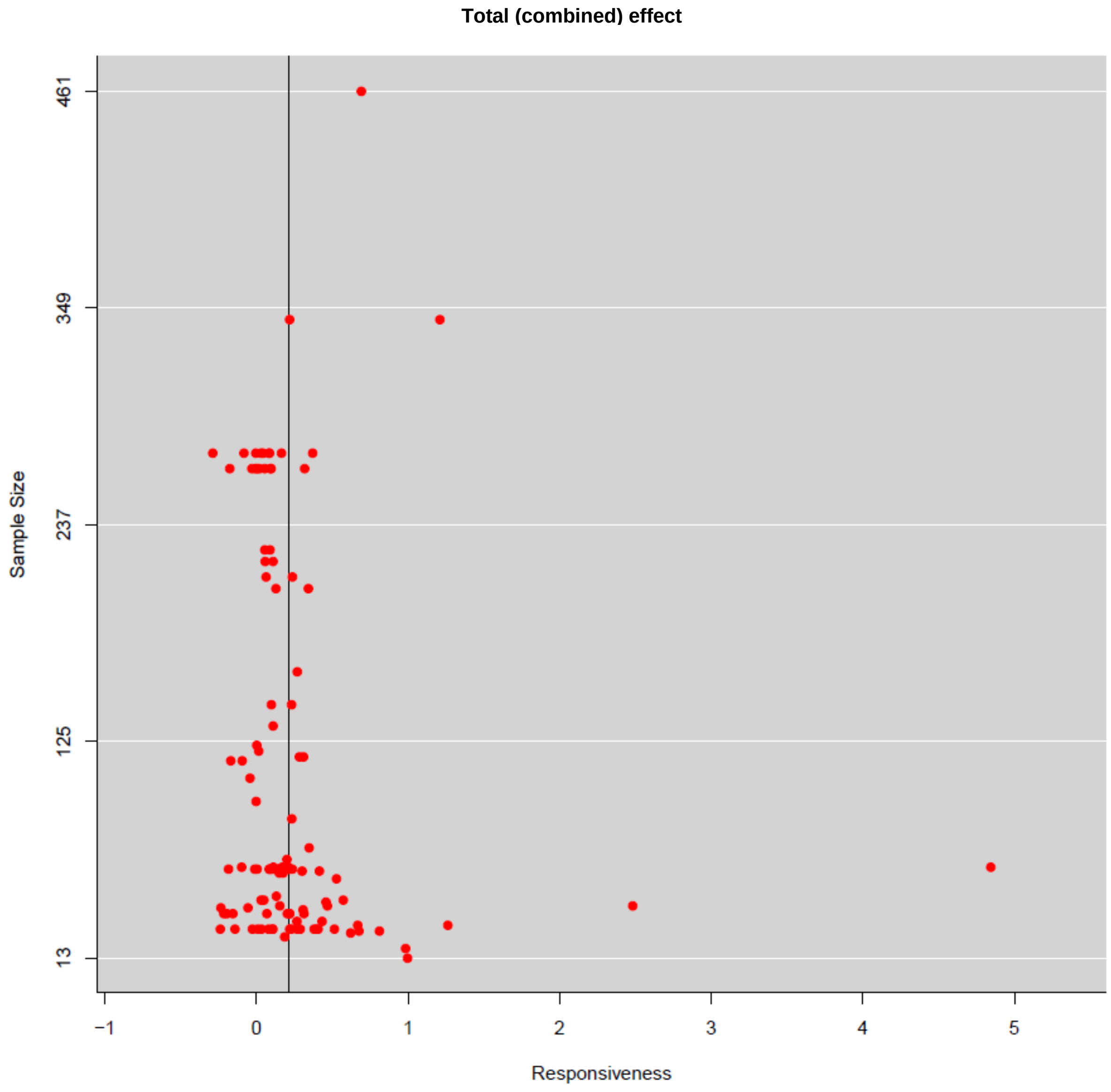
Total (combined) effect
Sample Size
461
349
237
125
13
−1
0
1
2
3
4
5
Responsiveness

Figure E11c

Treatment effect

Study & Questionnaire		Responsiveness [95% CI]
Adding Drobnic et al. (2005)	(k=1)	0.23 [−0.41; 0.87]
Adding Newall et al. (2005)	(k=2)	0.37 [−0.14; 0.89]
Adding Martinez−Garcia et al. (2006)	(k=3)	0.31 [−0.06; 0.67]
Adding Maa et al. (2007)	(k=4)	0.31 [−0.03; 0.65]
Adding Lavery et al. (2011)	(k=5)	0.35 [0.07; 0.64]
Adding Liaw et al. (2011)	(k=6)	0.37 [0.11; 0.64]
Adding Hernando et al. (2012)	(k=7)	0.30 [0.07; 0.53]
Adding Mandal et al. (2012)	(k=8)	0.35 [0.12; 0.57]
Adding Mandal et al. (2012)	(k=9)	0.41 [0.18; 0.64]
Adding Nicolson et al. (2012)	(k=10)	0.42 [0.21; 0.62]
Adding Nicolson et al. (2012)	(k=11)	0.69 [0.29; 1.09]
Adding Bilton et al. (2013)	(k=12)	0.98 [0.09; 1.86]
Adding Bilton et al. (2013)	(k=13)	0.92 [0.15; 1.69]
Adding Diego et al. (2013)	(k=14)	1.03 [0.28; 1.77]
Adding Serisier et al. (2013) (ORBIT−2)	(k=15)	0.97 [0.27; 1.68]
Adding Serisier et al. (2013) (BLESS)	(k=16)	0.94 [0.29; 1.58]
Adding Serisier et al. (2013) (BLESS)	(k=17)	0.90 [0.31; 1.49]
Adding Stockley et al. (2013)	(k=18)	0.87 [0.31; 1.44]
Adding Bilton et al. (2014)	(k=19)	0.86 [0.38; 1.34]
Adding Haworth et al. (2014) (Wk 12)	(k=20)	0.82 [0.37; 1.28]
Adding Haworth et al. (2014) (Wk 26)	(k=21)	0.80 [0.37; 1.23]
Adding Lee et al. (2014)	(k=22)	0.77 [0.36; 1.18]
Adding Mandal et al. (2014)	(k=23)	0.75 [0.35; 1.15]
Adding Quittner et al. (2014)	(k=24)	0.72 [0.35; 1.10]
Adding Liu et al. (2014)	(k=25)	0.73 [0.37; 1.10]
Adding Quittner et al. (2015) (RS, AIR BX1)	(k=26)	0.71 [0.37; 1.06]
Adding Quittner et al. (2015) (PF, AIR BX1)	(k=27)	0.68 [0.35; 1.01]
Adding Quittner et al. (2015) (Vit, AIR BX1)	(k=28)	0.65 [0.34; 0.97]
Adding Quittner et al. (2015) (RF, AIR BX1)	(k=29)	0.63 [0.32; 0.93]
Adding Quittner et al. (2015) (HP, AIR BX1)	(k=30)	0.60 [0.31; 0.89]
Adding Quittner et al. (2015) (EF, AIR BX1)	(k=31)	0.58 [0.30; 0.86]
Adding Quittner et al. (2015) (SF, AIR BX1)	(k=32)	0.56 [0.29; 0.83]
Adding Quittner et al. (2015) (TB, AIR BX1)	(k=33)	0.54 [0.28; 0.80]
Adding Quittner et al. (2015) (AIR BX1)	(k=34)	0.52 [0.27; 0.77]
Adding Quittner et al. (2015) (RS, AIR BX2)	(k=35)	0.52 [0.28; 0.76]
Adding Quittner et al. (2015) (PF, AIR BX2)	(k=36)	0.50 [0.27; 0.73]
Adding Quittner et al. (2015) (Vit, AIR BX2)	(k=37)	0.48 [0.26; 0.71]
Adding Quittner et al. (2015) (RF, AIR BX2)	(k=38)	0.46 [0.24; 0.68]
Adding Quittner et al. (2015) (HP, AIR BX2)	(k=39)	0.45 [0.24; 0.66]
Adding Quittner et al. (2015) (EF, AIR BX2)	(k=40)	0.44 [0.23; 0.64]
Adding Quittner et al. (2015) (SF, AIR BX2)	(k=41)	0.43 [0.23; 0.63]
Adding Quittner et al. (2015) (TB, AIR BX2)	(k=42)	0.41 [0.21; 0.60]
Adding Quittner et al. (2015) (AIR BX2)	(k=43)	0.40 [0.21; 0.59]
Adding Zanini et al. (2015)	(k=44)	0.42 [0.23; 0.60]
Adding Abdelhalim et al. (2016)	(k=45)	0.44 [0.25; 0.62]
Adding Dona et al. (2018) (RF, 3mths)	(k=46)	0.43 [0.24; 0.61]
Adding Dona et al. (2018) (RF, 6mths)	(k=47)	0.42 [0.24; 0.61]
Adding Dona et al. (2018) (Vit, 3mths)	(k=48)	0.42 [0.24; 0.61]
Adding Dona et al. (2018) (Vit, 6mths)	(k=49)	0.42 [0.24; 0.60]
Adding Dona et al. (2018) (EF, 3mths)	(k=50)	0.42 [0.25; 0.60]
Adding Dona et al. (2018) (EF, 6mths)	(k=51)	0.42 [0.24; 0.60]
Adding Dona et al. (2018) (SF, 3mths)	(k=52)	0.42 [0.24; 0.59]
Adding Dona et al. (2018) (SF, 6mths)	(k=53)	0.41 [0.24; 0.58]
Adding Dona et al. (2018) (TB, 3 mths)	(k=54)	0.41 [0.24; 0.58]
Adding Dona et al. (2018) (TB, 6mths)	(k=55)	0.41 [0.24; 0.58]
Adding Dona et al. (2018) (HP, 3mths)	(k=56)	0.40 [0.23; 0.57]
Adding Dona et al. (2018) (HP, 6mths)	(k=57)	0.40 [0.23; 0.56]
Adding Dona et al. (2018) (RS, 3mths)	(k=58)	0.40 [0.24; 0.56]
Adding Dona et al. (2018) (RS, 6mths)	(k=59)	0.40 [0.24; 0.56]
Adding Olveira et al. (2016) (PF, 3mths)	(k=60)	0.40 [0.24; 0.56]
Adding Olveira et al. (2016) (PF, 6mths)	(k=61)	0.40 [0.24; 0.56]
Adding Ailiyaer et al. (2018)	(k=62)	0.40 [0.24; 0.56]
Adding Munoz et al. (2018)	(k=63)	0.40 [0.25; 0.56]
Adding Munoz et al. (2018)	(k=64)	0.40 [0.25; 0.56]
Adding Ozalp et al. (2019)	(k=65)	0.40 [0.25; 0.56]
Adding Watz et al. (2019)	(k=66)	0.40 [0.24; 0.55]
Adding Atalay et al. (2019)	(k=67)	0.40 [0.25; 0.55]
Adding Zhong et al. (2020)	(k=68)	0.40 [0.26; 0.55]
Adding Brockwell et al. (2020) (12 months)	(k=69)	0.40 [0.25; 0.54]
Adding Brockwell et al. (2020) (Index value, 3 months)	(k=70)	0.39 [0.25; 0.54]
Adding Brockwell et al. (2020) (Index value, 6 months)	(k=71)	0.38 [0.24; 0.53]
Adding Brockwell et al. (2020) (Index value, 9 months)	(k=72)	0.38 [0.23; 0.52]
Adding Brockwell et al. (2020) (Index value, 12 months)	(k=73)	0.37 [0.23; 0.51]
Adding Brockwell et al. (2020) (QALY, 12 months)	(k=74)	0.36 [0.22; 0.50]
Adding Hester et al. (2020) (PF, 2 weeks)	(k=75)	0.36 [0.22; 0.49]
Adding Hester et al. (2020) (RF, 2 weeks)	(k=76)	0.36 [0.22; 0.49]
Adding Hester et al. (2020) (Vit, 2 weeks)	(k=77)	0.36 [0.22; 0.49]
Adding Hester et al. (2020) (EF, 2 weeks)	(k=78)	0.36 [0.22; 0.49]
Adding Hester et al. (2020) (SF, 2 weeks)	(k=79)	0.36 [0.22; 0.49]
Adding Hester et al. (2020) (TB, 2 weeks)	(k=80)	0.35 [0.22; 0.48]
Adding Hester et al. (2020) (HP, 2 weeks)	(k=81)	0.35 [0.22; 0.48]
Adding Hester et al. (2020) (RS, 2 weeks)	(k=82)	0.36 [0.23; 0.48]
Adding Hester et al. (2020) (Index value, 2 weeks)	(k=83)	0.36 [0.23; 0.48]
Adding Hester et al. (2020) (VAS, 2 weeks)	(k=84)	0.36 [0.23; 0.48]
Adding Hester et al. (2020) (PF, 12 weeks)	(k=85)	0.35 [0.23; 0.47]
Adding Hester et al. (2020) (RF, 12 weeks)	(k=86)	0.35 [0.22; 0.47]
Adding Hester et al. (2020) (Vit, 12 weeks)	(k=87)	0.34 [0.22; 0.46]
Adding Hester et al. (2020) (EF, 12 weeks)	(k=88)	0.34 [0.22; 0.46]
Adding Hester et al. (2020) (SF, 12 weeks)	(k=89)	0.34 [0.22; 0.46]
Adding Hester et al. (2020) (TB, 12 weeks)	(k=90)	0.34 [0.22; 0.45]
Adding Hester et al. (2020) (HP, 12 weeks)	(k=91)	0.33 [0.22; 0.45]
Adding Hester et al. (2020) (RS, 12 weeks)	(k=92)	0.33 [0.22; 0.45]
Adding Hester et al. (2020) (12 weeks)	(k=93)	0.33 [0.22; 0.45]
Adding Hester et al. (2020) (Index value, 12 weeks)	(k=94)	0.36 [0.24; 0.49]
Adding Hester et al. (2020) (VAS, 12 weeks)	(k=95)	0.36 [0.23; 0.48]
Adding Sliwinski et al. (2019)	(k=96)	0.36 [0.24; 0.48]
**Random effects model**		**0.36 [0.24; 0.48]**

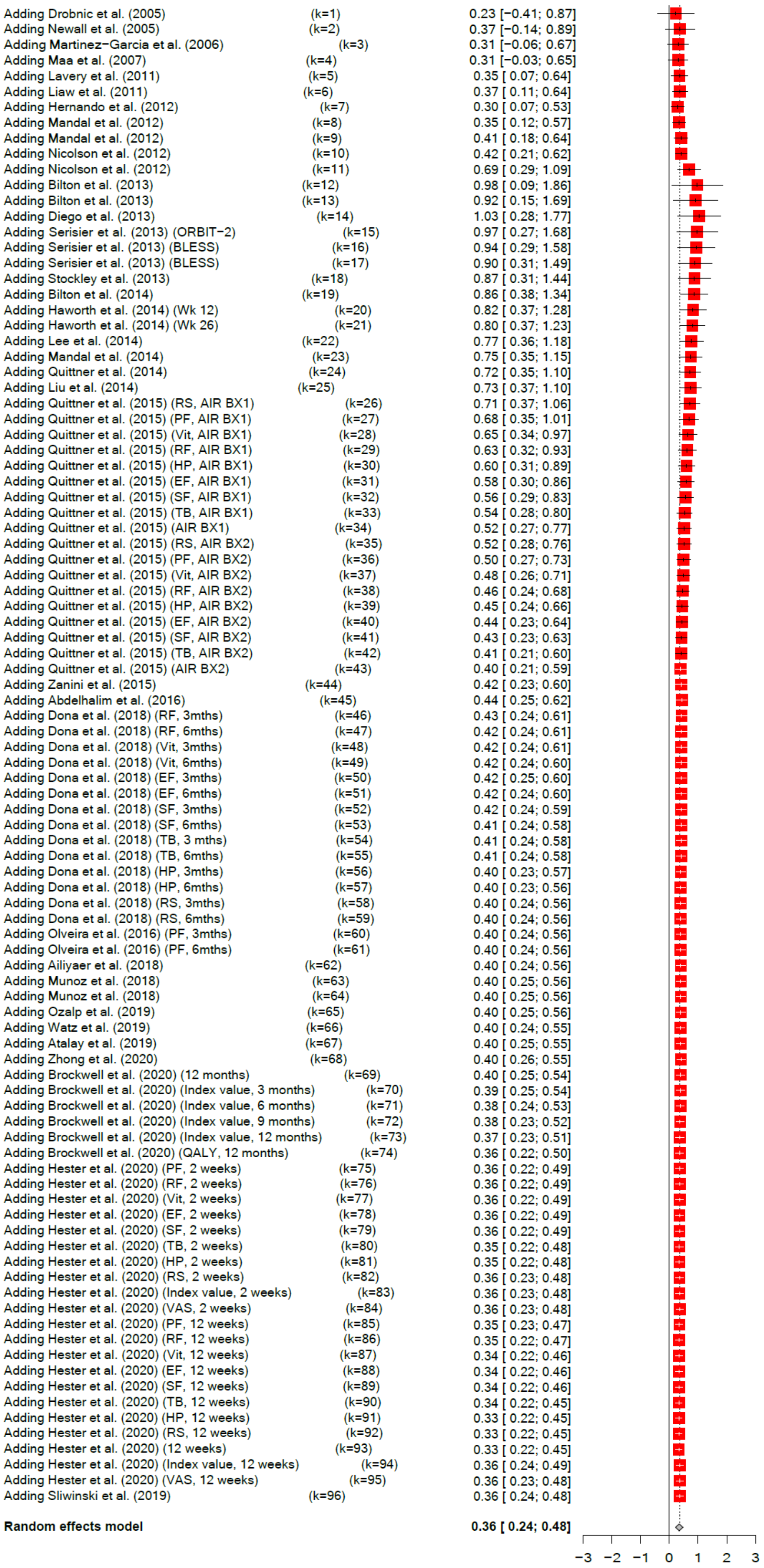

Figure E12a

EF, emotional functioning; ER, role emotional; GH, general health; HP, health perceptions; MH, mental health; RS, respiratory symptoms; Pain, bodily pain; PF, physical functioning; PR, role physical; RF, role functioning; SF, social functioning; TB, treatment burden; Vit, vitality.

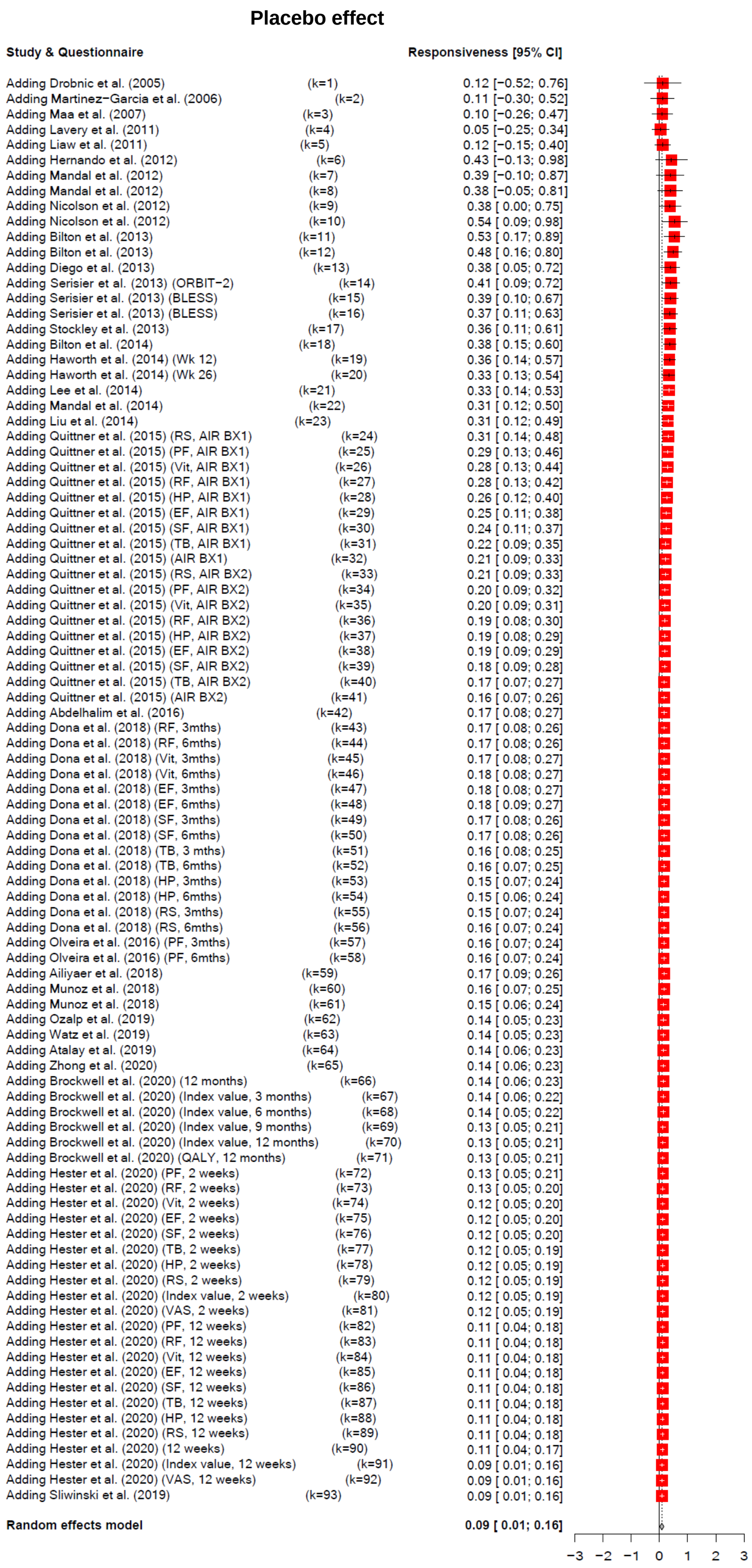

Figure E12b

EF, emotional functioning; ER, role emotional; GH, general health; HP, health perceptions; MH, mental health; RS, respiratory symptoms; Pain, bodily pain; PF, physical functioning; PR, role physical; RF, role functioning; SF, social functioning; TB, treatment burden; Vit, vitality.

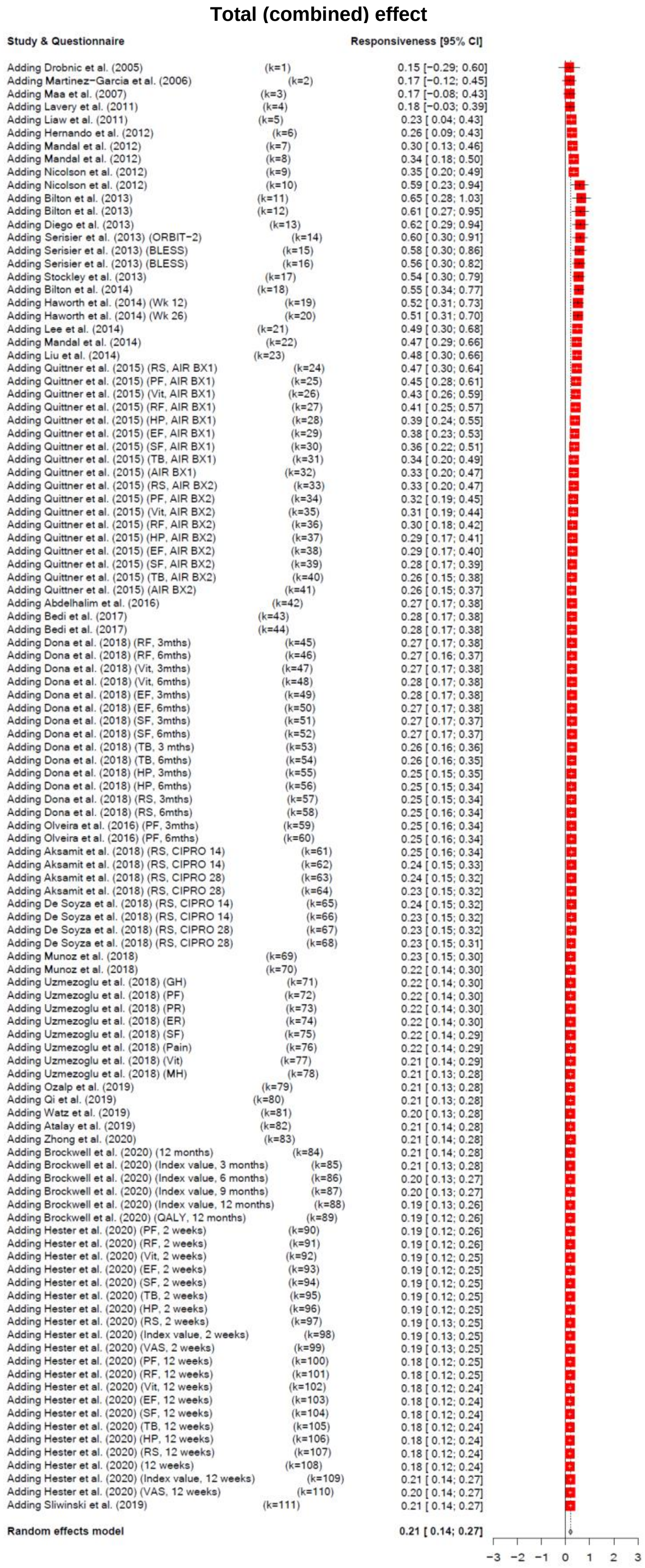

Figure E12c

EF, emotional functioning; ER, role emotional; GH, general health; HP, health perceptions; MH, mental health; RS, respiratory symptoms; Pain, bodily pain; PF, physical functioning; PR, role physical; RF, role functioning; SF, social functioning; TB, treatment burden; Vit, vitality

References

ABD-ELAZEEM, A., SADEK, S. H. & ATTIA, N. M. 2018. COPD-bronchiectasis overlap syndrome: Are there clinical, physiological and radiological synergistic effects? *Egypt J Radiol Nucl Med,* 49**,** 23-28.

AILIYAER, Y., WANG, X., ZHANG, Y., LI, C., LI, T., QI, Q. & LI, Y. 2018. A Prospective Trial of Nebulized Amikacin in the Treatment of Bronchiectasis Exacerbation. *Respiration,* 95**,** 327-333.

ALCARAZ-SERRANO, V., GIMENO-SANTOS, E., SCIOSCIA, G., GABARRUS, A., NAVARRO, A., HERRERO-CORTINA, B., AMARO, R., FERNANDEZ-BARAT, L. & TORRES, A. 2020. Association between physical activity and risk of hospitalisation in bronchiectasis. *Eur Respir J*, 55.

ALIBERTI, S., LONNI, S., DORE, S., MCDONNELL, M. J., GOEMINNE, P. C., DIMAKOU, K., FARDON, T. C., RUTHERFORD, R., PESCI, A., RESTREPO, M. I., SOTGIU, G. & CHALMERS, J. D. 2016. Clinical phenotypes in adult patients with bronchiectasis. *Eur Respir J,* 47**,** 1113-1122.

ARAUJO, D., SHTEINBERG, M., ALIBERTI, S. A.-O., GOEMINNE, P. C., HILL, A. T., FARDON, T. C., OBRADOVIC, D., STONE, G., TRAUTMANN, M., DAVIS, A., DIMAKOU, K., POLVERINO, E., DE SOYZA, A. A.-O., MCDONNELL, M. J. & CHALMERS, J. D. 2018. The independent contribution of *Pseudomonas aeruginosa* infection to long-term clinical outcomes in bronchiectasis. *Eur Respir J,* 51**,** 1701953.

ARTARAZ, A., CRICHTON, M. L., FINCH, S., ABO-LEYAH, H., GOEMINNE, P., ALIBERTI, S., FARDON, T. & CHALMERS, J. D. 2020. Development and initial validation of the bronchiectasis exacerbation and symptom tool (BEST). *Respir Res,* 21, 18.

ASAKURA, T., MORIMOTO, K., ITO, A., SUZUKI, S., MORINO, E., OSHITANI, Y., NAKAGAWA, T., YAGI, K., KADOWAKI, T., SAITO, F., HASE, I., FURUUCHI, K., OKAMORI, S., KUSUMOTO, T., HIRABAYASHI, R., OGAWA, T., KAMATA, H., NAMKOONG, H., TAKASAKI, J., FUJITA, M., OGAWA, K., KITADA, S., ISHIDA, T., KURASHIMA, A., ISHII, M., MIYATA, H., ATO, M. & HASEGAWA, N. 2020. Etiology and health-related quality of life in non-cystic fibrosis bronchiectasis and nontuberculous mycobacterial pulmonary disease: the first analysis of the Japanese nontuberculous mycobacteriosis-bronchiectasis registry. *Am J Respir Crit Care Med,* 201.

ATALAY, O. T., YILMAZ, A., ALTINISIK, G., CENGIZ, B., TASKIN, H., YALMAN, A. & KIZMAZ, E. 2019. Comparison of the effect of aerobic exercise with whole body vibration in patients with bronchiectasis: Single blind randomized controlled study. *Turk Thorac J,* 20, S90.

BALTEANU, M., TRAISTARU, R., CIORAN, N., TANASESCU, M. & POSTOLACHE, P. 2017. P192 An eighth week pulmonary rehabilitation in patients with non-cystic fibrosis bronchiectasis - observational study. *Chest,* 151**,** A91.

BARTLEY, J., GARRETT, J., CAMARGO, C. A., JR., SCRAGG, R., VANDAL, A., SISK, R., MILNE, D., TAI, R., JEON, G., CURSONS, R. & WONG, C. 2018. Vitamin D(3) supplementation in adults with bronchiectasis: A pilot study. *Chron Respir Dis,* 15**,** 384-392.

BASAVARAJ, A., SEGAL, L. N., SCOTT, A. S., KITNER, S., HARRIS, D., ADDRIZZO-HARRIS, D. J. & KAMELHAR, D. 2019. Clinical phenotypes among patients with bronchiectasis. *Am J Respir Crit Care Med*, 199.

BATCHELOR, H., BRILL, S., BROWN, J. S. & HURST, J. R. 2011. P248 Associations of depression, fatigue and quality of life in adult non-CF bronchiectasis. *Thorax,* 66**,** A168.

BEDI, P., CHALMERS, J. D., GRAHAM, C., CLARKE, A., DONALDSON, S., DOHERTY, C., GOVAN, J. R. W., DAVIDSON, D. J., ROSSI, A. G. & HILL, A. T. 2017. A Randomized Controlled Trial of Atorvastatin in Patients With Bronchiectasis Infected With *Pseudomonas Aeruginosa*: A Proof of Concept Study. *Chest,* 152**,** 368-378.

BEKIR, M., OLGUN YILDIZELI, S., KOCAKAYA, D., ERYUKSEL, E. & BAGCI CEYHAN, B. 2017. Health related quality of life among adult patients with non cf bronchiectasis:the role of psychological status and disease severity. *Eur Respir J,* 50**,** 715.

BELLELLI, G., CHALMERS, J. D., SOTGIU, G., DORE, S., MCDONNELL, M. J., GOEMINNE, P. C., DIMAKOU, K., SKRBIC, D., LOMBI, A., PANE, F., OBRADOVIC, D., FARDON, T. C., RUTHERFORD, R. M., PESCI, A. & ALIBERTI, S. 2016. Characterization of bronchiectasis in the elderly. *Respir Med,* 119**,** 13-19.

BILTON, D., DAVISKAS, E., ANDERSON, S. D., KOLBE, J., KING, G., STIRLING, R. G., THOMPSON, B. R., MILNE, D. & CHARLTON, B. 2013. Phase 3 randomized study of the efficacy and safety

of inhaled dry powder mannitol for the symptomatic treatment of non-cystic fibrosis bronchiectasis. *Chest,* 144**,** 215-225.

BILTON, D., TINO, G., BARKER, A. F., CHAMBERS, D. C., DE SOYZA, A., DUPONT, L. J. A., DOCHARTAIGH, C., VAN HAREN, E. H. J., VIDAL, L. O., WELTE, T., FOX, H. G., WU, J. & CHARLTON, B. 2014. Inhaled mannitol for non-cystic fibrosis bronchiectasis: a randomised, controlled trial. *Thorax,* 69**,** 1073.

BIRRING, S. S., PRUDON, B., CARR, A. J., SINGH, S. J., MORGAN, M. D. & PAVORD, I. D. 2003. Development of a symptom specific health status measure for patients with chronic cough: Leicester Cough Questionnaire (LCQ). *Thorax,* 58**,** 339-43.

BONDARENKO, J., BUTTON, BM., LEE, AL., NICOLSON, CH. 2019. The Clinical Impact of Self-Reported Symptoms of Chronic Rhinosinusitis in Adults with Bronchiectasis. Abstracts: 3rd World Bronchiectasis Conference *Chronic Obstr Pulm Dis,* 5**,** 307.

BORENSTEIN, M., HEDGES, L., HIGGINS, J. & ROTHSTEIN, H. 2011. *An Introduction to Meta-Analysis,* Chichester, UK, John Wiley & Sons.

BRADLEY, J. M., WILSON, J. J., HAYES, K., KENT, L., MCDONOUGH, S., TULLY, M. A., BRADBURY, I., KIRK, A., COSGROVE, D., CONVERY, R., KELLY, M., ELBORN, J. S. & O'NEILL, B. 2015. Sedentary behaviour and physical activity in bronchiectasis: a cross-sectional study. *BMC Pulm Med,* 15**,** 61.

BRILL, S., PATEL, A., SINGH, R., MACKAY AJ, BROWN, J. & HURST, J. 2015. Lung function, symptoms and inflammation during exacerbations of non-cystic fibrosis bronchiectasis: a prospective observational cohort study. *Respir Res,* 16**,** 16.

BROCKWELL, C., STOCKL, A., CLARK, A., BARTON, G., PASTEUR, M., FLEETCROFT, R., HILL, J. & WILSON, A. M. 2020. Randomised controlled trial of the effect, cost and acceptability of a bronchiectasis self-management intervention. *Chron Respir Dis*, 17.

BROWN, J., BRADLEY, J., COPELAND, F., CARROLL, M., CRICHTON, M., DUCKERS, J., HAWORTH, C., FLOTO, R. A., HILL, A. T., LOEBINGER, M., WILSON, R., HURST, J., COOKSON, W., WINSTANLEY, C., MCGUIRE, A., MCNALLY, R., MAWSON, P., KELLEHER, P., DENNING, D., NAVARATNAM, V., HUBBARD, R., KELLY, M., STEER, J., SULLIVAN, A., GATHERAL, T., WALKER, P., ELBORN, J. S., CHALMERS, J. D. & DE SOYZA, A. 2019. Convergent validity of bronchiectasis quality of life tools in the bronch-UK registry. *Thorax,* 74, A248-A249.

BUCHHOLZ, I., JANSSEN, M. F., KOHLMANN, T. & FENG, Y. S. 2018. A Systematic Review of Studies Comparing the Measurement Properties of the Three-Level and Five-Level Versions of the EQ-5D. *Pharmacoeconomics,* 36**,** 645-661.

BULCUN, E., ARSLAN, M., EKICI, A. & EKICI, M. 2015. Quality of Life and Bronchial Hyper-Responsiveness in Subjects With Bronchiectasis: Validation of the Seattle Obstructive Lung Disease Questionnaire in Bronchiectasis. *Respir Care,* 60**,** 1616-1623.

CAKMAK, A., INAL-INCE, D., SONBAHAR-ULU, H., BOZDEMIR-OZEL, C., OZALP, O., CALIK-KUTUKCU, E., SAGLAM, M., VARDAR-YAGLI, N., ARIKAN, H., SELCUK, Z. T. & COPLU, L. 2020. Physical activity of patients with bronchiectasis compared with healthy counterparts: A cross-sectional study. *Heart Lung,* 49**,** 99-104.

CARTLIDGE, M. K., SMITH, M. P., BEDI, P., DONALDSON, S., CLARKE, A., MANTOANI, L. C., RABINOVICH, R. A., ROSSI, A. G. & HILL, A. T. 2018. Validation of the Incremental Shuttle Walk Test as a Clinical End Point in Bronchiectasis. *Chest,* 154**,** 1321-1329.

CHALMERS, J., ALIBERTI, S., FILONENKO, A., SHTEINBERG, M., GOEMINNE, P., HILL, A., FARDON, T., OBRADOVIĆ, D., GERLINGER, C., SOTGIU, G., OPERSCHALL, E., RUTHERFORD, R., DIMAKOU, K., POLVERINO, E., SOYZA, A. & MCDONNELL, M. 2018. Characterisation of the "Frequent Exacerbator Phenotype" in Bronchiectasis. *Am J Respir Crit Care Med,* 197**,** 1410-1420.

CHALMERS, J. D., GOEMINNE, P., ALIBERTI, S., MCDONNELL, M. J., LONNI, S., DAVIDSON, J., POPPELWELL, L., SALIH, W., PESCI, A., DUPONT, L. J., FARDON, T. C., DE SOYZA, A. & HILL, A. T. 2014. The bronchiectasis severity index. An international derivation and validation study. *Am J Respi Crit Care Med,* 189**,** 576-585.

CHALMERS, J. D., MOFFITT, K. L., SUAREZ-CUARTIN, G., SIBILA, O., FINCH, S., FURRIE, E., DICKER, A., WROBEL, K., ELBORN, J. S., WALKER, B., MARTIN, S. L., MARSHALL, S. E., HUANG, J. T. & FARDON, T. C. 2017. Neutrophil Elastase Activity Is Associated with Exacerbations and Lung Function Decline in Bronchiectasis. *Am J Respir Crit Care Med,* 195**,** 1384-1393.

CHAN, S. L., CHAN-YEUNG, M. M., OOI, G. C., LAM, C. L., CHEUNG, T. F., LAM, W. K. & TSANG, K. W. 2002. Validation of the Hong Kong Chinese version of the St. George Respiratory Questionnaire in patients with bronchiectasis. *Chest,* 122**,** 2030-2037.

CHAURASIA, S., REDDY, A., HARAN, A. & CHAWLA, P. 2017. Quality of life in non cystic fibrosis bronchiectasis(NCFB): predictors and correlation with other outcome parameters. *Eur Respir J,* 50**,** PA3698.

CHOI, H., LEE, H. & OH, Y. 2020. Clinical characteristics of patients with bronchiectasis and comorbid asthma: The KMBARC registry data. *Am J Respir Crit Care Med*, 201.

CORDOVA-RIVERA, L., GIBSON, P. G., GARDINER, P. A., HILES, S. A. & MCDONALD, V. M. 2019. Extrapulmonary associations of health status in severe asthma and bronchiectasis: Comorbidities and functional outcomes. *Respir Med,* 154**,** 93-101.

DAL CORSO, S., JOSÉ, A., HOLLAND, A. E., SELMAN, J. P. R., CASTRO, R. A. S., DE CAMARGO, A. A., DE OLIVEIRA, C. S., FREITAS, T. P., ATHANASIO, R., RACHED, S. Z., CUKIER, A. & STELMACH, R. 2017. Home-based pulmonary rehabilitation in patients with bronchiectasis: a randomized controlled trial. *Eur Respir J,* 50**,** OA4668.

DE CAMARGO, C. O., JOSE, A., LUPPO, A., DE CAMARGO, A. A., ATHANAZIO, R. A., RACHED, S. Z., QUITTNER, A. L., STELMACH, R. & DAL CORSO, S. 2020. Quality of Life Questionnaire-Bronchiectasis: a study of the psychometric properties of the Brazilian Portuguese version. *Clin Rehabil,* 34, 960-970.

DHAR, R., SINGH, S., TALWAR, D., MOHAN, M., TRIPATHI, S. K., SWARNAKAR, R., TRIVEDI, S., RAJAGOPALA, S., D'SOUZA, G., PADMANABHAN, A., BABURAO, A., MAHESH, P. A., GHEWADE, B., NAIR, G., JINDAL, A., JAYADEVAPPA, G. D. H., SAWHNEY, H., SARMAH, K. R., SAHA, K., ANANTHARAJ, S., KHANNA, A., GAMI, S., SHAH, A. A., SHAH, A. A., DUTT, N., GARG, H., VYAS, S., VENUGOPAL, K., PRASAD, R., ALEEMUDDIN, N. M., KARMAKAR, S., SINGH, V., JINDAL, S. K., SHARMA, S., PRAJAPAT, D., CHANDRASHEKARIA, S., MCDONNELL, M. J., MISHRA, A., RUTHERFORD, R., RAMANATHAN, R. P., GOEMINNE, P. C., VASUDEV, P., DIMAKOU, K., CRICHTON, M. L., JAYARAJ, B. S., KUNGWANI, R., DAS, A., SAWHNEY, M., POLVERINO, E., TORRES, A., GULECHA, N. S., SHTEINBERG, M., DE SOYZA, A., MANGALA, A., SHAH, P., CHAUHAN, N. K., JAJODIA, N., SINGHAL, A., BATRA, S., HASAN, A., LIMAYE, S., SALVI, S., ALIBERTI, S. & CHALMERS, J. D. 2019. Bronchiectasis in India: results from the European Multicentre Bronchiectasis Audit and Research Collaboration (EMBARC) and Respiratory Research Network of India Registry. *Lancet Glob Health,* 7**,** e1269-e1279.

DIEGO, A. D., MILARA, J., MARTINEZ-MORAGÓN, E., PALOP, M., LEÓN, M. & CORTIJO, J. 2013. Effects of long-term azithromycin therapy on airway oxidative stress markers in non-cystic fibrosis bronchiectasis. *Respirology,* 18**,** 1056-1062.

DIGGINS, B., GOOD, W., POOT, B., MAZE, M., DAWKINS, P., STROIL-SALAMA, E., MORGAN, L. & WONG, C. 2020. Bronchiectasis Severity and Quality of Life in New Zealand Vary According to Socioeconomic Factors and Ethnicity. *Respirology,* 25, 221.

DOÑA, E., OLVEIRA, C., PALENQUE, F. J., PORRAS, N., DORADO, A., MARTÍN-VALERO, R., GODOY, A. M., ESPILDORA, F. & OLVEIRA, G. 2018. Pulmonary Rehabilitation Only Versus With Nutritional Supplementation in Patients With Bronchiectasis: A RANDOMIZED CONTROLLED TRIAL. *J Cardiopulm Rehabil Prev,* 38**,** 411-418.

DOS REIS, L. V. T., MATEUS, S. P., SALLES, R. E. B., COSTA, W., SALLES, R. I. B., SALLES, R. I. B. & ALVES, R. L. R. 2018. Valuation of Health Status in Patients with Non-Fibrocystic Bronchiectasis Using the Questionnaire EQ-5D-3L. *American Thoracic Society***,** A1971-A1971.

DREW, J. C., CAREY, K. A., OLIVIER, K. N., RICOTTA, E., KWAN, J., ROBINSON, C., MACDONALD, S. D., FENNELLY, K. P. & PREVOTS, R. 2019. The risk of mortality among patients with bronchiectasis at a tertiary care center: Quality of life and 6MWD. *Am J Respir Crit Care Med*, 199.

DROBNIC, M. E., SUÑÉ, P., MONTORO, J. B., FERRER, A. & ORRIOLS, R. 2005. Inhaled tobramycin in non-cystic fibrosis patients with bronchiectasis and chronic bronchial infection with Pseudomonas aeruginosa. *Ann Pharmacother,* 39**,** 39-44.

DUDGEON, E., CRICHTON, M., SCOTT, G., FINCH, S., FARDON, T. C. & CHALMERS, J. D. 2017. A Quantitative and Qualitative Study to Understand Symptom Burden in Patients with Bronchiectasis. *American Thoracic Society***,** A1520-A1520.

EGGER, M., SMITH, G. D., SCHNEIDER, M. & MINDER, C. 1997. Bias in meta-analysis detected by a simple, graphical test. *BMJ,* 315**,** 629-634.

ESHED, I., MINSKI, I., KATZ, R., JONES, P. W. & PRIEL, I. E. 2007. Bronchiectasis: correlation of high-resolution CT findings with health-related quality of life. *Clin Radiol,* 62**,** 152-9.

EWEN, R., BARTEN-NEINER, G., DE ROUX, A., DIEL, R., MERTSCH, P., RADEMACHER, J., WELTE, T., ZURAWSKI, A. & RINGSHAUSEN, F. C. 2019. Nontuberculous mycobacteria in patients with bronchiectasis: Data from the German Bronchiectasis Registry PROGNOSIS. *Eur Respir J,* 54.

FELIU, A., RODRIGO-TROYANO, A., PEREA, L., GINER, J., CASTILLO, D., SÁNCHEZ-REUS, F., DE LA ROSA, D., MARTÍNEZ-GARCÍA, M. Á. & SIBILA VIDAL, O. 2018. Utility of CAT questionnaire in the diagnosis of bacterial colonization in Bronchiectasis. *Eur Respir J,* 52**,** PA5302.

FERRI, S., TAMMARO, S., RETUCCI, M., BELLOFIORE, A., SOLOPERTO, S., DI FRANCESCO, C., GELMINI, A., GRAMEGNA, A., ORI, M., BRAGHIROLI, A., BLASI, F. & ALIBERTI, S. 2020. Impact of cough on insomnia and sleep quality in bronchiectasis. *Eur Respir J,* 56.

FINCH, S. M., SHOEMARK, A., CRICHTON, M., LOEBINGER, M., POLVERINO, E., ALIBERTI, S., GOEMINNE, P., VENDRELL, M., SOYZA, A. D., DIMAKOU, K. & CHALMERS, J. D. 2017. Sex differences in reported quality of life in bronchiectasis: an analysis of the embarc registry. *Thorax,* 72**,** A28.

FLETCHER, KE., FRENCH, CT., IRWIN, RS., CORAPI, KM. & NORMAN, GR. 2010. A prospective global measure, the Punum Ladder, provides more valid assessments of quality of life than a retrospective transition measure. *J Clin Epidemiol,* 63, 1123-1131.

FRENCH, CT., IRWIN, RS., FLETCHER, KE. & ADAMS, TM. 2002. Evaluation of a cough-specific quality-of-life questionnaire. *Chest,* 121**,** 1123-31.

GALE, NS., DUCKERS, JM., MUNNERY, M., ENRIGHT, S., SHALE, DJ. 2010. Functional impairment in patients with bronchiectasis. *Thorax,* 65, A178.

GALINDO-PACHECO, LV., AMAYA-MEJIA, AS., O'FARRILL-ROMANILLOS, PM., DEL RIVERO-HERNANDEZ, LG., SEGURA-MENDEZ, NH. 2013. Quality of life in adults with variable common immunodeficiency and bronchiectasis. *Rev Alerg Mex,* 60(3), 123-128.

GALLI, B., SANTOS, D. O., MOROLI, R. G., RIBEIRO, M. A., OTTONI, I. E. F., BADDINI-MARTINEZ, J. A. & GASTALDI, A. C. 2019. Anxiety, depression, and cough and health related quality of life in bronchiectasis. *Eur Respir J,* 54.

GAO, Y.-H., GUAN, W.-J., XU, G., LIN, Z.-Y., TANG, Y., LIN, Z.-M., GAO, Y., LI, H.-M., ZHONG, N.-S., ZHANG, G.-J. & CHEN, R.-C. 2015. The Role of Viral Infection in Pulmonary Exacerbations of Bronchiectasis in Adults: A Prospective Study. *Chest,* 147**,** 1635-1643.

GAO, Y., GUAN, W., XU, G., LIN, Z., TANG, Y., LIN, Z., LI, H., GAO, Y., LUO, Q., ZHONG, N. & CHEN, R. 2014a. Sleep disturbances and health-related quality of life in adults with steady-state bronchiectasis. *PLoS One,* 9**,** e102970.

GAO, Y. H., GUAN, W. J., ZHU, Y. N., CHEN, R. C. & ZHANG, G. J. 2018. Anxiety and depression in adult outpatients with bronchiectasis: Associations with disease severity and health-related quality of life. *Clin Respir J,* 12**,** 1485-1494.

GAO, Y. H., ABO LEYAH, H., FINCH, S., LONERGAN, M., ALIBERTI, S., DE SOYZA, A., FARDON, T. C., TINO, G. & CHALMERS, J. D. 2020. Relationship between Symptoms, Exacerbations, and Treatment Response in Bronchiectasis. *Am J Respir Crit Care Med,* 201, 1499-1507.

GIRÓN MORENO, R. M., FERNANDES VASCONCELOS, G., CISNEROS, C., GÓMEZ-PUNTER, R. M., SEGRELLES CALVO, G. & ANCOCHEA, J. 2013. Trastornos psicológicos y calidad de vida en pacientes con bronquiectasias no relacionadas con fibrosis quística. *Arch Bronconeumol,* 49**,** 415-420.

GISSEL, T. N., HVASS, M., FLY, J., BORSTING, K., BIRRING, S. S., SPINOU, A. & WEINREICH, U. M. 2020. Translation and linguistic validation of the Bronchiectasis Health Questionnaire (BHQ) into Danish. *Dan Med J,* 67.

GOEMINNE, P. C., NAWROT, T. S., RUTTENS, D., SEYS, S. & DUPONT, L. J. 2014. Mortality in non-cystic fibrosis bronchiectasis: a prospective cohort analysis. *Respir Med,* 108**,** 287-96.

GUAN, W.-J., GAO, Y.-H., XU, G., LIN, Z.-Y., TANG, Y., LI, H.-M., LIN, Z.-M., ZHENG, J.-P., CHEN, R.-C. & ZHONG, N.-S. 2014. Capsaicin Cough Sensitivity and the Association with Clinical Parameters in Bronchiectasis. *PLoS One,* 9**,** e113057.

GUAN, W., YH, G., G, X., ZY, L., Y, T., HM, L., ZM, L., JP, Z., RC, C. & ZHONG, N. 2015a. Six-minute walk test in Chinese adults with clinically stable bronchiectasis: association with clinical indices and determinants. *Curr Med Resp Opin***,** 1-10.

GUAN, W. J., GAO, Y. H., XU, G., LIN, Z. Y., TANG, Y., LI, H. M., LIN, Z. M., ZHENG, J. P., CHEN, R.

C. & ZHONG, N. S. 2015c. Sputum bacteriology in steady-state bronchiectasis in Guangzhou, China. *Int J Tuberc Lung Dis,* 19**,** 610-9.
GUAN, W. J., JIANG, M., GAO, Y. H., LI, H. M., XU, G., ZHENG, J. P., CHEN, R. C. & ZHONG, N. S. 2016. Unsupervised learning technique identifies bronchiectasis phenotypes with distinct clinical characteristics. *Int J Tuberc Lung Dis,* 20**,** 402-10.
GUILEMANY, J. M., ANGRILL, J., ALOBID, I., CENTELLAS, S., PRADES, E., ROCA, J., PUJOLS, L., BERNAL-SPREKELSEN, M., PICADO, C. & MULLOL, J. 2009. United airways: the impact of chronic rhinosinusitis and nasal polyps in bronchiectasic patient's quality of life. *Allergy,* 64**,** 1524-9.
GUYATT, G. H., BERMAN, L. B., TOWNSEND, M., PUGSLEY, S. O. & CHAMBERS, L. W. 1987. A measure of quality of life for clinical trials in chronic lung disease. *Thorax,* 42**,** 773-8.
HALL, K., KUYS, S., SMITH, D. 2015. Sustained improvements in quality of life for non CF bronchiectasis following outpatient physiotherapy. *Respirology,* 20**,** 115.
HERNANDO, R., DROBNIC, M. E., CRUZ, M. J., FERRER, A., SUÑÉ, P., MONTORO, J. B. & ORRIOLS, R. 2012. Budesonide efficacy and safety in patients with bronchiectasis not due to cystic fibrosis. *Int J Clin Pharm,* 34**,** 644-50.
HERRERO-CORTINA, B., ALCARAZ, V., VILARÓ, J., TORRES, A. & POLVERINO, E. 2018a. Impact of Hypertonic Saline Solutions on Sputum Expectoration and Their Safety Profile in Patients with Bronchiectasis: A Randomized Crossover Trial. *J Aerosol Med Pulm Drug Deliv,* 31**,** 281-289.
HERRERO-CORTINA, B., MEMBRADO-LOPE, O., SAN MIGUEL-PAGOLA, M., FRANCÍN-GALLEGO, M., SAEZ-PEREZ, J. A. & POLVERINO, E. 2018b. Effects of a home programme of airway clearance techniques on adventitious respiratory sounds in patients with bronchiectasis: a randomised controlled trial. *Eur Respir J,* 52**,** PA349.
HERRERO-CORTINA, B., VILARÓ, J., MARTÍ, D., TORRES, A., SAN MIGUEL-PAGOLA, M., ALCARAZ, V. & POLVERINO, E. 2016. Short-term effects of three slow expiratory airway clearance techniques in patients with bronchiectasis: a randomised crossover trial. *Physiotherapy,* 102**,** 357-364.
HERRERO-CORTINA, B., MIGUEL-PAGOLA, M. S., FRANCIN-GALLEGO, M., SAEZ-PEREZ, J. A. & POLVERINO, E. 2019. Long-term efficacy of a home-based airway clearance programme to improve cough severity in patients with bronchiectasis: a randomised controlled trial. *Eur Respir J,* 54.
HESTER, K. L. M., RYAN, V., NEWTON, J., RAPLEY, T. & DE SOYZA, A. 2020. Bronchiectasis Information and Education: a randomised, controlled feasibility trial. *Trials,* 21, 331.
HIGGINS, J. P. T., THOMPSON, S. G., DEEKS, J. J. & ALTMAN, D. G. 2003. Measuring inconsistency in meta-analyses. *BMJ,* 327**,** 557-560.
HILL, J., RENTON, D., ABO-LEYAH, H., CRICHTON, M., FINCH, S., FARDON, T. & CHALMERS, J. 2015a. A single isolation of pseudomonas aeruginosa predicts poor quality of life and increased exacerbation frequency in adult bronchiectasis. *Eur Respir J,* 46**,** PA363.
HILL, J., RENTON, D., ABO-LEYAH, H., CRICHTON, M., FINCH, S., FARDON, T. & CHALMERS, J. 2015b. Idiopathic and post-infective bronchiectasis show no clinically important differences in outcomes or airway inflammatory profiles. *Eur Respir J,* 46**,** OA466.
HUNT, S. M. & MCEWEN, J. 1980. The development of a subjective health indicator. *Sociol Health Illn,* 2**,** 231-46.
JACQUES, PS., GAZZANA, MB., PALOMBINI, DV., BARRETO, SS., and DALCIN PTR. 2012. Six-minute walk distance is not related to quality of life in patients with non-cystic fibrosis bronchiectasis. *J Bras Pneumol,* 38(3), 346-355.
JAESCHKE, R., SINGER, J. & GUYATT, G. H. 1989. Measurement of health status. Ascertaining the minimal clinically important difference. *Control Clin Trials,* 10**,** 407-15.
JONES, P. W. 2005. St. George's Respiratory Questionnaire: MCID. *Chron Obstr Pulm Dis,* 2**,** 75-9.
JONES, P. W., HARDING, G., BERRY, P., WIKLUND, I., CHEN, W. H. & KLINE LEIDY, N. 2009. Development and first validation of the COPD Assessment Test. *Eur Respir J,* 34**,** 648.
JONES, P. W., QUIRK, F. H., BAVEYSTOCK, C. M. & LITTLEJOHNS, P. 1992. A Self-complete Measure of Health Status for Chronic Airflow Limitation: The St. George's Respiratory Questionnaire. *Am Rev Respir Dis,* 145**,** 1321-1327.
KEY, A., HOLT, K., SMITH, J. & WALKER, P. 2019. Objective cough count in bronchiectasis patients is reproducible and relates to quality of life. *Eur Respir J,* 54.
KHODOSH, E., POTEIKO, P., KRUTKO, V. & KHOROSHUN, D. 2015. Influence of inhaled

glucocorticosteroids on course of the bronchiectasis. *Eur Respir J,* 46**,** PA2963.

KIM, H. K., LEE, H., KIM, S. H., CHOI, H., LEE, J. H., LEE, J. S., LEE, S. W. & OH, Y. M. 2020. Validation of the Korean Version of the Bronchiectasis Health Questionnaire. *Tuberc Respir Dis,* 83, 228-233.

KON, S. S., CLARK, A. L., DILAVER, D., CANAVAN, J. L., PATEL, M. S., POLKEY, M. I. & MAN, W. D. 2013. Response of the COPD Assessment Test to pulmonary rehabilitation in unselected chronic respiratory disease. *Respirology,* 18**,** 974-7.

KUMAR, R., GULERIA, R., KHILNANI, G. C., MOHAN, A., MADAN, K., HADDA, V. & PANDEY, R. M. 2017. The effects of pulmonary rehabilitation in patients with non-cystic fibrosis bronchiectasis- A randomised controlled trial. *Eur Respir J,* 50**,** OA307.

LANZA, F. C., CASTRO, R. A. S., DE CAMARGO, A. A., ZANATTA, D. J. M., RACHED, S., ATHANAZIO, R., CUKIER, A., STELMACH, R. & DAL CORSO, S. 2018. COPD Assessment Test (CAT) is a Valid and Simple Tool to Measure the Impact of Bronchiectasis on Affected Patients. *Chronic Obstr Pulm Dis,* 15**,** 512-519.

LAVERY, K. A., O'NEILL, B., PARKER, M., ELBORN, J. S. & BRADLEY, J. M. 2011. Expert patient self-management program versus usual care in bronchiectasis: a randomized controlled trial. *Arch Phys Med Rehabil,* 92**,** 1194-201.

LECHTZIN, N., HILLIARD, M. E. & HORTON, M. R. 2013. Validation of the Cough Quality-of-Life Questionnaire in patients with idiopathic pulmonary fibrosis. *Chest,* 143, 1745-1749.

LEE, A., CECINS, N. & HILL, C. E. A. 2010. Utility of the chronic respiratory questionnaire in non-cystic fibrosis bronchiectasis. *Respirology,* 15**,** A78.

LEE, A. L., HILL, C. J., CECINS, N., JENKINS, S., MCDONALD, C. F., BURGE, A. T., RAUTELA, L., STIRLING, R. G., THOMPSON, P. J. & HOLLAND, A. E. 2014. The short and long term effects of exercise training in non-cystic fibrosis bronchiectasis--a randomised controlled trial. *Respir Res,* 15**,** 44-44.

LEE, B. Y., LEE, S., LEE, J. S., SONG, J. W., LEE, S.-D., JANG, S. H., JUNG, K.-S., HWANG, Y. I. & OH, Y.-M. 2012. Validity and Reliability of CAT and Dyspnea-12 in Bronchiectasis and Tuberculous Destroyed Lung. *Tuberc Respir Dis,* 72**,** 467-474.

LI, L., LI, Z., BI, J., LI, H., WANG, S., SHAO, C. & SONG, Y. 2020. The association between serum albumin/prealbumin level and disease severity in non-CF bronchiectasis. *Clin Exp Pharmacol Physiol,* 47, 1537-1544.

LIAW, M. Y., WANG, Y. H., TSAI, Y. C., HUANG, K. T., CHANG, P. W., CHEN, Y. C. & LIN, M. C. 2011. Inspiratory muscle training in bronchiectasis patients: a prospective randomized controlled study. *Clin Rehabil,* 25**,** 524-36.

LIU, J., ZHONG, X., HE, Z., WEI, L., ZHENG, X., ZHANG, J., BAI, J., ZHONG, W. & ZHONG, D. 2014. Effect of Low-Dose, Long-Term Roxithromycin on Airway Inflammation and Remodeling of Stable Noncystic Fibrosis Bronchiectasis. *Mediators Inflamm,* 2014**,** 708608.

LIU, D., MILROSS, M. & DWYER, T. 2019. Psychometric properties of the quality of life-bronchiectasis questionnaire in Australia. *Respirology,* 24, 145.

LIU, Y. & XU, J. 2020. Exhaled breath condensate pH as a biomarker of disease severity in non-cystic fibrosis bronchiectasis. *Am J Respir Crit Care Med,* 201.

LOEBINGER, M. R., WELLS, A. U., HANSELL, D. M., CHINYANGANYA, N., DEVARAJ, A., MEISTER, M. & WILSON, R. 2009. Mortality in bronchiectasis: a long-term study assessing the factors influencing survival. *Eur Respir J,* 34**,** 843-849.

MAA, S. H., TSOU, T. S., WANG, K. Y., WANG, C. H., LIN, H. C. & HUANG, Y. H. 2007. Self-administered acupressure reduces the symptoms that limit daily activities in bronchiectasis patients: pilot study findings. *J Clin Nurs,* 16**,** 794-804.

MACHADO, B. C., JACQUES, P. S., PENTEADO, L. P. & ROTH DALCIN, P. T. 2018. Prognostic Factors in Adult Patients with Non-Cystic Fibrosis Bronchiectasis. *Lung,* 196**,** 691-697.

MAGGE, A., ASHRAF, S., QUITTNER, A. L. & METERSKY, M. L. 2019. Quality of life in patients with bronchiectasis: a 2-year longitudinal study. *Ann Transl Med,* 7**,** 334.

MÁIZ, L., GIRÓN, R. M., PRATS, E., CLEMENTE, M. G., POLVERINO, E., CAÑO, S., CORDOVILLA, R., DORCA, J., PEÑALVER, C., BARANDA, F. & MARTÍNEZ-GARCÍA, M. A. 2018. Addition of hyaluronic acid improves tolerance to 7% hypertonic saline solution in bronchiectasis patients. *Ther Adv Respir Dis,* 12**,** 1753466618787385.

MANDAL, P., CHALMERS, JD., GRAHAM, C., HARLEY, C., SIDHU, MK., DOHERTY, C., GOVAN, JW., SETHI, T., DAVIDSON, DJ., ROSSI, AG., HILL, AT. 2014. Atorvastatin as a stable treatment in bronchiectasis: a randomised controlled trial. *Lancet Respir Med,* 2(6), 455-463.

MANDAL, P., MORICE, A. H., CHALMERS, J. D. & HILL, A. T. 2013. Symptoms of airway reflux predict exacerbations and quality of life in bronchiectasis. *Respir Med,* 107**,** 1008-1013.

MANDAL, P., SIDHU, M. K., KOPE, L., POLLOCK, W., STEVENSON, L. M., PENTLAND, J. L., TURNBULL, K., MAC QUARRIE, S. & HILL, A. T. 2012. A pilot study of pulmonary rehabilitation and chest physiotherapy versus chest physiotherapy alone in bronchiectasis. *Respir Med,* 106**,** 1647-54.

MARTÍNEZ-GARCÍA, M. A., PERPIÑÁ-TORDERA, M., ROMÁN-SÁNCHEZ, P. & SOLER-CATALUÑA, J. J. 2005. Quality-of-Life Determinants in Patients With Clinically Stable Bronchiectasis. *Chest,* 128**,** 739-745.

MARTÍNEZ-GARCÍA, M. A., PERPIÑÁ-TORDERA, M., ROMÁN-SÁNCHEZ, P. & SOLER-CATALUÑA, J. J. 2006. Inhaled steroids improve quality of life in patients with steady-state bronchiectasis. *Respir Med,* 100**,** 1623-32.

MARTÍNEZ GARCÍA, M. A., PERPIÑÁ TORDERA, M., ROMÁN SÁNCHEZ, P. & SOLER CATALUÑA, J. J. 2005. [Internal consistency and validity of the Spanish version of the St. George's respiratory questionnaire for use in patients with clinically stable bronchiectasis]. *Arch Bronconeumol,* 41**,** 110-7.

MCCULLOUGH, A. R., HUGHES, C. M., TUNNEY, M., ELBORN, J. S. & BRADLEY, J. M. 2011. P249 Assessment of health related quality of life (HRQoL) in non-cystic fibrosis bronchiectasis using a new disease-specific tool for measuring HRQoL: the Quality of Life-Bronchiectasis (QOL-B) questionnaire. *Thorax,* 66**,** A169-A169.

MCCULLOUGH, A. R., TUNNEY, M. M., QUITTNER, A. L., ELBORN, J. S., BRADLEY, J. M. & HUGHES, C. M. 2014. Treatment adherence and health outcomes in patients with bronchiectasis. *BMC Pulm Med,* 14**,** 107.

MCCULLOUGH, A. R., TUNNEY, M. M., STUART ELBORN, J., BRADLEY, J. M. & HUGHES, C. M. 2015. Predictors of adherence to treatment in bronchiectasis. *Respir Med,* 109**,** 838-845.

MCDONNELL, M. J., ALIBERTI, S., GOEMINNE, P. C., DIMAKOU, K., ZUCCHETTI, S. C., DAVIDSON, J., WARD, C., LAFFEY, J. G., FINCH, S., PESCI, A., DUPONT, L. J., FARDON, T. C., SKRBIC, D., OBRADOVIC, D., COWMAN, S., LOEBINGER, M. R., RUTHERFORD, R. M., DE SOYZA, A. & CHALMERS, J. D. 2016. Multidimensional severity assessment in bronchiectasis: an analysis of seven European cohorts. *Thorax,* 71**,** 1110-1118.

MCKEOUGH, Z. J., LARGE, S. L., SPENCER, L. M., CHENG, S. W. M. & MCNAMARA, R. J. 2020. An observational study of self-reported sedentary behaviour in people with chronic obstructive pulmonary disease and bronchiectasis. *Braz J Phys Ther,* 24**,** 399-406.

MESE, M., SAVCI, S., KAHRAMAN, B. O., TANRIVERDI, A., GUNDUZ, D. & SEVINC, C. 2020. The relationship of walking speed with clinical parameters in patients with non-cystic fibrosis bronchiectasis. *Eur Respir J,* 56.

MORSI, TS., GHOBASHY, S., YOUNIS, G. 2014. Quality of life and psychological disorders in Egyptian patients with chronic lung diseases: Clinico-physiological correlation. *Egypt J Chest Dis Tuberc,* 63(3), 731-742.

MUNOZ, G., BUXO, M., DE GRACIA, J., OLVEIRA, C., MARTINEZ-GARCIA, M. A., GIRON, R., POLVERINO, E., ALVAREZ, A., BIRRING, S. S. & VENDRELL, M. 2016. Validation of a Spanish version of the Leicester Cough Questionnaire in non-cystic fibrosis bronchiectasis. *Chron Respir Dis,* 13**,** 128-136.

MUNOZ, G., DE GRACIA, J., BUXÓ, M., ALVAREZ, A. & VENDRELL, M. 2018. Long-term benefits of airway clearance in bronchiectasis: a randomised placebo-controlled trial. *Eur Respir J,* 51**,** 1701926.

MUNOZ, G., OLVEIRA, C., MARTINEZ-GARCIA, M. A., GIRON, R., DE GRACIA, J., ALVAREZ, A., BUXO, M., POLVERINO, E., SABATER, G., VENDRELL, M. 2013 Validation of a Spanish version of the Leicester Cough Questionnaire in adult patients with non-cystic fibrosis bronchiectasis *Eur Respir J,* 2013**,** P5068.

NEWALL, C., STOCKLEY, RA., HILL, SL. 2005. Exercise training and inspiratory muscle training in patients with bronchiectasis. *Thorax*, 60, 943-948.

NICOLINI, A. & GRECCHI, B. 2019. Short-term comparative and follow-up study of two high frequency chest wall oscillation techniques in patients with bronchiectasis : A preliminary study. *Eur Respir J,* 54.

NICOLSON, C. H., STIRLING, R. G., BORG, B. M., BUTTON, B. M., WILSON, J. W. & HOLLAND, A. E. 2012. The long term effect of inhaled hypertonic saline 6% in non-cystic fibrosis bronchiectasis. *Respir Med,* 106**,** 661-7.

O'LEARY, C. J., WILSON, C. B., HANSELL, D. M., COLE, P. J., WILSON, R. & JONES, P. W. 2002. Relationship between psychological well-being and lung health status in patients with bronchiectasis. *Respir Med,* 96**,** 686-92.

OLVEIRA, C., OLVEIRA, G., ESPILDORA, F., GIRON, R. M., MUÑOZ, G., QUITTNER, A. L. & MARTINEZ-GARCIA, M. A. 2014a. Validation of a Quality of Life Questionnaire for Bronchiectasis: psychometric analyses of the Spanish QOL-B-V3.0. *Qual Life Res,* 23**,** 1279-92.

OLVEIRA, C., OLVEIRA, G., ESPILDORA, F., GIRÓN, R. M., VENDRELL, M., DORADO, A. & MARTÍNEZ-GARCÍA, M. 2014b. Mediterranean diet is associated on symptoms of depression and anxiety in patients with bronchiectasis. *Gen Hosp Psychiatry,* 36**,** 277-83.

OLVEIRA, G., OLVEIRA, C., DOÑA, E., PALENQUE, F. J., PORRAS, N., DORADO, A., GODOY, A. M., RUBIO-MARTÍNEZ, E., ROJO-MARTÍNEZ, G. & MARTÍN-VALERO, R. 2016. Oral supplement enriched in HMB combined with pulmonary rehabilitation improves body composition and health related quality of life in patients with bronchiectasis (Prospective, Randomised Study). *Clin Nutr,* 35**,** 1015-22.

OZALP, O., INAL-INCE, D., CAKMAK, A., CALIK-KUTUKCU, E., SAGLAM, M., SAVCI, S., VARDAR-YAGLI, N., ARIKAN, H., KARAKAYA, J. & COPLU, L. 2019. High-intensity inspiratory muscle training in bronchiectasis: A randomized controlled trial. *Respirology,* 24**,** 246-253.

OZALP, O., INAL-INCE, D., CALIK, E., VARDAR-YAGLI, N., SAGLAM, M., SAVCI, S., ARIKAN, H., BOSNAK-GUCLU, M. & COPLU, L. 2012. Extrapulmonary features of bronchiectasis: muscle function, exercise capacity, fatigue, and health status. *Multidiscip Respir Med,* 7**,** 3.

PANYARATH, P. 2016. Efficacy of roflumilast on exacerbations in patients with non-cystic fibrosis bronchiectasis: A preliminary randomized double-blind placebo-controlled trial. *Respirology,* 21**,** 126.

PATEL, S., COLE, A. D., NOLAN, C. M., BARKER, R. E., JONES, S. E., KON, S., CAIRN, J., LOEBINGER, M., WILSON, R. & MAN, W. D. C. 2019. Pulmonary rehabilitation in bronchiectasis: a propensity-matched study. *Eur Respir J,* 53**,** 1801264.

PATEL, S., BARKER, R., WALSH, J., POLGAR, O., NOLAN, C. & MAN, W. 2020. The five-repetition sit-to-stand test (5STS) in patients with bronchiectasis: validity and reponsiveness. *Eur Resp J,* 56.

PATHAK, P., DHARIWAL, M., WHALLEY, D., CRAWFORD, S. R. & ANGYALOSI, G. 2019. PRS64 Content validation of the respiratory symptoms subscale (RSS) of the quality of life questionnaire-bronchiectasis (QoL-B) in non-cystic fibrosis bronchiectasis. *Value Health,* 22, S883.

PEHLIVAN, E., NIKSARLıOĞLU, E. Y., BALCı, A. & KıLıÇ, L. 2019. The Effect of Pulmonary Rehabilitation on the Physical Activity Level and General Clinical Status of Patients with Bronchiectasis. *Turk Thorac J,* 20**,** 30-35.

PICCIRILLO, J. F., MERRITT, M. G., JR. & RICHARDS, M. L. 2002. Psychometric and clinimetric validity of the 20-Item Sino-Nasal Outcome Test (SNOT-20). *Otolaryngol Head Neck Surg,* 126**,** 41-7.

POLLEY, L., YAMAN, N., HEANEY, L., CARDWELL, C., MURTAGH, E., RAMSEY, J., MACMAHON, J., COSTELLO, R. W. & MCGARVEY, L. 2008. Impact of cough across different chronic respiratory diseases: comparison of two cough-specific health-related quality of life questionnaires. *Chest,* 134**,** 295-302.

POLVERINO, E., BLASI, F., RINGSHAUSEN, F., DE SOYZA, A., VENDRELL, M., GOEMINNE, P., BOERSMA, W., HAWORTH, C., MURRIS, M., DIMAKOU, K., LOEBINGER, M., MENENDEZ, R., TORRES, A., WELTE, T., WILSON, R., ELBORN, S., ALIBERTI, S. & CHALMERS, J. 2018. Determinants of quality of life in bronchiectasis using the quality of life bronchiectasis (QOL-B) questionnaire: data from the EMBARC registry. *Eur Respir J,* 52**,** OA4951.

POLVERINO, E., TRAVERSI, L., MARTINEZ-GARCIA, M., SHTEINBERG, M., BOSSIOS, A., DIMAKOU, K., PAGGIARO, P., GHILLAS, G., ADIR, Y., FERRI, S., SPANEVELLO, A., DENTE, F., ALIBERTI, S. & CHALMERS, J. 2020. Asthma as a co-morbidity and cause of bronchiectasis: data from the European Bronchiectasis Registry (EMBARC). *Eur Respir J,* 56.

PUMPHREY, E., KEIR, H., FONG, C., FINCH, S., FARDON, T. C., DICKER, A. J. & CHALMERS, J. D. 2017. Neutrophil Extracellular Traps Are Associated with Disease Severity in Bronchiectasis. *American Thoracic Society.* D94**,** A7302-A7302.

QI, Q., AILIYAER, Y., LIU, R., ZHANG, Y., LI, C., LIU, M., WANG, X., JING, L., LI, Y., Q, Q., Y, A., R, L., Y, Z., C, L., M, L., X, W., L, J., QI, Q., AILIYAER, Y., LIU, R., ZHANG, Y., LI, C., LIU, M.,

WANG, X., JING, L. & LI, Y. 2019. Effect of N-acetylcysteine on exacerbations of bronchiectasis (BENE): A randomized controlled trial. *Respir Res,* 20**,** 73-73.

QUITTNER, A. L., MARCIEL, K. K., SALATHE, M. A., O'DONNELL, A. E., GOTFRIED, M. H., ILOWITE, J. S., METERSKY, M. L., FLUME, P. A., LEWIS, S. A., MCKEVITT, M., MONTGOMERY, A. B., O'RIORDAN, T. G. & BARKER, A. F. 2014. A preliminary quality of life questionnaire-bronchiectasis: a patient-reported outcome measure for bronchiectasis. *Chest,* 146**,** 437-448.

QUITTNER, A. L., O'DONNELL, A. E., SALATHE, M. A., LEWIS, S. A., LI, X., MONTGOMERY, A. B., O'RIORDAN, T. G. & BARKER, A. F. 2015. Quality of Life Questionnaire-Bronchiectasis: final psychometric analyses and determination of minimal important difference scores. *Thorax,* 70**,** 12-20.

RAATS, D., DECAESTEKER, T., FOGELSTROM, M., DUPONT, L. & LORENT, N. 2019. Cross-sectional evaluation of serum and sputum calprotectin in bronchiectasis disease monitoring. *Eur Respir J,* 54.

RAJ, A. A., PAVORD, D. I. & BIRRING, S. S. 2009. Clinical cough IV:what is the minimal important difference for the Leicester Cough Questionnaire? *Handb Exp Pharmacol***,** 311-20.

RILEY, R. D., HIGGINS, J. P. T. & DEEKS, J. J. 2011. Interpretation of random effects meta-analyses. *BMJ,* 342**,** d549.

RINGSHAUSEN, F. C., SOKOL, P., BARTEN, G., DE ROUX, A., DIEL, R., KONWERT, S., WELTE, T., WITTE, M.-L., ZURAWSKI, A. & RADEMACHER, J. 2018. Quality of life is associated with increased symptom burden and markers of disease severity: data from the German Bronchiectasis Registry PROGNOSIS. *Eur Respir J,* 52**,** PA2670.

ROGERS, G. B., BRUCE, K. D., MARTIN, M. L., BURR, L. D. & SERISIER, D. J. 2014. The effect of long-term macrolide treatment on respiratory microbiota composition in non-cystic fibrosis bronchiectasis: an analysis from the randomised, double-blind, placebo-controlled BLESS trial. *Lancet Respir Med,* 2**,** 988-996.

ROSENTHAL, R. 1979. The file drawer problem and tolerance for null results. *Psychological Bulletin,* 86**,** 638-641.

ROWAN, S. A., BRADLEY, J. M., BRADBURY, I., LAWSON, J., LYNCH, T., GUSTAFSSON, P., HORSLEY, A., O'NEILL, K., ENNIS, M. & ELBORN, J. S. 2014. Lung clearance index is a repeatable and sensitive indicator of radiological changes in bronchiectasis. *Am J Respir Crit Care Med,* 189**,** 586-92.

SAHIN, H., VAROL, Y., ALSEL, N. & CIRAK, A. K. 2019. Which score is better in association with exercise capacity and QoL in noncystic fibrosis bronchiectasis patients? FACED or BSI? *Turk Thorac J,* 20, S85.

SAKA, S., OZALP, Ö., ÇALıK KÜTÜKÇÜ, E., İNAL İNCE, D., SAĞLAM, M., VARDAR YAĞLı, N., BOŞNAK GÜÇLÜ, M., ARıKAN, H., ÇÖPLÜ, L. & SAVCı, S. 2017. Comparison of physical activity level, activities of daily living, and depression between the patients with asthma and bronchiectasis. *Eur Respir J,* 50**,** PA2564.

SANTOS, D., DA SILVA GALLI, B. A., MOROLI, R. G., PEROSSI, J., PEROSSI, L., BADDINI-MARTINEZ, J. A. & GASTALDI, A. C. 2019. Is there a correlation between the bronchiectasis severity and health-related quality of life? *Eur Respir J,* 54.

SCHÜNEMANN, H. J., GRIFFITH, L., JAESCHKE, R., GOLDSTEIN, R., STUBBING, D. & GUYATT, G. H. 2003. Evaluation of the minimal important difference for the feeling thermometer and the St. George's Respiratory Questionnaire in patients with chronic airflow obstruction. *J Clin Epidemiol,* 56**,** 1170-6.

SHOEMARK, A., CANT, E., CARRETO, L., SMITH, A., ORIANO, M., KEIR, H. R., PEREA, L., CANTO, E., TERRANOVA, L., VIDAL, S., MOFFITT, K., ALIBERTI, S., SIBILA, O. & CHALMERS, J. D. 2019. A point-of-care neutrophil elastase activity assay identifies bronchiectasis severity, airway infection and risk of exacerbation. *Eur Respir J,* 53, 06.

SHTEINBERG, M., YAARI, N., STEIN, N., BENTUR, L., LIVNAT, G. & ADIR, Y. 2020. A randomized controlled trial of 4 weeks of airway clearance with oscillating positive end expiratory pressure device versus autogenic drainage in people with bronchiectasis. *Eur Respir J,* 56.

SIBILA, O., LASERNA, E., SHOEMARK, A., KEIR, H. R., FINCH, S., RODRIGO-TROYANO, A., PEREA, L., LONERGAN, M., GOEMINNE, P. C., CHALMERS, J. D., O, S., E, L., A, S., H.R, K., S, F., A, R.-T., L, P., M, L., P.C, G., SIBILA, O., LASERNA, E., SHOEMARK, A., KEIR, H. R., FINCH, S., RODRIGO-TROYANO, A., PEREA, L., LONERGAN, M., GOEMINNE, P. C., CHALMERS, J. D., O, S., E, L., A, S., H.R, K., S, F., A, R.-T., L, P., M, L., P.C, G. & D, C. J. 2019. Airway bacterial load and inhaled antibiotic response in bronchiectasis. *Am J Resp Crit*

Care Med, 200**,** 33-41.
SOKOL, P., G, B., A, D. R., R, D., S, K., T, W., A, Z. & J, R. 2019. Validation of the German translation of the Quality of Life-Bronchiectasis (QoL-B) questionnaire: Data from the German Bronchiectasis Registry PROGNOSIS. *Pneumologie,* 73.
SPECK, R. M., BENDER, R. H., GERLINGER, C. & FILONENKO, A. 2018. Psychometric Analyses of Pros in Bronchiectasis. *Value Health,* 21**,** S236-S237.
SPINOU, A., HOUSLEY, G., BIRRING, S. S., WILSON, R. & LOEBINGER, M. R. 2018. Cross-sectional comparison of the bronchiectasis-specific quality of life questionnaires. *Eur Respir J,* 52**,** PA2681.
SPINOU, A., LEE, K. K., SINHA, A., ELSTON, C., LOEBINGER, M. R., WILSON, R., CHUNG, K. F., YOUSAF, N., PAVORD, I. D., MATOS, S., GARROD, R. & BIRRING, S. S. 2017a. The Objective Assessment of Cough Frequency in Bronchiectasis. *Lung,* 195**,** 575-585.
SPINOU, A., SIEGERT, R. J., GUAN, W. J., PATEL, A. S., GOSKER, H. A.-O., LEE, K. K., ELSTON, C., LOEBINGER, M. R., WILSON, R., GARROD, R. & BIRRING, S. S. 2017b. The development and validation of the Bronchiectasis Health Questionnaire. *Eur Respir J,* 49**,** 1601532.
STOCKLEY, R., DE SOYZA, A., GUNAWARDENA, K., PERRETT, J., FORSMAN-SEMB, K., ENTWISTLE, N. & SNELL, N. 2013. Phase II study of a neutrophil elastase inhibitor (AZD9668) in patients with bronchiectasis. *Respir Med,* 107**,** 524-533.
SVENNINGSEN, S., GUO, F., MCCORMACK, D. G. & PARRAGA, G. 2017. Noncystic Fibrosis Bronchiectasis: Regional Abnormalities and Response to Airway Clearance Therapy Using Pulmonary Functional Magnetic Resonance Imaging. *Acad Radiol,* 24**,** 4-12.
SWIGRIS, J. J., KUSCHNER, W. G., JACOBS, S. S., WILSON, S. R. & GOULD, M. K. 2005. Health-related quality of life in patients with idiopathic pulmonary fibrosis: a systematic review. *Thorax,* 60**,** 588-594.
TERPSTRA, L. C., BIESENBEEK, S., ALTENBURG, J. & W.G., B. 2019. Aetiology and disease severity are among the determinants of quality of life in bronchiectasis. *Clin Respir J,* 13**,** 521-529.
TOMKINSON, JL., BRUTON, A. 2009. The 6-minute walk test for patients with bronchiectasis: comparison with normal predictive data. *Journal of the Association of Chartered Physiotherapists in Respiratory Care*. 41,16–21.
TONG, L., MILROSS, M. & DWYER, T. 2020. Quality of Life-Bronchiectasis (QOL-B) Questionnaire: A Sensitive Tool for An Australian Population with Bronchiectasis. *Respirology,* 25, 109.
TU, S.-P., FIHN, S. D., MCDONELL, M. B., SPERTUS, J. A. & STEELE, B. G. 1997. A New Self-Administered Questionnaire to Monitor Health-Related Quality of Life in Patients With COPD. *Chest,* 112**,** 614-622.
ÜZMEZOĞLU, B., ALTıAY, G., ÖZDEMIR, L., TUNA, H. & SÜT, N. 2018. The Efficacy of Flutter(®) and Active Cycle of Breathing Techniques in Patients with Bronchiectasis: A Prospective, Randomized, Comparative Study. *Turk Thorac J,* 19**,** 103-109.
VAROL, Y., SAHIN, H., AKSEL, N. & CIRAK, A. K. 2019. Which score is better in association with exercise capacity and QoL in noncystic fibrosis bronchiectasis patients? FACED or BSI? *Eur Respir J,* 54.
VISSER, S. K., BYE, P. T. P., FOX, G. J., BURR, L. D., CHANG, A. B., HOLMES-LIEW, C.-L., KING, P., MIDDLETON, P. G., MAGUIRE, G. P., SMITH, D., THOMSON, R. M., STROIL-SALAMA, E., BRITTON, W. J. & MORGAN, L. C. 2019. Australian adults with bronchiectasis: The first report from the Australian Bronchiectasis Registry. *Respir Med,* 155**,** 97-103.
WARE, J. E., JR. & SHERBOURNE, C. D. 1992. The MOS 36-item short-form health survey (SF-36). I. Conceptual framework and item selection. *Med Care,* 30**,** 473-83.
WATZ, H., NAGELSCHMITZ, J., KIRSTEN, A., PEDERSEN, F., VAN DER MEY, D., SCHWERS, S., BANDEL, T. J. & RABE, K. F. 2019. Safety and efficacy of the human neutrophil elastase inhibitor BAY 85-8501 for the treatment of non-cystic fibrosis bronchiectasis: A randomized controlled trial. *Pulm Pharmacol Ther,* 56**,** 86-93.
WILSON, C., JONES, P., O'LEARY, C., HANSELL, D., COLE, P. & WILSON, R. 1997b. Effect of sputum bacteriology on the quality of life of patients with bronchiectasis. *Eur Respir J,* 10**,** 1754-1760.
WILSON, C. B., JONES, P. W., O'LEARY, C. J., COLE, P. J. & WILSON, R. 1997a. Validation of the St. George's Respiratory Questionnaire in bronchiectasis. *Am J Respir Crit Care Med,* 156**,** 536-41.
WONG, C. C., P., LEWIS, C., CHANG, C., VANDAL, A., ZHEUNG, M., HAMED, M., TUFFERY, C., STOREY, L., JAYARAM, L., JONES, S. 2019. Oral Clonidine Improves Quality of Life in

Patients with Bronchiectasis. *Respirology,* 24**,** 101.

YANG, B., CHOI, H., LIM, J. H., PARK, H. Y., KANG, D., CHO, J., LEE, J. S., LEE, S. W., OH, Y. M., MOON, J. Y., KIM, S. H., KIM, T. H., SOHN, J. W., YOON, H. J. & LEE, H. 2019. The disease burden of bronchiectasis in comparison with chronic obstructive pulmonary disease: a national database study in Korea. *Ann Transl Med,* 7, 770.

YANG, B., JANG, H. J., CHUNG, S. J., YOO, S.-J., KIM, T., KIM, S.-H., SHIN, Y. M., KANG, H. K., KIM, J. S., CHOI, H. & LEE, H. 2020. Factors associated with bronchiectasis in Korea: a national database study. *Ann Transl Med,* 8, 1350.

YILDIZ, S., INAL-INCE, D., CALIK-KUTUKCU, E., VARDAR-YAGLI, N., SAGLAM, M., ARIKAN, H. & COPLU, L. 2018. Clinical Determinants of Incremental Shuttle Walk Test in Adults with Bronchiectasis. *Lung,* 196**,** 343-349.

ZANINI, A., AIELLO, M., ADAMO, D., CHERUBINO, F., ZAMPOGNA, E., SOTGIU, G., CHETTA, A. & SPANEVELLO, A. 2015a. Effects of Pulmonary Rehabilitation in Patients with Non-Cystic Fibrosis Bronchiectasis: A Retrospective Analysis of Clinical and Functional Predictors of Efficacy. *Respiration,* 89.

ZANINI, A., AIELLO, M., ADAMO, D., CASALE, S., CHERUBINO, F., DELLA PATRONA, S., RAIMONDI, E., ZAMPOGNA, E., CHETTA, A. & SPANEVELLO, A. 2015b. Estimation of minimal clinically important difference in EQ-5D visual analog scale score after pulmonary rehabilitation in subjects with COPD. *Respir Care,* 60, 88-95.

ZHONG, L., XIONG, Y., ZHENG, Z., LIU, N., HU, J., YANG, F. & CHEN, R. 2020. Effect of short-term inhalation of warm saline atomised gas on patients with non-cystic fibrosis bronchiectasis. *ERJ Open Res,* 6.

ZHOU, A., ZHOU, Z., PENG, Y., ZHAO, Y., DUAN, J. & CHEN, P. 2018. The role of CAT in evaluating the response to treatment of patients with AECOPD. *Chron Obstr Pulm Dis,* 13**,** 2849-2858.
