# Supplementary material for: Psychometrics of health-related quality of life questionnaires in bronchiectasis: a systematic review and meta-analysis
Source: Eur Respir J. 2021 Nov 11;58(5):2100025. doi: 10.1183/13993003.00025-2021 (PMC8581652; doi:10.1183/13993003.00025-2021)
Supplement: Supplementary file 2 [file ERJ-00025-2021.Shareable.pdf]

EUROPEAN RESPIRATORY JOURNAL
ORIGINAL RESEARCH ARTICLE
R.H. MCLEESE ET AL.

SHAREABLE PDF

Psychometrics of health-related quality of life questionnaires in bronchiectasis: a systematic review and meta-analysis

Rebecca H. McLeese [1], Arietta Spinou [2], Zina Alfahl [1,3], Michail Tsagris [4], J. Stuart Elborn [5], James D. Chalmers [6], Anthony De Soyza [7], Michael R. Loebinger [8], Surinder S. Birring [9], Konstantinos C. Fragkos [10], Robert Wilson [8], Katherine O'Neill [1,5,11] and Judy M. Bradley [1,5,11]

[1]The Wellcome Trust-Wolfson Northern Ireland Clinical Research Facility, School of Medicine, Dentistry and Biomedical Sciences, Queen's University Belfast, Belfast, UK. [2]Population Health Sciences, Faculty of Life Sciences and Medicine, King's College London, London, UK. [3]School of Pharmacy, Queen's University Belfast, Belfast, UK. [4]Dept of Economics, University of Crete, Rethymnon, Greece. [5]Wellcome-Wolfson Institute for Experimental Medicine, School of Medicine, Dentistry and Biomedical Sciences, Queen's University Belfast, Belfast, UK. [6]Scottish Centre for Respiratory Research, University of Dundee, Dundee, UK. [7]Respiratory Dept, Institute of Cellular Medicine, Newcastle University and Freeman Hospital, Sir William Leech Research Centre, Newcastle upon Tyne, UK. [8]Host Defence Unit, Royal Brompton Hospital, London, UK. [9]Centre for Human and Applied Physiological Sciences, School of Basic and Medical Biosciences, Faculty of Life Sciences and Medicine, King's College London, London, UK. [10]Division of Medicine, University College London, London, UK. [11]These two authors are co-senior authors.

Corresponding author: Judy M. Bradley (Judy.Bradley@qub.ac.uk)

Check for updates

Shareable abstract (@ERSpublications)
The psychometric properties of health-related quality of life questionnaires should inform the selection of patient-reported outcomes in bronchiectasis clinical trials https://bit.ly/3wQuSrm

Cite this article as: McLeese RH, Spinou A, Alfahl Z, *et al.* Psychometrics of health-related quality of life questionnaires in bronchiectasis: a systematic review and meta-analysis. *Eur Respir J* 2021; 58: 2100025 [DOI: 10.1183/13993003.00025-2021].

This single-page version can be shared freely online.

Copyright ©The authors 2021.

This version is distributed under the terms of the Creative Commons Attribution Licence 4.0.

This article has supplementary material available from erj.ersjournals.com

Received: 6 Jan 2021
Accepted: 2 April 2021

Abstract

Introduction Understanding the psychometric properties of health-related quality of life (HRQoL) questionnaires can help inform selection in clinical trials. Our objective was to assess the psychometric properties of HRQoL questionnaires in bronchiectasis using a systematic review and meta-analysis of the literature.

Methods A literature search was conducted. HRQoL questionnaires were assessed for psychometric properties (reliability, validity, minimal clinically important difference (MCID) and floor/ceiling effects). Meta-analyses assessed the associations of HRQoL with clinical measures and responsiveness of HRQoL in clinical trials.

Results 166 studies and 12 HRQoL questionnaires were included. The Bronchiectasis Health Questionnaire (BHQ), Leicester Cough Questionnaire (LCQ), Chronic Obstructive Pulmonary Disease (COPD) Assessment Test (CAT) and Medical Outcomes Study 36-item Short-Form Health Survey (SF-36) had good internal consistency in all domains reported (Cronbach's $\alpha \geqslant 0.7$) across all studies, and the Quality of Life-Bronchiectasis (QOL-B), St George's Respiratory Questionnaire (SGRQ), Chronic Respiratory Disease Questionnaire (CRDQ) and Seattle Obstructive Lung Disease Questionnaire (SOLQ) had good internal consistency in all domains in the majority of (but not all) studies. BHQ, SGRQ, LCQ and CAT had good test–retest reliability in all domains reported (intraclass correlation coefficient $\geqslant 0.7$) across all studies, and QOL-B, CRDQ and SOLQ had good test–retest reliability in all domains in the majority of (but not all) studies. HRQoL questionnaires were able to discriminate between demographics, important markers of clinical status, disease severity, exacerbations and bacteriology. For HRQoL responsiveness, there was a difference between the treatment and placebo effect.

Conclusions SGRQ was the most widely used HRQoL questionnaire in bronchiectasis studies and it had good psychometric properties; however, good psychometric data are emerging on the bronchiectasis-specific HRQoL questionnaires QOL-B and BHQ. Future studies should focus on the medium- to long-

term test–retest reliability, responsiveness and MCID in these HRQoL questionnaires which show potential in bronchiectasis.
